# A Versatile Biomimic Nanotemplating Fluidic Assay for Multiplex Quantitative Monitoring of Viral Respiratory Infections and Immune Responses in Saliva and Blood

**DOI:** 10.1002/advs.202204246

**Published:** 2022-10-17

**Authors:** Roozbeh Siavash Moakhar, Carolina del Real Mata, Mahsa Jalali, Houda Shafique, Alireza Sanati, Justin de Vries, Julia Strauss, Tamer AbdElFatah, Fahimeh Ghasemi, Myles McLean, Imman I. Hosseini, Yao Lu, Sripadh Guptha Yedire, Sahar Sadat Mahshid, Mohammad Amin Tabatabaiefar, Chen Liang, Sara Mahshid

**Affiliations:** ^1^ Department of Bioengineering McGill University Montreal Quebec H3A 0E9 Canada; ^2^ Biosensor Research Center Isfahan University of Medical Sciences Isfahan 81746‐73461 Iran; ^3^ Department of Medicine McGill University Montreal Quebec H4A 3J1 Canada; ^4^ Lady Davis Institute for Medical Research and McGill AIDS Centre Jewish General Hospital Montreal QC H3T 1E2 Canada; ^5^ Biological Sciences Sunnybrook Research Institute Sunnybrook Health Sciences Centre Toronto ON M4N 3M5 Canada; ^6^ Department of Genetics and Molecular Biology School of Medicine Isfahan University of Medical Sciences Isfahan 81746‐73461 Iran

**Keywords:** impedimetric biosensors, microfluidic devices, molecularly imprinted polymers, multiplexed testing, viral respiratory infection

## Abstract

The last pandemic exposed critical gaps in monitoring and mitigating the spread of viral respiratory infections at the point‐of‐need. A cost‐effective multiplexed fluidic device (NFluidEX), as a home‐test kit analogous to a glucometer, that uses saliva and blood for parallel quantitative detection of viral infection and body's immune response in an automated manner within 11 min is proposed. The technology integrates a versatile biomimetic receptor based on molecularly imprinted polymers in a core–shell structure with nano gold electrodes, a multiplexed fluidic‐impedimetric readout, built‐in saliva collection/preparation, and smartphone‐enabled data acquisition and interpretation. NFluidEX is validated with Influenza A H1N1 and SARS‐CoV‐2 (original strain and variants of concern), and achieves low detection limit in saliva and blood for the viral proteins and the anti‐receptor binding domain (RBD) Immunoglobulin G (IgG) and Immunoglobulin M (IgM), respectively. It is demonstrated that nanoprotrusions of gold electrodes are essential for the fine templating of antibodies and spike proteins during molecular imprinting, and differentiation of IgG and IgM in whole blood. In the clinical setting, NFluidEX achieves 100% sensitivity and 100% specificity by testing 44 COVID‐positive and 25 COVID‐negative saliva and blood samples on par with the real‐time quantitative polymerase chain reaction (*p* < 0.001, 95% confidence) and the enzyme‐linked immunosorbent assay.

## Graphical abstract

The authors present a nanostructured microfluidic electrochemical multiplexed device (NFluidEX) for parallel quantitative detection of viral proteins and antibodies in untreated saliva and whole blood within 11 minutes. This point‐of‐care platform is clinically and quantitatively validated with viral respiratory viruses to yield 100% sensitivity and 100% specificity against gold standard methods.

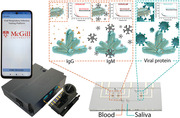

## 1 Introduction

Molecular diagnostic tests based on nucleic acid (e.g., ribonucleic acid (RNA)) detection have been recognized as the gold standard for accurate diagnosis of the viral respiratory infections.^[^ ^1^, ^2^, ^3^, ^4^, ^5^, ^6^ ^]^ However, RNA tests require costly equipment and trained personnel due to their lengthy protocols for sample preparation and testing. Particularly, the sample preparation for RNA extraction and detection is still hindered by a long turnaround time in centralized laboratory facilities.^[^ ^7^ ^]^ On the other hand, rapid antigen tests, such as BTNX Inc.’s Rapid Response Test have been developed to be more accessible and in the hands of individuals at home and in remote locations. However, antigen tests use nasal swabs as the targeted sample for detection, which are challenged with numerous false results with reported sensitivities as low as 50%.^[^ ^8^, ^9^ ^]^

An ideal test platform should address three challenges: 1) detecting the presence of particular viral invasion at the early stages of the infection from easily accessible body fluids such as saliva, 2) detecting the antibodies in response to the infection from whole blood, and 3) monitoring the efficacy of therapy once the patient is under treatment by quantifying both viral particles and specific antibodies. Indeed, saliva remains an unconventional choice in diagnostic testing of respiratory infection and particularly SARS‐CoV‐2.^[^ ^10^ ^]^ To date, the U.S. Food and Drug Administration (FDA) has solely issued approvals for saliva collection kits for at‐home testing of COVID‐19 through the self‐collection of saliva that is stabilized in a diluent buffer and shipped to a centralized facility for subsequent sample pretreatment and testing.^[^ ^11^, ^12^, ^13^ ^]^ When compared to self‐administered nasopharyngeal swabs (71%, 4.93 mean log copies mL^−1^), the self‐collected saliva (81%, 5.58 mean log copies mL^−1^) provides higher sensitivity when the diagnosis is performed in the first 5–10 days of infection by SARS‐CoV‐2.^[^ ^14^ ^]^ This is while the long virus incubation period of up to 14 days can accelerate the spread of the virus, where an average of 5–6 days is expected between incubation and symptom onset, thus prompting the need for more sensitive detection at early‐stages.^[^ ^15^ ^]^ However, the untreated saliva typically requires lengthy pretreatment protocols negatively affecting its potential. As such, the need for direct collection and on‐platform sample preparation is necessary to reduce the reliance on centralized laboratory facilities for diagnosis of viral respiratory infections.

The combination of molecular diagnostic and serological testing on a single platform improves the robustness of the result due to the heterogeneity in disease responses.^[^ ^16^ ^]^ Looking at the results of the last few years, testing via molecular diagnostics alone is shown to have a positive predictive agreement from 51.9% to 79.2%, likely due to viral load clearance from the upper respiratory tract over time. Meanwhile, a combined approach with tandem serology testing increases the positive detection rate to 98.6 to100%,^[^ ^17^, ^18^ ^]^ allowing for more reliable responses during the acute and convalescent phase of infection. In addition, the multiplex detection of immunoglobulin G (IgG) and immunoglobulin M (IgM) antibodies enables the serosurveillance of current and past infections based on the temporal prevalence of the immunoglobulins in response to infection; sequential seroconversion is confirmed to occur first in IgM followed by IgG in early infection, with waning levels of IgM and high levels of IgG during late infection.^[^ ^19^, ^20^ ^]^ Nonetheless, the diagnostic community has found it highly challenging to achieve the accuracy for parallel diagnosis and serological testing in one single attempt from the easily accessible body fluids.

**Figure 1:**
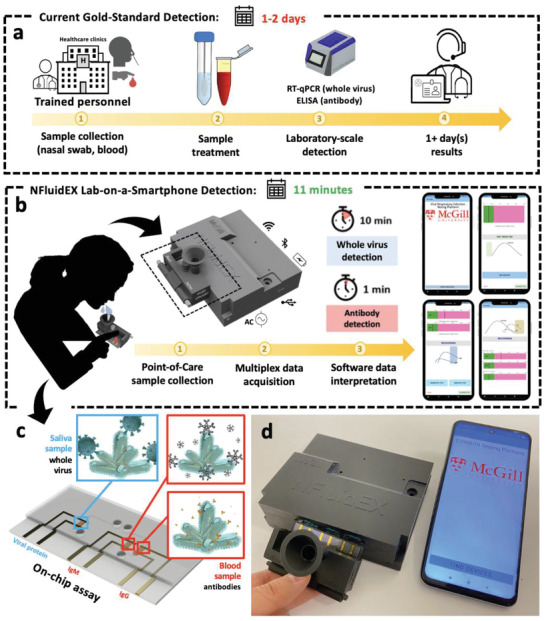
Schematic representation of the NFluidEX. a) The gold standard methods for diagnosis and serology testing of viral respiratory infection. (b) The NFluidEX device for rapid identification of current and past infections. c) The electrochemical microfluidic device with on‐chip assays for the specific detection of whole viral particles in saliva and antibodies in blood. (d) Real image of the NFluidEX quantitative diagnosis and serology testing.

Here, we propose a novel paradigm for viral respiratory infection diagnosis and serological testing via parallel analysis of untreated saliva and whole blood for quantitative detection of viral particles and specific antibodies (IgG and IgM) via the nanostructured microfluidic electrochemical multiplexed (NFluidEX) device. The NFluidEX features a gold nano/micro islands (NMIs) sensor ^[^ ^21^, ^22^, ^23^, ^24^ ^]^ within the electrochemical biosensor and a newly developed biomimetic receptor based on molecularly imprinted polymers (MIPs) with proven chemical stability, scalability, and simple adaptability compared to affinity‐based assays.^[^ ^25^, ^26^, ^27^, ^28^ ^]^ This has ushered a new standard for electrochemical MIP‐based biosensors toward a translational capacity for targets with various sizes and geometries in complex biofluids for selective biosensing.^[^ ^29^, ^30^, ^31^ ^]^ In case of the NFluidEX, a thin layer of MIP monomer was electropolymerized in the presence of a target template on the NMIs sensor, in which the template was then removed to leave built‐in recognition sites to rebind to the target and generate a strong electrical signal.^[^ ^32^, ^33^ ^]^ The MIP biomimetic receptors are tunable with the morphological and physical characteristics of the target and can be rapidly adapted for various viral subtypes and variants of concern. The combination of the assay with a multiplexed microfluidic sample delivery and an automated custom‐made potentiostat resulted in a rapid (total 11 min: 10 min incubation and 1 min signal transduction) easy‐to‐use quantitative low‐cost NFluidEX as a home‐test kit, analogous to a glucometer (**Figure** **1**). The NFluidEX possesses a sample cartridge for the collection of untreated samples directly from the user by placing a blood sample from a finger prick and a saliva sample from a self‐collection funnel. The serology testing from blood is performed in the device with a 1 min incubation time, followed by the saliva testing with a 10 min incubation time. A smartphone application is coupled to receive and analyze signals via integrated Wi‐Fi and Bluetooth controllers and displays the results on the smartphone screen within 1 min. The NFluidEX achieved a low limit of detection (LOD) and clinically relevant sensitivity and specificity for the detection of viral particles and specific antibodies that robustly correlate with real‐time quantitative polymerase chain reaction (RT‐qPCR) and enzyme‐linked immunosorbent assay (ELISA), respectively. This approach can be rapidly adapted and reconfigured to address diverse user needs and detect different viruses. As the proof of concept and due to the availability of testing samples in the past two years, we tested the NFluidix for Influenza A H1N1 as well as for SARS‐CoV‐2 and its Alpha B.1.1.7, Delta B.1.617.2, and Omicron B.1.1.529 variants of concern (VOCs) with clinically relevant sensitivities and specificities.

## 2 Results and Discussion

### 2.1 The NFluidEX with the Biomimic NMIs/MIPs Detection Assay

The nanostructured microfluidic electrochemical multiplexed (NFluidEX) device operates based on two main compartments for parallel analysis of the saliva and blood samples: the impedimetric signal transduction module and the multiplexed fluidic test assays connected to the sample collection cartridge (Figure 1). The signal transduction panel is a portable cost‐effective battery‐operated electrochemical workstation that consists of a printed electrical circuit board and a Bluetooth wireless connection to convert the impedimetric signal of the test assay to a quantifiable readout on a smartphone via an Android application within 1 min (Figures S1–S4, Supporting Information). A single‐use sample collection cartridge embedded with a 3‐channel multiplexed fluidic assay allows for easy self‐collection of the saliva and blood by a lay‐user. Patient blood samples can be collected by a self‐administered lancing device (≈3 µL), while patient saliva can be obtained via an attached self‐collection funnel (≈700 µL). The parallel fluidic channels avoid cross contamination between blood and saliva samples (more details in the Supporting Information; Figures S5–S8, Table S1, and Movie S1, Supporting Information). The complete NFluidEX prototype enables an easy‐to‐use diagnosis and serology testing device for viral respiratory infections (Movie S2, Supporting Information).

**Figure 2:**
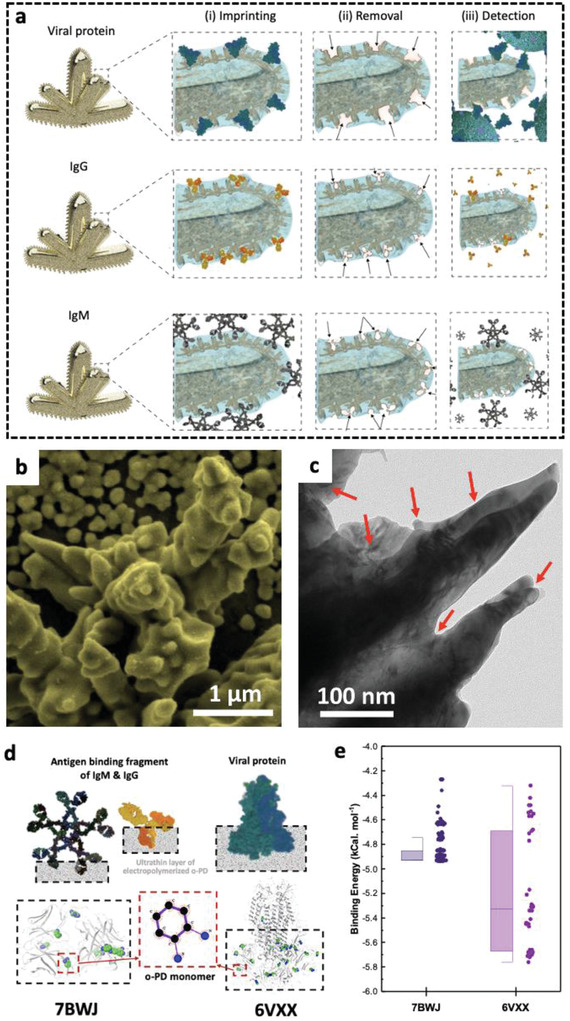
Design of the biomimic NMIs/MIPs detection assay within NFluidEX. (a) The formation of rapidly adaptable MIPs templates using viral proteins (top), IgG antibody (middle), and IgM antibody (bottom) for (i) imprinting the polymeric thin film on the nanorough surface of NMI electrodes and (ii) their removal to form the empty geometrical shapes in polymer (iii) for selective detection, (b) scanning electron microscopy image of the gold NMIs, and (c) transmission electron microscopy image of the biomimic NMIs/MIPs, (d) Molecular docking studies of the interaction between o‐PD monomers and amino acids of template proteins to show regions of high binding affinities, (e) quantified binding affinity between o‐PD and amino acids of the template proteins (IgG/IgM [7BWJ] and viral protein [6VXX]).

The fluidic assay consists of three biomimic NMI/MIP detection chambers, where the viral proteins (the viral spike proteins (SP) of SARS‐CoV‐2 or the viral haemagglutinin surface protein of Influenza A H1N1), and specific antibodies (here the anti‐receptor binding domain (RBD) antibodies, IgG‐RBD and IgM‐RBD) are separately imprinted in the ortho‐phenylenediamine (o‐PD) polymeric layer of each chamber, respectively, with a thickness of 5–10 nm nested on the nanorough surface of 1 µm sized NMI electrodes. Each chamber mimics the spatial configuration of the head domain of the targets, which enables the specific simultaneous detection of whole viral particles in saliva, and antibodies in blood (**Figure** **2**a; and Figures S9–S12, Supporting Information).

The sensitivity of the biomimic NMI/MIP assay strongly depends on the structural configuration and enhanced electroactivity of NMI structures. The test chamber design confers an ≈6 times enhanced electrochemical signal response compared to the signal of a flat electrode, arising from the large surface area and uniform distribution of micro‐dimensional gold NMI surface (Figure 2b).^[^ ^21^, ^22^, ^23^ ^]^ The electrochemically induced defects and dislocations (shown with arrows in Figure 2c) in the nanoprotuberance of a single NMI enhance the signal sensitivity through elevated aspect ratio geometries,^[^ ^34^ ^]^ structural isotropy,^[^ ^35^, ^36^ ^]^ entropy, and surface energy (details in Figure S9, Supporting Information). Electrochemical patterning of the surface with NMIs gives rise to an enhanced localized electrical field confinement at the edges of the nanoprotuberance for enhanced electrocatalytic activity at the surface of the electrode.^[^ ^37^, ^38^ ^]^ The appendage microstructure grown on the conductive base that transfers a concentrated electric field, is enhanced locally along the edges and nanoprotuberances, which gives rise to a collective plasmon oscillation. The enhanced localized electric field expedites the charge transference by reducing the electron transfer barriers and consequently, enhances the signal transduction that positively affects the detection sensitivity for low traces of the target analyte.^[^ ^39^, ^40^ ^]^

The selectivity of the biomimic NMI/MIP recognition assay relies on the formation of binding sites in the polymeric thin films imprinted by viral proteins and antibodies that is central to selective recognition in saliva and blood, respectively. We used a thin layer (5–10 nm) of o‐PD polymer to interact with protein and antibody entities of the virus to record their spatial molecular configuration. We benchmarked the proficiency of templating the polymer layer with the virus entities by focusing on SARS‐CoV‐2 and investigating the affinity of the antigen‐binding fragment of the IgG‐RBD and IgM‐RBD (7BWJ) and the SP (6VXX) from Protein Data Bank (PDB), with the o‐PD polymer layer using molecular docking (MD) simulations to determine the most favourable binding sites (Figure 2d). The amino acids on the template protein conjugate with o‐PD to form recognition sites and to impart chemical functionality into the binding pockets. These preferential binding sites are determined based on binding energy quantification (Figure 2e). For the 6VXX SP, preferential binding of the o‐PD monomers is primarily in the head region near the RBD, thereby demonstrating monomer competition for stabilizing interactions in a region with a high binding affinity of −5.22 kcal mol^−1^.^[^ ^41^ ^]^ Similarly, quantification of free energy minimization for the 7BWJ antigen‐binding fragment showed competitive stabilization with a binding affinity of −4.85 kcal mol^−1^. The directional interactions of o‐PD monomers with the head region binding domain of the proteins allow for partial confinement of proteins in the polymer, which is essential for their removal to form the empty geometrical shapes in polymer for selective detection (simulation conditions summarized in Tables S2 and S3, Supporting Information). Formation of the selective geometrical pockets and template protein removal is confirmed by 3D surface topography, demonstrating a rough, and indented surface casted with respect to the size and shape of the template proteins (Figure S13, Supporting Information) for the selective nanoimprinted geometric sites.

### 2.2 Analytical Performance of the NFluidEX

The NFluidEX enables a multiplex parallel analysis of saliva samples for whole virus detection via its viral protein, and blood samples for serology testing of IgG and IgM antibodies. The analytical assessment of NFluidEX is evaluated based on the impedance magnitude within the biological concentration ranges (ng mL^−1^ to µg mL^−1^)^[^ ^42^ ^]^ in buffer solution and in body fluids including saliva, plasma, and blood. All signal recordings were obtained by a potentiostat/galvanostat module with a potential amplitude of 10 mV and optimized to indicate the correctly adjusted impedimetric signal collection parameters for patient samples (detailed discussion in the Supporting Information).

**Figure 3:**
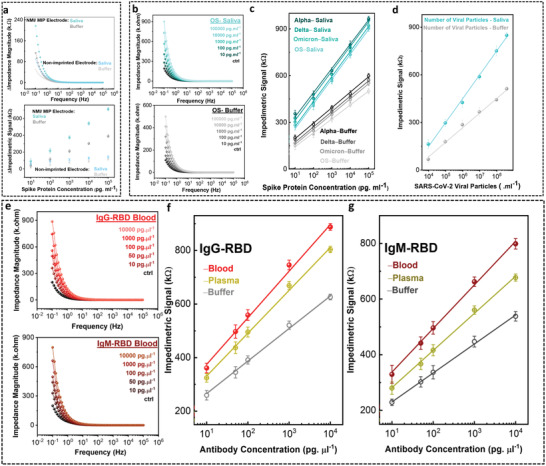
Analytical sensitivity performance metrics. (a) The impedimetric signal transduction of SARS‐CoV‐2 SP on NMI/MIP electrode imprinted with the SP head domain compared with NMI/NIP (non‐imprinted polymer electrode) in buffer (black) and saliva (cyan). (b) The impedance magnitude of SARS‐CoV‐2 SP on NMI/MIP electrode in different concentrations in saliva and buffer. The correlated linear relation of the impedimetric signal as a function of SP concentration in saliva and buffer belonging to (c) original strain, Alpha B.1.1.7, Delta B.1.617.2, and Omicron B.1.1.529 variants and (d) heat‐inactivated SARS‐CoV‐2 viral particles. (e) The impedance magnitude of SARS‐CoV‐2 IgG‐RBD (light red) and IgM‐RBD (dark red) antibodies on NMI/MIP electrode in whole blood. The correlated linear relation of the impedimetric signal as a function of (f) IgG‐RBD and (g) IgM‐RBD concentration in whole blood (red), plasma (yellow), and buffer (gray). Data shows mean values ± standard deviation (n = 3).

The working principle of the NMI/MIP signal transduction is based on the detection of an increase in impedance magnitude of the spectroscopic signal upon interaction of the electrodes with the specific domains of viral proteins or antibodies in the designated chambers. As a result, electron transfers between electrodes and redox active species in electrolytes are blocked, resulting in changes in impedance magnitude. An incubation time of 10 min for viral proteins in saliva, and 1 min for IgG and IgM in whole blood is required for the optimal performance of the assays (Figure S14, Supporting Information). The sensitive signal transduction at a low concentration of virus (10 pg mL^−1^) is achieved due to the NMI/MIP electrodes harbouring the binding sites. This is evident by comparing the impedimetric signal of viral protein on the biomimic NMI/MIP test assay with imprinted binding sites, with that of NMI/nonimprinted polymer (NIP) electrode (without imprinted binding sites) (**Figure** **3**a). The negligible impedimetric magnitude changes in both buffer and human saliva on the NMI/NIP electrode confirm the lack of imprinted binding sites in the nonconductive o‐PD layer compared to the NMI/MIP. Continuous potential cycling over 100 cycles on the electrochemical NMI, NMI/MIP, and NMI/NIP show little changes in the impedance magnitude, demonstrating the stability of the electrochemical biosensor (Figure S15, Supporting Information).

In both buffer and saliva media, and for all tested concentrations, the NMI/MIP electrodes bearing the binding sites generate a sensitive differentiable signal. In case of SARS‐CoV‐2, for the viral protein, the increase in the impedance signal was positively correlated to the increase in the concentration of the virus (Figure 3b; and Figure S16, Supporting Information). The frequency in which the signal transduction is performed tremendously affects the signal resolution at low concentrations of analytes (0.1–10^5^ Hz). As such, extenuating the test frequency to 0.1 Hz, led to an increased signal resolution capable of fully distinguishing concentrations as low as 10 pg mL^−1^. Higher impedance magnitude responses are observed in saliva compared to buffer solution due to higher biofluid viscosity, mucin‐related matrix effects and the presence of additional interferent proteins,^[^ ^43^ ^]^ resulting in an overall higher limit of detection. Nonetheless, the signal difference remained negligible and within the same order of magnitude, so untreated biofluids did not generate false readouts on the NMI/MIP assay (Figure S17, Supporting Information).

The impedimetric signal increased linearly with the concentration of the SARS‐CoV‐2 SP within the range of 10–10^5^ pg mL^−1^ for the original strain Alpha B.1.1.7, Delta B.1.617.2, and Omicron B.1.1.529 variants, after a 10 min incubation time in buffer (Figure 3c). Similarly, spiked saliva samples demonstrated a linear signal behavior with respect to increase of concentration (*R* ^2^ = 0.99), with negligible interference of saliva media. The calculated LOD of the NFluidEX is in the low pg mL^−1^ ranges for the detection of SARS‐CoV‐2 SP (Original strain: 5.89 pg mL^−1^; Alpha variant: 6.48 pg mL^−1^; Delta variant: 8.13 pg mL^−1^; Omicron variant: 7.62 pg mL^−1^; IgG‐RBD and IgM‐RBD: 3–7 pg µL^−1^, Table S4, Supporting Information), and its wide linear range (10–10^5^ pg mL^−1^) stands out when compared to other literature reports and COVID‐19 Emergency Use Authorization (EUA) medical devices, particularly with its superior application in testing the untreated saliva in a short time‐window (Tables S5–S7, Supporting Information).

Translating the performance of the test assay based on viral load compared to the concentration of other viral entities is advantageous by enabling detection without lysis, isolation or concentration of the entities.^[^ ^4^ ^]^ To assess the biomimic NMI/MIP test assay for the detection of whole viral particles, we calibrated the impedimetric signal based on the tested heat‐inactivated SARS‐CoV‐2 particles in physiological concentrations in both buffer and saliva (Figure S18, Supporting Information). The imprinted polymer assay remained amendable over a wide linear range from 9.60 × 10^3^ to 3.84 × 10^8^ number of viral particles mL^−1^ (Figure 3d), which is comparable with physiologically relevant viral loads in saliva.^[^ ^44^ ^]^ The LOD of 948.4 number of viral particles mL^−1^ lies sufficiently below the requirements by the World Health Organization, thereby demonstrating the applicability of the NFluidEX for early‐stage diagnosis from saliva.^[^ ^45^, ^46^ ^]^

In parallel, the NFluidEX was tested for the detection of both IgG‐RBD and IgM‐RBD antibodies to determine the ability of the device in serology testing. In the designated chambers, the biomimic NMIs/MIPs were specifically fabricated for the serological detection of SARS‐CoV‐2 specific antibodies. The impedimetric signal of antibodies in the concentration range of 10–10^4^ pg µL^−1^ was assessed in spiked buffer, undiluted human plasma, and whole blood, demonstrating an increasing trend with respect to the concentration of IgG‐RBD and IgM‐RBD (Figure 3e). A linear relationship between the impedimetric signal and logarithm value of the SARS‐CoV‐2 antibody concentration over the range of 10–10^4^ pg µL^−1^ was found in buffer, undiluted human plasma, and whole blood, respectively (Figure 3f,g; and Figure S19, Supporting Information). The analytical performance of the multiplexed test assay with whole blood is comparable to that of plasma and buffer, with a LOD as low as 3.13 pg µL^−1^ (Table S4, Supporting Information), confirming the serosurveillance capability of the NFluidEX directly in whole blood.^[^ ^47^, ^48^ ^]^

To explore the efficacy of NfluidEX for selective detection of SARS‐CoV‐2 SP in saliva, and both IgG‐RBD and IgM‐RBD in blood, we obtained the impedimetric signal of SARS‐CoV‐2 in buffer and saliva compared to the signals of other viral infections that can interfere with SARS‐CoV‐2 detection due to similarities in shape, size, and molecular composition. These include the severe acute respiratory syndrome coronavirus 1 (SARS‐CoV‐1), the human coronavirus 229E (HCoV‐229E), the Middle East respiratory syndrome coronavirus (MERS‐CoV), and Influenza A H1N1 (more details in the Supporting Information).

**Figure 4:**
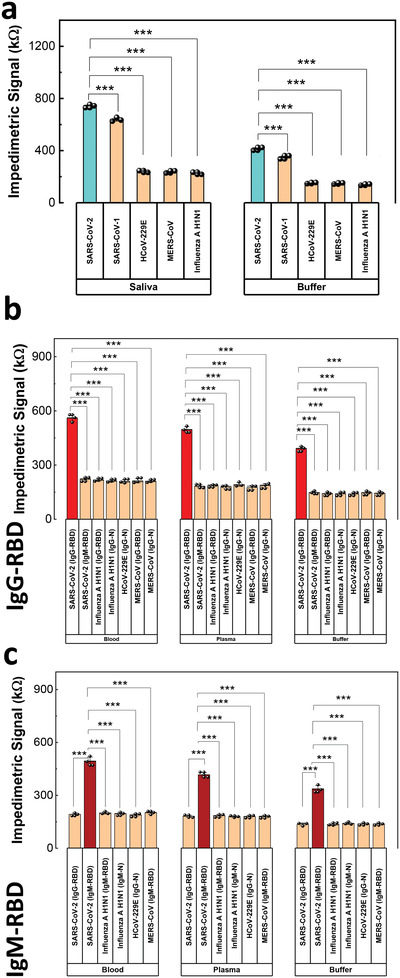
Analytical selectivity performance metrics. (a) Quantification of the cross‐reactivity of the SARS‐CoV‐2 SP as opposed to different viral SPs. Quantification of the cross‐reactivity of the SARS‐CoV‐2 antibodies (b) IgG‐RBD and (c) IgM‐RBD compared with different viral IgG and IgM antibodies, *** *p* < .001. Data shows mean values ± standard deviation (n = 3).

The impedimetric signal from biomimic NMIs/MIPs assay in the NFluidEX device imprinted with SARS‐CoV‐2 SP demonstrated a higher value towards its matching protein (SARS‐CoV‐2 SP) compared to the SPs from other tested viruses (**Figure** **4**a). We performed a null comparison between the results using one‐way analysis of variance (ANOVA) with post hoc Holm‐Sidak's test. ANOVA demonstrated an overall significant difference among the target SARS‐CoV‐2 SP in the NMI/MIP test assay and other viral SPs (*p* < .001, Table S8, Supporting Information), suggesting a low nonspecific binding of other viral SPs with the biomimic NMIs/MIPs imprinted with SARS‐CoV‐2 SP. The selectivity was tested at a lower concentration within the linear range of detection (Figure S20, Supporting Information) rendering similar selective signal magnitude, which demonstrates that over the concentration range, the presence of the signal of SARS‐CoV‐2 is still significantly higher than the others. This indicates low cross‐reactivity of other viral SPs with the NFluidEX saliva test assay. Notably, SARS‐CoV‐1 was detectable yet distinguishable on the SARS‐CoV‐2 SP imprinted assay, indicating that a past virus with high sequence and structural homology will unsurprisingly bind the assay; similar cross‐reactivity has been reported on both proposed and commercial clinical tests.^[^ ^49^, ^50^ ^]^

To investigate the versatility of the test response towards SARS‐CoV‐2, SPs from a series of its variants, Alpha B.1.1.7, Delta B.1.617.2, and Omicron B.1.1.529 were tested similarly on the original strain SP imprinted NMI/MIP assay, demonstrating an adaptable impedimetric response to identify the viral SP from different variants (Figure S21, Supporting Information).^[^ ^49^, ^50^ ^]^ Sequence and structural similarity comparisons are presented in Tables S9 and S10, Supporting Information, demonstrating a relationship between selective response and the homology between protein structures.^[^ ^49^ ^]^

We investigated the selectivity of the NFluidEX towards SARS‐CoV‐2 IgG‐RBD and IgM‐RBD antibodies over those of other similar viruses. Similarly, the NMI/MIP test chambers imprinted SARS‐CoV‐2 IgG‐RBD and IgM‐RBD demonstrated higher impedimetric signals towards their targets with minimal cross‐reactivity (Figure 4b–c). We performed a null comparison between the results using one‐way ANOVA with post hoc Holm–Sidak's test. There was negligible cross‐binding for Influenza A H1N1 IgG‐N, Influenza A H1N1 IgG‐RBD, HCoV‐229E IgG‐N, MERS‐CoV IgG‐N, MERS‐CoV IgG‐RBD, Influenza A H1N1 IgM‐N, Influenza A H1N1 IgM‐RBD, and MERS‐CoV IgM‐RBD in the SARS‐CoV‐2 IgG‐RBD and IgM‐RBD imprinted assays (*p* < .001, Tables S11 and S12, Supporting Information). In particular, negligible cross‐reactivity was detected for SARS‐CoV‐2 IgM‐RBD on SARS‐CoV‐2 IgG‐RBD assay and SARS‐CoV‐2 IgG‐RBD on SARS‐CoV‐2 IgM‐RBD assay (comparing the first and second columns in Figure 4b,c).

The selectivity of the assay toward the imprinted target was further tested with a determined concentration of the target and analogous viral particles within the linear range response of the assay, demonstrating a high selectivity at even lower concentrations (Figure S22, Supporting Information). In a comparative study, to confirm the higher accuracy in the readout when targeting the anti‐RBD antibodies versus antinucleocapsid antibodies (IgG‐N and IgM‐N), the IgG‐RBD and IgM‐RBD entities were imprinted in the assay and tested with similar conditions (Figure S23a,b, Supporting Information). As predicted, an attenuated positive impedimetric signal was observed for IgG‐N and IgM‐N antibodies compared to the statistically higher response from anti‐RBD antibodies (*p* < .001, details in the Supporting Information). The anti‐RBD assay was tested with Delta B.1.617.2 anti‐RBD antibodies (Figure S23c, Supporting Information) and a nearly identical impedimetric signal was obtained, indicating that the original strain assay can be used to detect antibodies belonging to variants of concern.

To demonstrate the broad applicability of the NFluidEX test assay, we tested the biomimic NMI/MIP assay for Influenza A H1N1 virus. The assay was imprinted following the same protocol with the viral haemagglutinin surface protein of Influenza. Electrochemical sensing demonstrated a linearly increasing impedance magnitude over the varied concentration of viral protein. A similar linear trend over a wide linear range from 10 to 10^5^ pg mL^−1^ at a low LOD was observed in both buffer and saliva (Figure S24a–c, Supporting Information). To validate the haemagglutinin imprinted geometric sites for the detection of whole Influenza viral particles, we challenged the assay with heat‐inactivated viral particles and observed linearly increasing impedimetric signal from 6.44 × 10^6^ to 2.58 × 10^9^ number of viral particles mL^−1^ at a low LOD in buffer and saliva (Figure S24d–f, Supporting Information).

The affinity of nanoimprinted assay is an essential component for the effective performance of the NFluidEX. To validate the affinity of the NMI/MIP assay, a series of experiments were conducted with the conventional method for affinity characterization, surface plasmon resonance (SPR). The SPR experiments were used to further study the difference between NMI/MIP and NMI/NIP (NMI/nonimprinted polymer electrode (without imprinted binding sites) assays. The change in optical response was denoted by right‐shifted wavelengths and was proportional the fraction of analyte bound, thereby inversely proportional to the dissociation constant. As expected, upon the introduction of the analyte, the NIP assay shows a slight response that may be attributed to nonspecific binding, while the MIP assay demonstrated a strong change in wavelength for all protein targets over their respective linear ranges, which was fitted using the nonlinear Hill equation (Equation (S3), Figures S25–S25, Tables S13–S14, Supporting Information) to determine the dissociation constant (*K*_d_). A comparison with current literature, *K*_d_ values show that the subnanomolar responses lies within a relevant range (Tables S15 and S16, Supporting Information). The *K*_d_ was calculated to be 3.17 pm for the SARS‐CoV‐2 SP, and 0.32 and 0.57 nm for the SARS‐CoV‐2 IgG‐RBD and IgM‐RBD, respectively, which suggests a strong affinity between the target proteins and the nano‐imprinted NMI/MIP, thus supporting the stability of the binding surface.^[^ ^51^ ^]^ In addition, these subnanomolar values correlate with the molecular docking simulation, suggesting a higher binding affinity for the SP as compared to the antigen fragment binding of the antibodies.

The imprinting factors (IFs) were calculated by the ratio of MIP to NIP response (Equation (S4), Supporting Information), with higher values indicating greater binding to the NMI/MIP assay as opposed to the NMI/NIP assay and lower values suggest greater potential for nonspecific binding. We observed IFs up to 5, indicating low potential for nonspecific binding; the NMI/MIP IFs are comparable between viral proteins, antibodies, and whole viral particles in buffer and in untreated saliva, plasma, and whole blood (Tables S17 and S18, Supporting Information), thus demonstrating the translational potential of the NFluidEX assay for both large and small biomolecules.

In addition, impedimetric sensing of saliva and blood electrolyte was performed on commercial gold screen printed electrodes (SPE), flat gold platforms, and gold NMIs. In the complex biofluids, the sensing linear range and LOD results were significantly better on the gold NMIs compared to commercial screen‐printed electrodes (Figure S29, Supporting Information), thereby indicating that the enhanced nanostructure presents a stable structure for subsequent electropolymerization and sensing of complex biofluids. The greatest change in impedimetric response was similarly noted in the NMI/MIP electrode compared to the SPE and flat gold counterparts, thus making it a suitable and stable choice for a biosensor in complex media. The core–shell NMI/MIP assay in complex biofluids has a limit of detection at least one order of magnitude lower than the flat gold and screen‐printed electrode counterparts, in addition to a wider linear range and higher sensitivity (Table S19, Supporting Information).

### 2.3 Validation of the NFluidEX Device with COVID‐19 Patient Samples

Real human biofluids are highly complex, so the evaluation of the NFluidEX device with patient samples can determine its efficacy when challenged with untreated saliva and whole blood. To demonstrate the applicability of the NFluidEX for clinical decision making, we analysed 34 COVID‐19‐positive saliva samples in contrast to 17 COVID‐19‐negative saliva samples, while simultaneously testing 10 COVID‐19‐positive patient blood samples in contrast to 8 COVID‐19‐negative blood samples for multiplexed serosurveillance of IgG‐RBD and IgM‐RBD antibodies (Figures S30–S32, Supporting Information).

**Figure 5:**
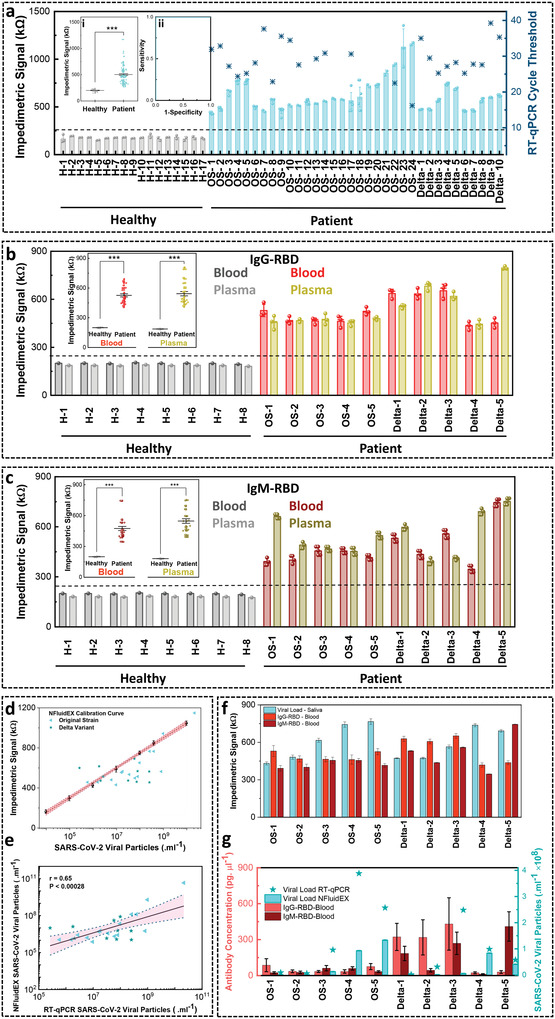
Assessment of quantitative multiplexed NFluidEX for the detection of SARS‐CoV‐2 in saliva and blood samples using COVID‐19‐positive and ‐negative subjects. (a) The impedimetric signal analysis of saliva samples comparing healthy and patient signals, with inset (i) null comparison demonstrating the distinguished signal level in patients with COVID‐19‐positive compared to healthy controls (*F*_1122_ = 161.77, *p* = 4.11E‐24), and inset (ii) the ROC curve showing 100% sensing efficiency. (b) The serosurveillance of IgG‐RBD from COVID‐19‐positive and ‐negative subjects with the inset showing statistical analysis of the impedimetric signal average level in patient whole blood (*F*_1,27_ = 261.78, *p* = 2.03E‐15) and plasma samples (*F*_1,27_ = 105.05, *p* = 8.34E‐11) compared to healthy controls, *** *p* < .001. (c) The serosurveillance of IgM‐RBD from COVID‐19‐positive and ‐negative subjects with an inset statistical analysis of the impedimetric signal average level in patient whole blood (*F*_1,36_ = 48.61, *p* = 3.57E‐8) and plasma samples (*F*_1,36_ = 73.19, *p* = 3.37E‐10) compared to healthy controls, *** *p* < .001. (d) Quantitative correlation of NFluidEX impedimetric signal from COVID‐19‐positive samples with the test assay calibration curve based on viral particle load. (e) The linear regression and 95% confidence intervals to compare the statistical significance of the NFluidEX quantitative response with RT‐qPCR. (f) Case study of NFluidEX response for 10 patients using both the diagnosis and serology tests. (g) Quantitative response of the NFluidEX case study compared with RT‐qPCR. Data shows mean values ± standard deviation (n = 3).

We analyzed a set of randomized samples belonging to the SARS‐CoV‐2 original strain and the Delta B.1.617.2 variant. In order to quantifiably assess the impedimetric signal of NFluidEX towards SARS‐CoV‐2 viral concentration, the signals from a cohort of healthy samples (*n* = 17) were compared to the signal from a cohort of patient samples (*n* = 34) clinically diagnosed with SARS‐CoV‐2 original strain and Delta variant (**Figure** **5**a). A clear threshold to identify the SARS‐CoV‐2 viral infection based on the impedimetric signal in the patient samples was defined at 250 kΩ regardless of the viral infection strain. A statistically significant difference (*p* < .001) is achieved to differentiate between COVID‐19‐positive patient samples and healthy saliva samples (Figure 5a, inset (i)), demonstrating a 100% sensing efficacy indicated via a receiver operating characteristic (ROC) curve (Figure 5a, inset (ii)). We also tested COVID‐19 positive blood and plasma samples (*n* = 10) of patients with NFluidEX and compared them to healthy samples (*n* = 8). A threshold impedimetric level clearly differentiates between positive patient signals and healthy signals both in blood and plasma for IgG‐RBD and IgM‐RBD antibodies (Figure 5b,c). The post hoc comparisons via Holm–Sidak's test demonstrated a statistically significant difference (*p* < 0.001) between the impedimetric signal of the positive patient samples versus negative patient samples both for blood and plasma, for IgG‐RBD and IgM‐RBD antibodies (Figure 5b,c, inset). **Table** **1** summarizes the performance of the multiplexed NFluidEX test assay in comparison with the results reported by RT‐qPCR and ELISA, where the overall parallel tests demonstrate 100% sensitivity and 100% specificity (Experimental Section, Equations (1)–(4)).

**Table 1:** Summary of NFluidEX performance against current gold standard testing methods

		RT‐qPCR		
		+	–	Total
NFluidEX	+	34	0	34
	–	0	17	17
	Overall result of NFluidEX	34	17	51

			ELISA				
NFluidEX			IgG	IgM	IgG	IgM	Total
			+	+	–	–	
	IgG	+	10	0	1	0	11
	IgM	+	0	10	0	1	11
	IgG	–	0	0	7	0	7
	IgM	–	0	0	0	7	7
	Overall result of NFluidEX	+	10		0		
		–	0		8		

+: positive test result, –: negative test result; Note: Although a single false positive was recorded for IgG and IgM, the combined parallel sensitivity and specificity evaluated at two unique test sites yielded 100% accordance with gold standard methods.

To assess the quantitative nature of the NFluidEX compared to RT‐qPCR, the viral load distribution in the patient samples was calculated based on both methods. For the gold standard RT‐qPCR method, cycle threshold (Ct) values were obtained for all patient samples regardless of their viral strain, according to the established inversely proportional scaling between Ct values and viral loads (Figure S33, Supporting Information). When we compared the estimated viral load distribution of the patient samples from RT‐qPCR with the NFluidEX impedimetric calibration curve (Figure 5d), a significant correlation between the NFluidEX device and conventional RT‐qPCR analysis, with a wide range of Ct values from 16.19 (OS‐24) to 39.24 (Delta‐9) was observed. Interestingly, there are numerous patient samples with low Ct values of between 22 and 26 (OS‐22, OS‐8, OS‐4, OS‐5, Delta‐3, Delta‐6), which were successfully detected by NFluidEX.

The predicted viral content was strongly correlated in the 34 saliva samples that tested positive by both the NFluidEX device and conventional RT‐qPCR analysis (Figure 5e), exhibiting similar mean values (1.87 × 10^9^ and 0.96 × 10^9^ number of viral particles mL^−1^), with significant correlation. Discrepancies between our sensor and the RT‐qPCR results can be attributed to the 30–40% miss rate of RT‐qPCR due to possible poor sample extraction and processing, in addition to challenges in RT‐qPCR sensitivity when amplifying the RdRp, ORF1 ab, and N genes.^[^ ^52^, ^53^ ^]^ However, the quantitative NFluidEX test with a low rate of error defines its potential as a reliable testing method compared to the current gold standard methods. Similarly, the ELISA‐positive patient samples with the ELISA optical density (OD) values ranged from as low as 0.001 (Delta‐4) to 11.19 (OS‐1) were all successfully recognized by the NFluidEX. This further validates the quantitative potential of our platform, while demonstrating a wide range of Ct and OD values for the validation of NFluidEX (Tables S20 and S21, Supporting Information).

As a proof‐of‐concept to demonstrate the potential of the NFluidEX in the synchronous usage of saliva‐based diagnosis and blood‐based serology testing, we conducted a field study for a cohort of 10 patients (*n* = 5 patients clinically diagnosed with the SARS‐CoV‐2 original strain and *n* = 5 patients clinically diagnosed with the SARS‐CoV‐2 Delta B.1.617.2 variant). The dual detection device allowed for an enhanced combined sensitivity over diverse disease manifestations due to higher positive rates of diagnostic tests during the acute phase of infection and high positive rates of serology biomarkers during the convalescent phase of infection.^[^ ^17^, ^18^, ^54^ ^]^ All the patients in this cohort were evaluated 1 week after symptom onset. Regardless of their viral strain, all patients demonstrated positive results for both the NFluidEX saliva‐diagnostic and blood‐serosurveillance tests, which were in accordance with their reported RT‐qPCR and ELISA results (Figure 5f). Quantitative multiplexed signal of NFluidEX monitored the diverse response of the individual patient samples with respect to the viral load and antibody concentration (Figure 5g). It demonstrated that patients in the acute phase of infection displayed higher viral loads and low antibody production (OS‐4, OS‐5, Delta‐4), while patients in the convalescent phase of infection displayed higher antibody levels and lower viral loads (Delta‐1, Delta‐2, Delta‐3). Some patients displayed higher relative IgM‐RBD content (Delta‐5), which is indicative of developing immunity against the viral antigens, while others were still likely in an early‐stages of infection (OS‐1, OS‐2, OS‐3). This study confirmed the potential benefits of quantitative monitoring outcome of heterogeneous disease dynamics that vary on the individual level.^[^ ^55^ ^]^

## 3 Conclusion

Here, we described the development of the NFluidEX device, a portable low‐cost quantitative multiplexed test, analogous to the glucometer, that is capable of parallel quantification of viral load in saliva, and the IgG and IgM antibodies in blood within 11 min. The NFluidEX test assay is designed based on imprinting the geometric structure of viral proteins and antibodies to form binding sites in a molecularly imprinted polymer layer on the surface of a sensing electrode, which offers a versatile response specific to the target (here, Influenza A H1N1 and SARS‐CoV‐2 and its different variants: Alpha B.1.1.7, Delta B.1.617.2, and Omicron B.1.1.529), with minimal variations; the versatility and rapid adaptability of the design enabled a similarly robust detection of various viral entities. The platform functioned with three simple user‐friendly steps, attributed to the combined built‐in sample collection/preparation, on‐chip microfluidic quantitative data acquisition, and a smartphone‐enabled result interpretation/visualization of the risk assessment for untrained users. It is capable of reliably quantifying viral loads with high correlation to current testing methods such as RT‐qPCR (*p* < 0.001, 95% confidence interval). Being an indicator of the stage of infection, IgG and IgM are both the target of the NFluidEX serosurveillance to quantifiably indicate the relative temporal prevalence of the patient's immune response. Overall, the quantitative output from untreated saliva diagnosis coupled with antibody detection provides a complete profile of the patient's diagnosis and risk assessment that accounts for diverse disease manifestations and increases the confidence in the test results. While several new testing platforms have been reported in the context of COVID‐19 pandemic with the aim of translation to point‐of‐care settings,^[^ ^52^, ^56^, ^57^, ^58^, ^59^, ^60^ ^]^ this is the first platform with a fully automated instrument‐free user‐friendly interface that enables parallel monitoring of various viral infections and immune responses in minute scale. This level of confidence helps with decision‐making on the spread management of viral respiratory infections.

## 4 Experimental Section

### Materials

Ortho‐phenylenediamine (o‐PD; Thermo‐Fischer), gold (III) chloride trihydrate (Sigma‐Aldrich). Heat‐inactivated SARS‐CoV‐2 (ATCC VR‐1986HK; Cedarlane), heat‐inactivated Influenza A (0810248CFHI; Cedarlane), human coronavirus spike protein (RDC3141; Cedarlane), MERS‐CoV spike protein (40069‐V08B1; SinoBiological), recombinant SARS‐CoV spike protein (10683‐CV; Novus Biological), anti‐H1N1 Influenza A virus nucleocapsid protein antibody (ab104870; Abcam), anti‐influenza (Ab01106‐15.0 & Ab01103‐15.0), and anti‐MERS‐CoV spike protein antibody (Ab01675‐21.0) (Absolute Antibody), MERS‐CoV/NCov spike protein antibody (NBP3‐06391) and Influenza A Haemagglutinin H1N1 antibody (NBP3‐06578) (Novus Biological), human coronavirus antibody (40640‐T62), and MERS‐CoV nucleocapsid protein antibody (100213‐RP02) (SinoBiological). Healthy human pooled saliva (IRHUSL50ML), single donor human plasma (blood‐derived) (IPLASK2E50ML) and single donor human whole blood (IWB1K2E10ML) samples were bought from Innovative Research to be untreated and stored upon arrival in −80, −20, and 4 °C, respectively. TaqMan Fast Virus 1‐Step Master Mix (4 444 432), and the nCoV_IP2‐12669Fw (5’‐ATGAGCTTAGTCCTGTTG‐3’), the nCoV_IP2‐12759Rv (5’‐CTCCCTTTGTTGTGTTGT‐3’), and the nCoV_IP2‐12696bProbe(+) (5’‐AGATGTCTTGTGCTGCCGGTA‐3’) primers were purchased from ThermoFisher Scientific. Sodium acetate (ASC), acetic acid, and phosphate buffer saline (PBS) 10X were bought in the chemical store of the Université du Quebec à Montréal. The chemicals purchased were analytical grade and were used without further purification. All the solutions were prepared using ultrapure water (>18 MΩ cm) from a Millipore Milli‐Q water purification system. All proteins used for templating the polymer assay are listed in **Table** **2**.

**Table 2:** Summary of Imprinting Template Proteins

Protein Name	Imprinted Protein	Sensing Experiment	Vendor
Human CellExp SARS‐CoV‐2 Spike Protein (RBD), Recombinant (P1530‐10)	SARS‐CoV‐2 SP Original Strain	Sensitivity of SARS‐CoV‐2 SP and heat‐inactivated SARS‐CoV‐2, OS patient samples	Cedarlane
Spike S1 (B.1.1.7 Variant), Avi‐His‐Tag (SARS‐CoV‐2) (101001‐1)	SARS‐CoV‐2 SP Alpha variant	Sensitivity of SARS‐CoV‐2 SP Alpha variant	Cedarlane
Recombinant SARS‐CoV‐2 B.1.617.2 Spike RBD His Protein, CF (10876‐CV‐100)	SARS‐CoV‐2 SP Delta variant	Sensitivity of SARS‐CoV‐2 SP Delta variant, Delta patient samples	Cedarlane
SARS‐CoV‐2 B.1.1.529 (Omicron) Spike S1 Protein (His Tag) (40591‐V08H41)	SARS‐CoV‐2 SP Omicron variant	Sensitivity of SARS‐CoV‐2 SP Omicron variant	SinoBiological
SARS‐CoV‐2 Spike Antibody (CR3022) (NBP2‐90980)	SARS‐CoV‐2 IgG‐RBD	Sensitivity of SARS‐CoV‐2 IgG‐RBD, OS and Delta patient samples	Cedarlane
SARS‐CoV‐2 Spike RBD Antibody (NBP3‐07033)	SARS‐CoV‐2 IgM‐RBD	Sensitivity of SARS‐CoV‐2 IgM‐RBD, OS and Delta patient samples	Novus
SARS Nucleocapsid Protein Antibody (NBP3‐05721)	SARS‐CoV‐2 IgG‐N	Sensitivity of SARS‐CoV‐2 IgG‐N, OS and Delta patient samples	Cedarlane
Anti‐Covid‐19 & SARS‐CoV Nucleocapsid (Ab02325‐15.0)	SARS‐CoV‐2 IgM‐N	Sensitivity of SARS‐CoV‐2 IgM‐N, OS and Delta patient samples	Absolute Antibody
Recombinant (HEK) Influenza A H1N1 HA protein (NBP1‐99041)	Influenza SP	Sensitivity of Influenza SP and heat‐inactivated Influenza viral particles	Cedarlane

### Solution Preparations

The gold solution for NMIs electrodeposition was prepared from gold (III) chloride trihydrate (HAuCl_4_.3H_2_O) in 0.5 m hydrochloric acid (HCl).^[^ ^61^, ^62^ ^]^ The 10 mm electro‐monomer o‐PD solution was prepared in acetate buffer. A washing solution was prepared with 0.1 M sodium hydroxide (NaOH) dissolved in ethanol and water at ratio of 5:1. The study of SARS‐CoV‐2 spike proteins was done in PBS (pH 7.2) and real human saliva from healthy patient samples. Solutions were spiked with 10–10^5^ pg mL^−1^ SARS‐CoV‐2 spike protein to fit in a biologically relevant range; a similar study was performed for the spike protein of the Alpha B.1.1.7, Delta B.1.617.2, and Omicron B.1.1.529 variants, and the Influenza hemagglutinin protein. SARS‐CoV‐2 and Influenza A heat‐inactivated virus was used to spike PBS (pH 7.2) and real human saliva over a range of 1 × 10^2^–4 × 10^6^ PFU mL^−1^; equivalent viral particle copies were provided by the company's certificate of analysis (CoA), where not, the conversions were taken from the CoA to approximate the number of viral particles in solution.

In this case, the supplier provided the Median Tissue Culture Infectious Dose (TCID_50_) per millilitre of the viral titer and performed a digital polymerase chain reaction (dPCR) analysis on the sample as a quality control measure to quantify the nucleic acid content in terms of genome copies per microliter. Assuming a single genome copy per viral particle,^[^ ^55^ ^]^ a quantitative measure for the number of viral particles per volume of the sample was performed. For selectivity studies, spike proteins from similar viruses including SARS‐CoV‐1, HCoV‐229E, MERS‐CoV, and Influenza A H1N1 were incubated on the surface assay at concentrations of 10^4^, 10^3^, and 10 pg mL^–1^. The study of SARS‐CoV‐2 antibodies (IgG‐RBD, IgM‐RBD, IgG‐N and IgM‐N) was conducted using PBS (pH 7.2), plasma, and whole blood samples from healthy patients. These biofluids were spiked over biologically relevant concentration ranges from of the solutions from 10 to 10^4^ pg µL^–1^. For the selectivity tests, IgG and IgM antibodies against the spike and nucleocapsid of similar viruses like HCoV‐229E, MERS‐CoV, and Influenza A H1N1 were incubated on the assay surface over 10^2^ and 50 pg µL^–1^.

### Microfluidic Device, Integrated Electrodes, NMIs, and Biomimic MIPs

Fabrication details are in Figure S6, Supporting Information. Briefly, the microfluidic device was based on an indium tin oxide (ITO)‐coated 4 in. glass wafer. Chemical vapour deposition was used for the deposition of 5–10 µm insulating silicone dioxide, followed by the sequential electron‐beam deposition of oxide, an attachment layer, and gold at a ratio of 6:1:10, then patterned by lift‐off process to fabricate the reference electrode (RE) and counter electrode (CE) connections. The width of each electrode and the connection dimensions mimic standard SPE for ease of connection with a portable potentiostat via an SPE adapter (IO Rodeo, Smart Lab Technology). An SU‐8 mold was used to construct microfluidic channels using SU‐8 2050 photoresist patterned through standard lithography followed by cutting of the wafer into individual chips (length: 57 mm, width: 24 mm). This was followed by a three‐electrode electrodeposition method of gold to generate 3D hierarchical NMIs at the analysis wells base. The electrodeposition was performed through an Autolab potentiostat/galvanostat (model: PGSTAT204), with an Ag/AgCl reference electrode and a platinum wire counter electrode. The supporting electrolyte solution for the 3D gold NMIs was HAuCl_4_ (1 mm) in HCl (0.5 m). Synthesis was carried out at the applied fix potential of 600 mV versus Ag/AgCl.^11^ Electrosynthesis of the polymer was carried out following a facile electropolymerization method. Briefly, a solution o‐PD was prepared using sodium acetate and H_2_SO_4_ solutions. SARS‐CoV‐2 protein and antibody were added to the solution from a stock concentration and the volume of the monomer solution to the SP/antibody solution was maintained at a ratio of 95:5. Further, electropolymerization of o‐PD polymer on NMIs electrode was carried out using cyclic voltammetry (CV) technique at a scan rate of 50 mV s^−1^. Eventually, the samples were washed with ethanol and water solution containing 0.1 m NaOH to remove the template. Similarly, a control assay modified by NIP was fabricated without using SARS‐CoV‐2 protein or antibody as the template. The detailed optimization studies on MIP layer generation for SARS‐CoV‐2 protein/antibody, including the optimal number of cycles needed for electropolymerization for o‐PD are discussed in the Supporting Information. Polydimethylsiloxane (PDMS) was prepared at a 10:1 ratio of elastomer to crosslinker and degassed in a desiccator before coating the wafer. This was cured overnight at 105 °C,^[^ ^63^ ^]^ followed by the selective removal of PDMS to expose the electrode connections and to open the inlet/outlet of the fluidic channels. Additive manufacturing using a polylactic acid (PLA) thermoplastic polymer filament and Formlabs resin on a 3D printer (Original Prusa i3 MK3S+ and Form 3, Formlabs) was used to fabricate the sample collection kit and the signal transduction housing unit. All designs were made on AutoCAD 2022 and Fusion 360 (Autodesk) and printed using standard fused filament fabrication (FFF) and stereolithography (SLA) methodologies. Electrochemical impedance spectroscopy (EIS) measurements were carried out using a custom‐built 4‐layer, 89 × 74 mm printed circuit board (PCB) base potentiostat device inspired by an open‐source design.^[^ ^64^ ^]^ In the current design, the RN‐42 Bluetooth module (Microchip, Chandler AZ) was replaced with a CYBLE‐014008‐00 Bluetooth module (Cypress, San Jose CA) with an updated protocol version, and the ESP‐12S microcontroller (Espressif Systems, Shanghai, China) was replaced with the ESP‐12F model to allow for extra pin connections. A 1‐channel relay module was connected to this microcontroller to allow for quasisimultaneous EIS measurements between the IgG and IgM testing assays. The spring‐loaded electrode terminals were also replaced with SPE adaptors to allow for easy connection of the microfluidic test chip. The connected SPE adaptors were fitted into a side port accordingly to each align with one of the microfluidic chip's exposed‐electrode test areas (IgG‐RBD, IgM‐RBD, and whole virus via SP).

### Assay Incubation Time

The surface of the sensor was incubated with 10–10^5^ pg mL^−1^ of SARS‐CoV‐2 SP in both 6.7 mm PBS (pH 7.2) solution and human saliva. Similarly, the sensor was incubated with 10–10^4^ pg µL^−1^ of SARS‐CoV‐2 IgG‐RBD and IgM‐RBD in both 6.7 mm PBS (pH 7.2) solution and undiluted plasma for various times intervals from 5 to 40 min. The selection of the appropriate incubation time was determined by studying the interface properties of the sensor surface via electrochemical impedance spectroscopy.

### Simulation‐Based Studies

For the MD studies, AutoDock (version 4.2.6, Scripps Research) was used in addition to the genetic algorithm to determine the preferential molecular conformations in each of the selected boxes, denoted by the coordinates in Tables S2 and S3 (Supporting Information). For this study, each box was evaluated for 150 runs and the optimal free energy of binding was recorded for each structure. The monomer structure of o‐PD was designed using HyperChem (Hypercube Inc.) for molecular modeling of chemical structures. The Parametric Method 3 (PM3) semiempirical method was used to perform optimization on the geometry of the proposed o‐PD structure, but since the o‐PD monomer was relatively simple, its conformation remained the same before and after PM3 optimization. The simulations were done using the 6VXX Protein Data Bank (PDB) file for the SP and the antigen binding fragment portion of the 7BWJ PDB file for the RBD antibodies.

### Affinity Studies

A series of SPR experiments were performed to determine the *K*_d_ of the different targets to the NMI/MIP and NMI/NIP assays. Specific nanoimprinted assays were fabricated for the SARS‐CoV‐2 viral entities, including the SP, IgG‐RBD, and IgM‐RBD. Each assay was uniquely fabricated on three different gold sensors specific for the P4SPR portable SPR equipment. Briefly, the sensor is, as provided, a prism with a face deposited with a thin gold film. The electrode is then treated overnight with16‐Mercaptohexadecanoic acid and dried with N_2_. Next the NMI were fabricated on the sensing area via electrodeposition and the MIP or NIP were electropolymerized following the optimized protocols presented in this manuscript. After, the sensor was loaded and secured into the Affinité Instruments SPR reader. The assay was sequentially loaded with target protein from low to incrementally higher concentrations. The lowest concentrations on the established linear range were prepared in 1X PBS buffer and incubated on assay for 10 min, followed by a stable reading after 10 min to minimize any noise and baseline drift. After the data was recorded, the next solution was added to achieve the desired concentration for the accumulation study until the linear range was covered. All experiments were performed with ≈500 µL of sample and the change in wavelength was recorded for each concentration point. Subsequently, the nonlinear Hill function was used to fit the data in OriginLab 2022.

### Sensing Performance

EIS in the presence of buffer (PBS, pH 7.2), saliva, plasma, and blood as well as clinical samples was carried out using a potentiostat/galvanostat (Ivium, model: AUT84915). The tests were performed after 1 min incubation of human blood and 10 min incubation of human plasma and saliva on the surface of the test assays. The signals were recorded at open circuit potential, in the range of 100 kHz to 100 mHz, and with a potential amplitude of 10 mV. The EIS tests were repeated at least three times, simulated with Zview software (version 3.1), and the average results with the standard deviation were reported. All sensing measurements were taken in a 1 mm K_3_Fe(CN)_6_ solution as the redox molecule. The analytical performance of the assay was evaluated by calculating the sensitivity (Equation (1)) and specificity (Equation (2)) as the fraction of true positives (TP) and the fraction of true negatives (TN) in patients, respectively. These are measures to evaluate the ability of the assay to rule out cases of false positives (FP) and false negatives (FN). For two tests performed in parallel (diagnostic and serology), the combined parallel sensitivity (Equation (3)) and specificity (Equation (4)) of the device should be considered

(1) $$\mathrm{Sensitivity}=\frac{\mathrm{TN}}{\mathrm{TN}+\mathrm{FP}}\times 100\%$$

(2) $$\mathrm{Specificity}=\frac{\mathrm{TP}}{\mathrm{TP}+\mathrm{FN}}\times 100\%$$

(3) $$\begin{array}{c} \begin{array}{c} \mathrm{Parallel}\;\mathrm{Sensitivity}=\left(1-\left(1-\frac{{\mathrm{Sensitivity}}_{1}}{100}\right)\times \left(1-\frac{{\mathrm{Sensitivity}}_{2}}{100}\right)\right)\times 100\% \end{array} \end{array}$$

(4) $$\mathrm{Parallel}\;\mathrm{Specificity}=\left(\frac{{\mathrm{Sensitivity}}_{1}}{100}\times \frac{{\mathrm{Sensitivity}}_{2}}{100}\right)\times 100\%$$

### COVID‐19 Patient Sample Study

Human saliva, blood, and plasma samples were collected from the patients who were admitted at “Erythron Laboratory,” a cooperator laboratory of Isfahan University of Medical Sciences (IR.MUI.MED.REC.1400.066 and McGill IRB Internal Study Number: A03‐M24‐21B). Free authorization and consent forms were signed by patients, and their clinical samples were collected according to the laboratory regulation. 15 saliva samples were collected from adult patients with COVID‐19 symptoms, such as fever, fatigue, and dry cough, and tested with RT‐qPCR (LightCycler 480, Roche) using primers (nCoV_IP2‐12669Fw, nCoV_IP2‐12759Rv, nCoV_IP2‐12696b Probe(+)) targeting the RdRp gene/nCoV_IP2 in the ORF1ab prior to electrochemical studies. 5 samples were determined to belong to the original strain of SARS‐CoV‐2 and 10 samples belonged to the Delta B.1.617.2 variant. In addition, 19 patient saliva samples were collected from the University Health Network's PRESERVE‐Pandemic Response Biobank for testing on the assay (REB #20‐5364). All samples tested positively using RT‐qPCR (QuantStudio 12K Flex, ThermoFisher) and were determined to be from the original strain of SARS‐CoV‐2 prior to electrochemical sensing. The samples were assessed at a Level 2+ facility situated in the Montreal Jewish General Hospital. In addition, 10 patient blood and plasma samples were collected for antibody evaluations, of which, 5 samples belonged to the original strain of SARS‐CoV‐2 and 5 samples belonged to the Delta B.1.617.2 variant. The blood samples were tested with ELISA reader (EUROIMMUN Analyzer I‐2P). The ELISA results were presented as the cut‐off index (COI) value for IgG‐N, targeting nucleocapsid protein of SARS‐CoV‐2, and the sum of IgM‐N and IgM‐S, targeting nucleocapsid and spike proteins of SARS‐CoV‐2, respectively. Accordingly, the samples with a COI of higher than 1.1 and lower than 0.9 were considered as positive and negative samples, respectively. The optical density was recorded at 450 nm using 3,3,“5,5”‐Tetramethylbenzidine and acidic stop solution.

### Statistical Analysis

Results were presented as the mean ± the standard deviation over triplicate readings. Statistical analysis was performed using OriginPro (OriginLab, 2021). The limits of detection and linear ranges were computed using linear regression parameters including the line slope and the standard error of the intercept. Statistical significance was calculated by performing a one‐wayANOVA with post hoc Holm–Sidak's test for mean comparison. The differences between datasets were considered statistically significant for *p* < 0.05. The figures were generated using the Paired Comparison Plot (version 3.60, OriginLab) graphing application using conservative *p* values.

## Conflict of Interest

The authors declare no conflict of interest.

## Author Contributions

S.M. and R.S.M. contributed to the idea conception. R.S.M. and C.d.R.M. contributed to the design, planning, and execution of the investigation. J.d.V., J.S., and H.S. contributed to design and execution of the automated device. R.S.M., M.J., and H.S. contributed to data analysis. C.d.R.M., H.S., Y.L., and R.S.M. contributed to the fabrication of the test assay. I.I.H., S.G.Y., and T.A. contributed to the fabrication of the microfluidic device. I.I.H. and H.S. contributed to a theoretical study using FDTD simulations, and F.G.H. contributed to molecular docking simulations. M.A.T. and C.L. contributed to the acquisition of patient samples and revision of the manuscript. A.S. contributed to performing electrochemical field tests, T.A. and M.M. contributed to performing electrochemical tests at the Montreal Jewish General Hospital. R.S.M., M.J., H.S., Y.L., and S.S.M. contributed to the preparation of the manuscript, with the support and collaboration of all coauthors. S.M. and S.S.M. contributed to the project conception, design of the experiments, interpretation of results. S.M. contributed to resources and funding acquisition, and supervision of the work. S.S.M., M.A.T., and C.L. are senior authors. S.M. is the corresponding author.

## Supporting information

Supporting Information

Supplemental Movie 1

Supplemental Movie 2

## Data Availability

The data that support the findings of this study are available from the corresponding author upon reasonable request.;
