# Supplementary material for: A Versatile Biomimic Nanotemplating Fluidic Assay for Multiplex Quantitative Monitoring of Viral Respiratory Infections and Immune Responses in Saliva and Blood
Source: Adv Sci (Weinh). 2022 Oct 17;9(33):2204246. doi: 10.1002/advs.202204246 (PMC9685479; doi:10.1002/advs.202204246)
Supplement: Supplementary file 1 — Supporting Information [file ADVS-9-2204246-s003.pdf]

ADVANCED SCIENCE Open Access

Supporting Information

for *Adv. Sci.*, DOI 10.1002/advs.202204246

A Versatile Biomimic Nanotemplating Fluidic Assay for Multiplex Quantitative Monitoring of Viral Respiratory Infections and Immune Responses in Saliva and Blood

*Roozbeh Siavash Moakhar, Carolina del Real Mata, Mahsa Jalali, Houda Shafique, Alireza Sanati, Justin de Vries, Julia Strauss, Tamer AbdElFatah, Fahimeh Ghasemi, Myles McLean, Imman I. Hosseini, Yao Lu, Sripadh Guptha Yedire, Sahar Sadat Mahshid, Mohammad Amin Tabatabaiefar, Chen Liang and Sara Mahshid**

Supporting Information

A Versatile Biomimic Nano-Templating Fluidic Assay for Multiplex Quantitative Monitoring of Viral Respiratory Infections and Immune Responses in Saliva and Blood

*Roozbeh Siavash Moakhar, Carolina del Real Mata, Mahsa Jalali, Houda Shafique, Alireza Sanati, Justin de Vries, Julia Strauss, Tamer AbdElFatah, Fahimeh Ghasemi, Myles McLean, Imman I. Hosseini, Yao Lu, Sripadh Guptha Yedire, Sahar Sadat Mahshid, Mohammad Amin Tabatabaiefar, Chen Liang, Sara Mahshid**

Roozbeh Siavash Moakhar, Carolina del Real Mata, Mahsa Jalali, Houda Shafique, Justin de Vries, Julia Strauss, Tamer AbdElFatah, Imman I. Hosseini, Yao Lu, Sripadh Guptha Yedire, Sara Mahshid
McGill University, Department of Bioengineering, Montreal, Quebec, Canada.
E-mail: sara.mahshid@mcgill.ca

Alireza Sanati
Biosensor Research Center, School of Advanced Technologies in Medicine, Isfahan University of Medical Sciences, Isfahan, Iran.

Fahmieh Ghasemi
Department of Bioinformatics and Systems Biology, School of Advanced Technologies Medicine, Isfahan University of Medical Sciences, Isfahan, Iran.

Myles McLean, Chen Liang
Department of Medicine, McGill University, Montreal, Quebec, Canada.
Lady Davis Institute for Medical Research and McGill AIDS Centre, Jewish General Hospital, Montreal, Quebec, Canada.

Sahar Sadat Mahshid
Biological Sciences, Sunnybrook Research Institute, Sunnybrook Health Sciences Centre, Toronto, Ontario, Canada.

Mohammad Amin Tabatabaiefar
Department of Genetics and Molecular Biology, School of Medicine, Isfahan University of Medical Sciences, Isfahan, Iran.

Keywords: viral respiratory infection, molecularly imprinted polymer, microfluidic devices, impedimetric biosensor, multiplexed testing

1. Fabrication and characterization of the NFluidEX

1.1 Signal transduction module

Portable electrochemical workstation

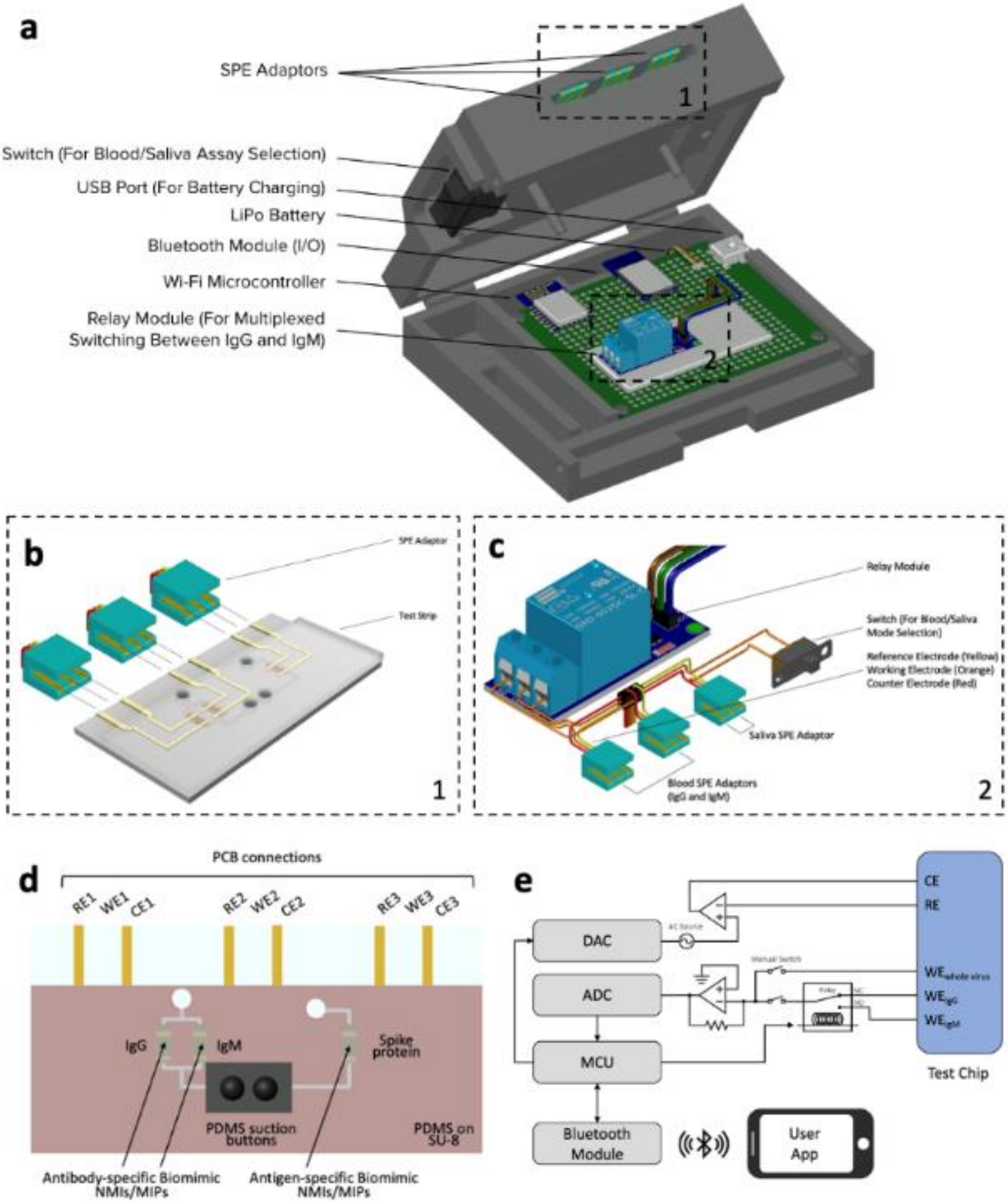

Figure S1. Schematic representation of the interior assembly of the electrochemical device for portable point-of-care testing. The NFluidEX potentiostat design included an updated Bluetooth protocol (Bluetooth Low Energy 5.1) and a relay module to allow

multiplexed detection for both serological test assays (IgG and IgM) and diagnostic assay (whole virus via spike protein [SP]). (a) Schematic of open housing unit labelled with primary electrochemical components. (b) Close-up view of alignment between the microfluidic test strip and the SPE adaptors embedded into the side port of the potentiostat housing unit. (c) Close-up view of switch setup for multiplexed analysis. A manual switch allows for user-mediated switching between blood or saliva sample mode. (d) Top view of test strip, detailing its compartments for multiplexed sensing. (e) Block diagram of potentiostat network with circuit schematic of the multiplexed switch setup. Working electrodes of the IgG and IgM testing assays are connected to the NO (Normally Open) and NC (Normally Closed) ports of the relay, which rapidly flips the AC voltage between them to perform pseudo-simultaneous measurements.

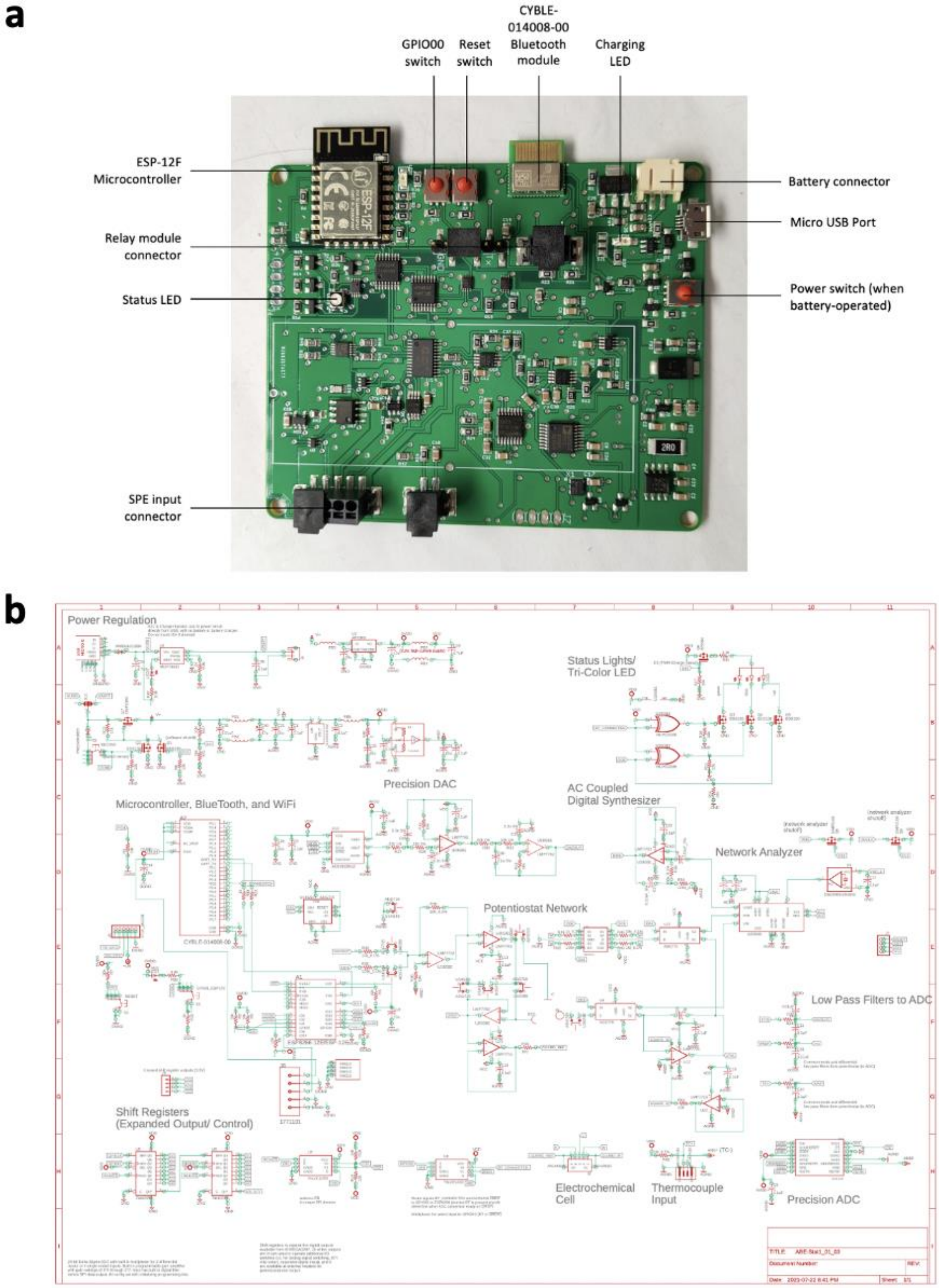

Figure S2. Detailed layout of the printed circuit board modified for NFluidEX testing. (a) Top view of PCB labelled with primary electronic components. (b) Blueprint of PCB detailing the function of each group of electronic components and the connectivity between

them. This blueprint is also included in the attached files and can be viewed in Autodesk EAGLE for closer inspection.

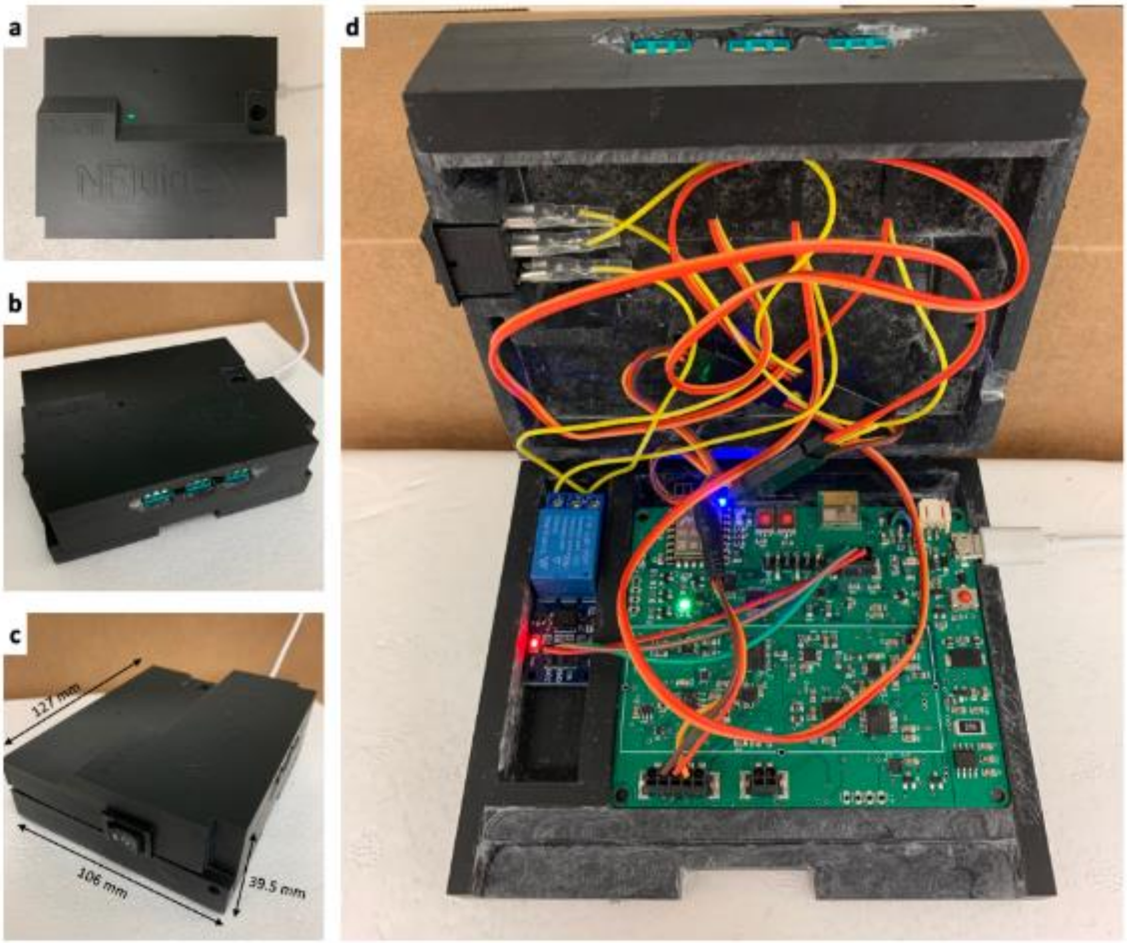

Figure S3. Portable electrochemical device for point-of-care testing of blood and saliva samples. The device is controlled via an ESP-12F WiFi microcontroller (Espressif Systems, Shanghai, China), which receives user-input signals from a CYBLE-014008-00 Bluetooth module (Cypress, San Jose CA, USA) and transmits them to an analog potentiostat network via a digital-to-analog converter (DAC). (a) Top view of closed unit; a green status LED indicates that the potentiostat is ready for measurements. (b) Front view of closed unit showing side port fitted with three SPE adaptors for facile insertion of the NFluidEX test strip. (c) Side view of closed unit showing manual switch for user-mediated selection between diagnostic and serology testing: | = serology test, || = diagnostic test, O = device off. (d) Interior view of unit fully assembled with all device components. For each assay, the current response of the system is recorded, passed through an analog-to-digital converter (ADC), and transmitted back to the Bluetooth module for interfacing to a custom Android smartphone application. The output current measurements are used to calculate the frequency-dependent impedance magnitude on a Bode plot. The entire device can be powered via a standard lithium-polymer battery or via micro-USB, which also recharges the battery when power is

depleted. All the potentiostat components are contained within a 3D-printed housing unit (127 mm x 106 mm x 39.5 mm).

Digitalization and Software

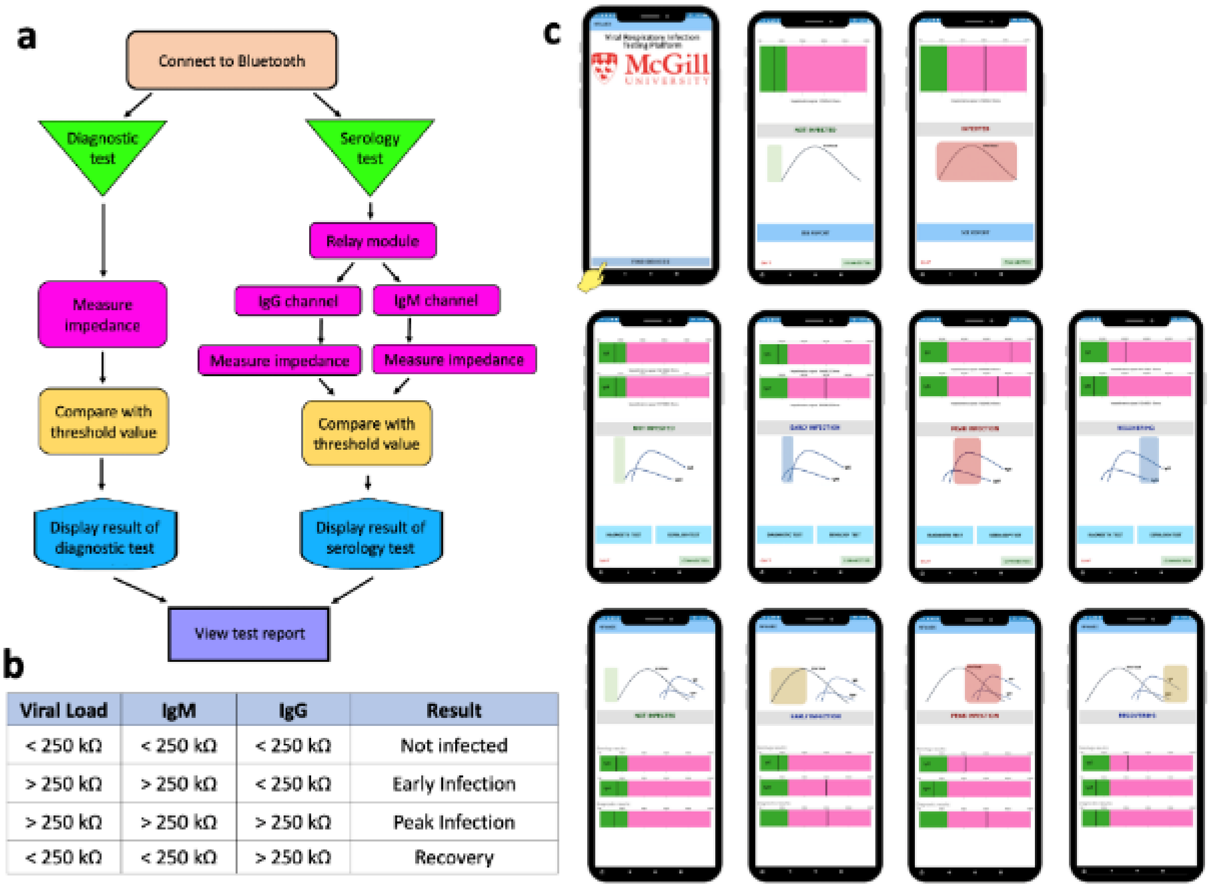

Viral Load	IgM	IgG	Result
< 250 kΩ	< 250 kΩ	< 250 kΩ	Not infected
> 250 kΩ	> 250 kΩ	< 250 kΩ	Early Infection
> 250 kΩ	> 250 kΩ	> 250 kΩ	Peak Infection
< 250 kΩ	< 250 kΩ	> 250 kΩ	Recovery

Figure S4. Digitization of the EIS readout signal. To perform the electrochemical measurements, we designed an EIS smartphone application (based on the open-source Android application provided by Jenkins et al.[1]) that communicated with a Bluetooth Low Energy (BLE) module; set parameters include the sample per frequency decade as 1, DC bias voltage as 0, signal amplitude as 0.01 V, and electrode configuration as ‘3-electrode’. (a) Flow chart of software interface to perform EIS measurements in different test assays. (b) Decision chart with threshold values based on three times the standard deviation of the highest healthy samples. (c) (top) The results of the saliva diagnostic test for whole viral particle presence, (middle) the results of the serological blood test for antibody presence, and (bottom) the results of the overall test with combined diagnostic and serology testing.

1.2 Sample collection cartridge

There is no established protocol at the point-of-care for saliva collection on a microfluidic chip. The primary challenge remains the high viscosity of saliva caused by the presence of mucin glycoproteins, which result in a matrix-like and stringy consistency in saliva that can lead to inaccuracies when pipetting and aliquoting a sample.[2,3] A high viscosity results in high internal friction of the fluid, which impedes its ability to flow in microchannels. We proposed the use of a filter-based technique that can remove large glycoproteins from the saliva while effectively reducing its viscosity with results comparable to that of from the centrifugation[4,5]. This is being done via an integrated self-collection funnel that connects to the microfluidic device using custom 3D-printed attachments.

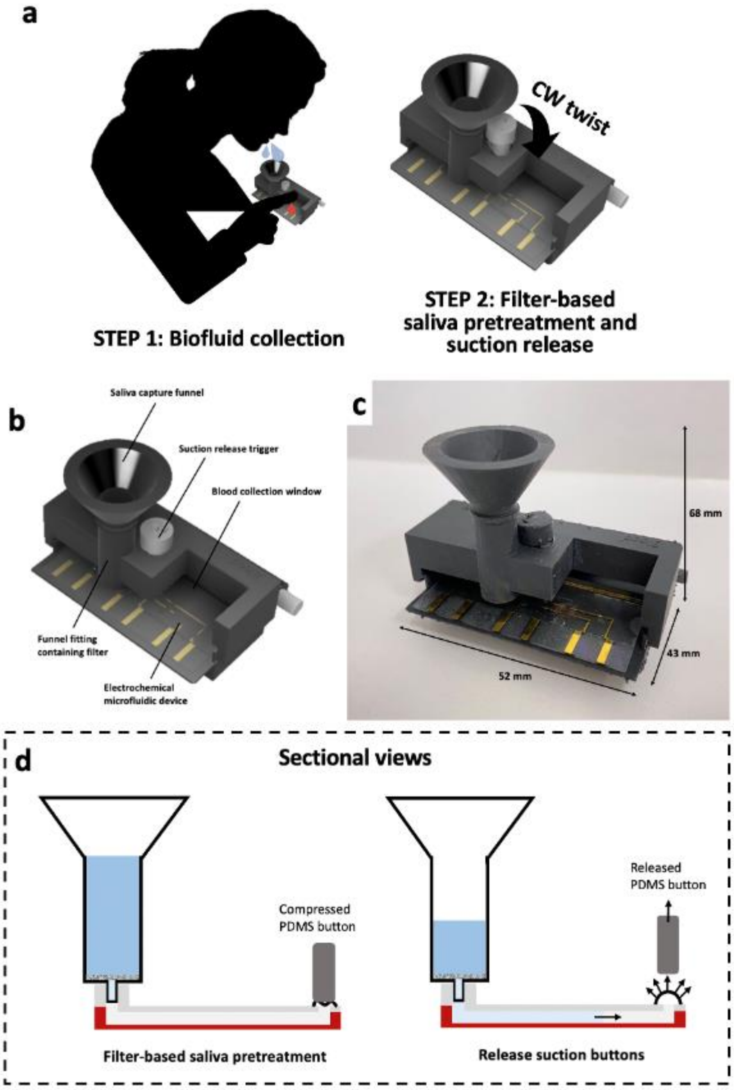

Figure S5. Sample collection cartridge. (a) The design of the removable sample collection kit that combines sample collection, pre-treatment, and microfluidic flow on a single

apparatus, (b) Perspective view of the sample collection cartridge with labelled components; a saliva capture funnel for direct self-collection of saliva, a blood collection window that exposes the inlet of the blood microchannel to the finger prick blood from the user, a single-release trigger that is used to press down on the PDMS soft lithography buttons (only when the trigger is removed, the buttons will be lifted to enable the suction-based flow), (c) real image of 3D-printed cartridge with inserted electrochemical microfluidic device and corresponding dimensions, (d) Sectional view of the sample collection workflow and point-of-care automated biofluid flow.

Fabrication of multiplex fluidic chip embedded with test assay

A two-step aligned standard lithography was used to pattern the electrochemical electrodes on an indium tin oxide (ITO) glass-coated wafer while a single-step lithography was utilized to pattern the fluidic channels. Initially the ITO-coated glass was deposited with a 5-10 µm silicon dioxide insulating layer using plasma-enhanced chemical vapor deposition (PECVD) at a deposition rate of 10 nm. s^{-1}. Then the electrochemical reference electrode (RE) and counter electrode (CE) were patterned in an AZ9245 photoresist followed by etching the patterned electrodes in the SiO_2 via BOE etching (**Figure S6a**). A thin-film consisting of oxide, an attachment layer, and gold at a ratio of 6:1:10 was deposited via electron-beam deposition (BJD 1600) followed by the second lithography step to pattern the electrode in a Shipley photoresist. A wet etching step using HF was utilized to remove the un-patterned gold thin-film and develop well isolated conductive gold electrodes upon photoresist lift-off (**Figure S6b**) with a final RE and CE dimensions corresponding to those of a standard screen-printed electrode to confer compatibility with the adapters assembled in the PCB. Next, a tertiary lithography step was used to fabricate the multiplex fluidic channels in an SU-8 layer with a thickness of ~50 µm aligning the sensing chamber over the electrochemical electrodes (**Figure S6c**). A bottom-up fabrication method based on chronoamperometric growth of gold nanostructures from $HAuCl_4$ solution was used to fabricate the working electrode with gold nano/micro island (NMI) structures in the sensing chamber of the microfluidic device.[6–8] To complete the assay fabrication on the working electrode, a thin-film of o-PD polymer (5-10 nm) was electrodeposited over the NMI surface via cyclic voltammetry approach (**Figure S6d**) followed by the electropolymerization with SARS-CoV-2 SP and antibody binding sites (fabrication and optimization described in **Section S1.4. the NMI/MIP assay**). Finally, a PDMS layer with the same size as the wafer (57 mm x 24 mm) was bonded to the wafer to encapsulate the channels via plasma treatment (**Figure S6e**).

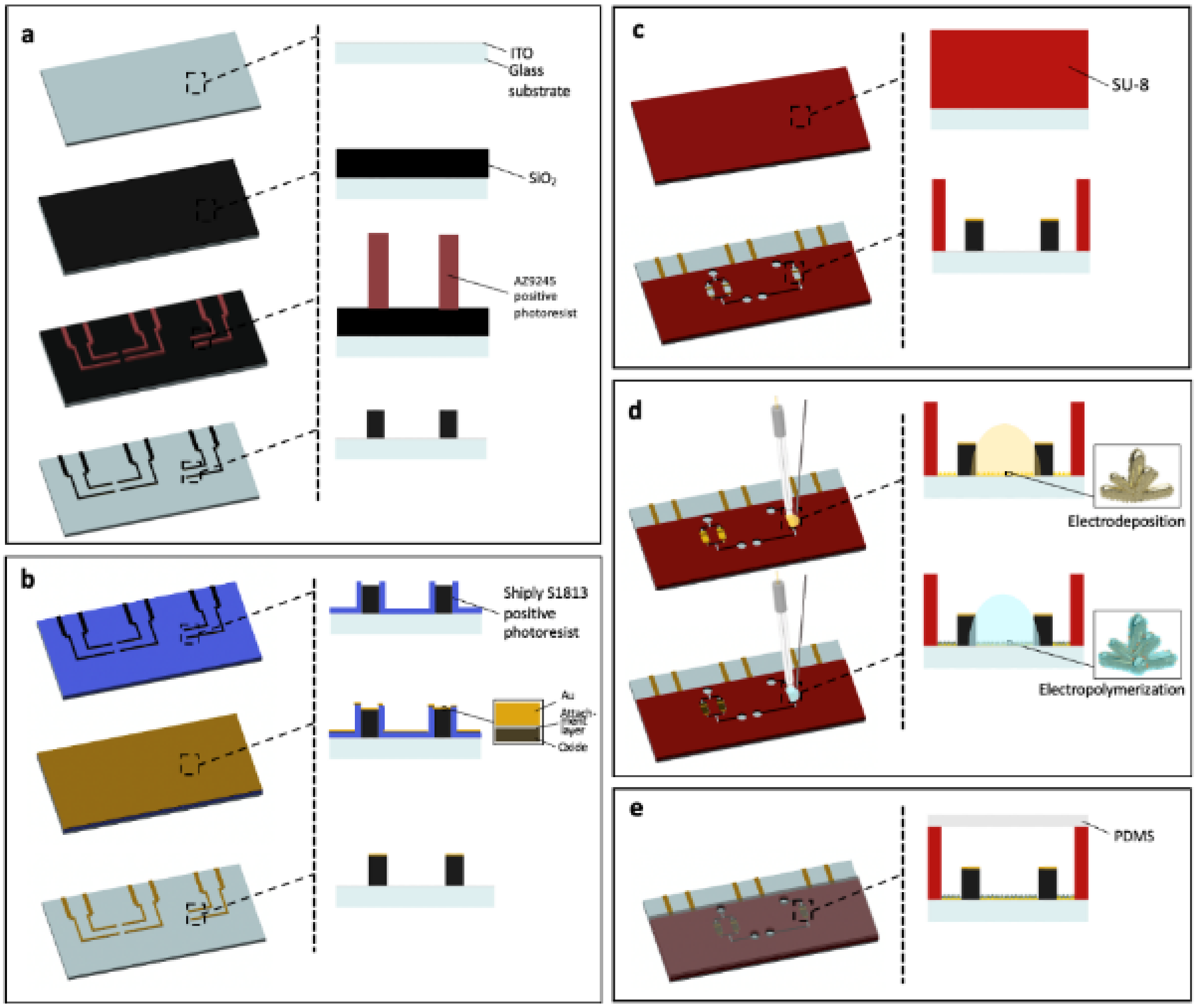

Figure S6. Schematic representation of the microfluidic device fabrication with embedded on-chip electrodes; top-view (left), cross-section-view (right). (a) First step lithography to pattern electrode space in an isolating SiO_2 thin film covering the ITO-coated glass wafer. (b) Sequential electron-beam evaporation of oxide, an attachment layer, and gold thin film and second lithography step to etch the thin film with the electrode pattern to obtain isolated conductive RE and CE electrodes. (c) Third step lithography to pattern the multiplex fluidic pattern in an SU-8 photoresist layer. (d) Bottom-up fabrication of gold NMI structures using electrodeposition followed by electropolymerization of o-PD with SARS-CoV-2 SP and antibody binding sites shown in the inset. (e) PDMS bonding with the fabricated substrate to encapsulate the device with a punched inlet and outlet for fluid flow.

Cross infection caused by mixing of blood and saliva biofluids on the microfluidic device was avoided by constructing microchannels with unique reaction chambers for each impedimetric assay. Separated fluidic chambers allow for the multiplexed treatment of unique biofluids with controlled fluidic manipulation in individual chambers (**Figure S7, Movie S1**), resulting in spatially distinct readouts that have provided 100% sensitivity and 100% selectivity for our system.

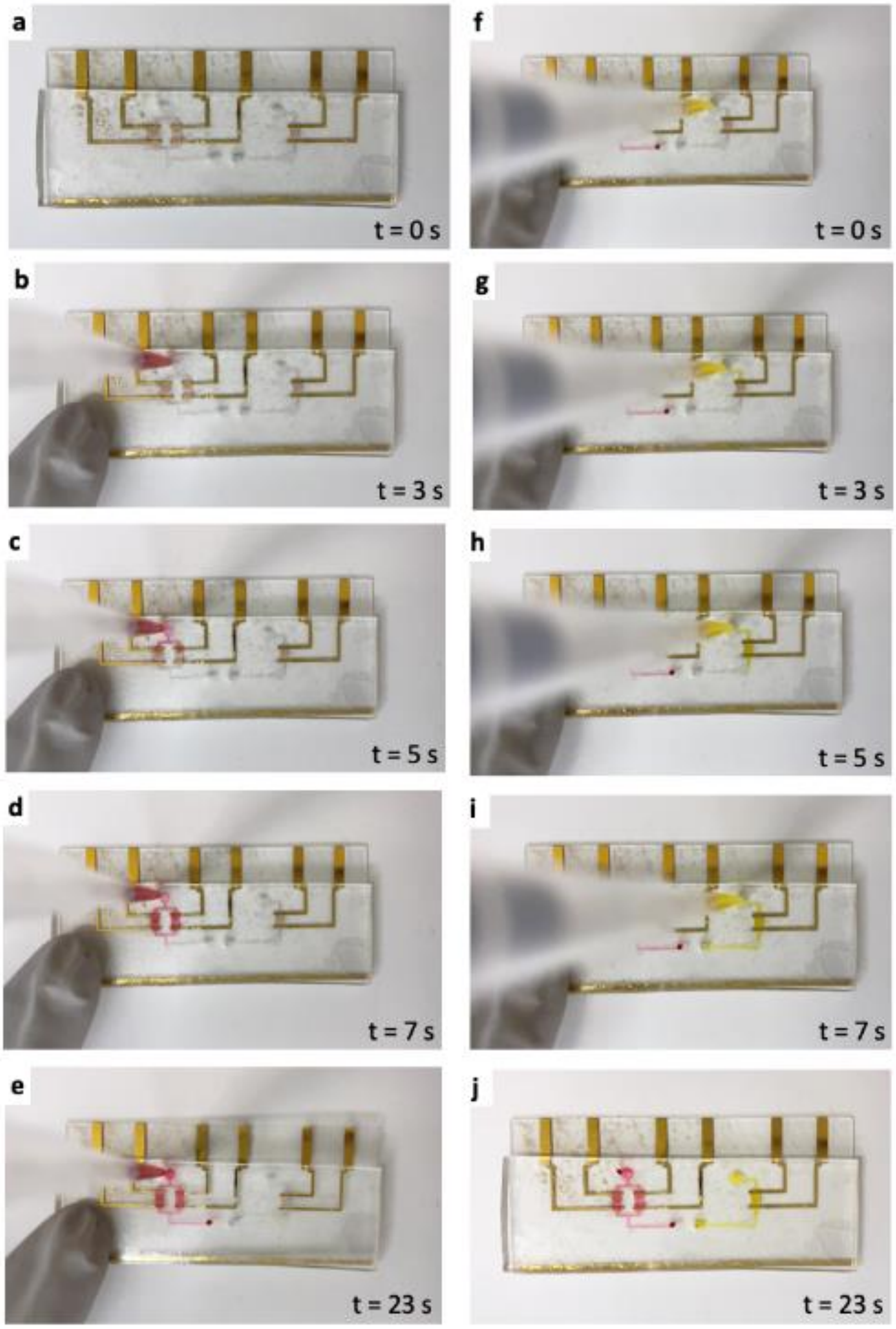

Figure S7. Food dye demonstration of fluid displacement in the microchannels of the electrochemical microfluidic device. (a) Image of the empty electrochemical microfluidic device, (b-e) time lapse images of red food dye displacement via pipet injection through the blood microchannels, (f-i) time lapse images of yellow food dye displacement via pipet injection through the saliva microchannel, (j) final image of the filled microchannels showing no mixing and spatial distinction between the fluids.

COMSOL simulations of fluid flow

The flow profiles of the proposed fluidic channels are evaluated using COMSOL Multiphysics with geometries imported from AutoCAD (**Figure S8**). The implementation of suction-based flow via flexible PDMS buttons allows for pressure to become a function of the compressed volume (**Equation S1**).

$$P = \left(\frac{V_0}{V} - 1\right) P_{atm} \qquad \textbf{Equation S1}$$

Where P is the negative pressure caused by the suction-button deformation, V_0 is the volume of the undeformed pressure chamber, V is the volume of the deformed pressure chamber and P_{atm} is the atmospheric pressure.[10] Notably, $\left(\frac{V_0}{V} - 1\right)$ expresses the compression that causes negative pressure-driven flow with the volumes calculated from the suction button (diameter: 2.5 mm, height: 1 mm). We assumed that the suction button is compressed almost completely (± 5% uncompressed); this yielded a pressure drop of about 5000 Pa, which was input as the negative pressure at the device outlets. Blood was simulated with a density of 994 kg. m^{-3} and dynamic viscosity of 0.004 Pa. s, while saliva was simulated with a density of 1012 kg. m^{-3} and a dynamic viscosity of 0.00157 Pa. s.[11–13] The 3D simulation based on these conditions yielded a velocity distribution shown in **Figure S8a**; surface integration of the velocity magnitude yielded a volumetric flow rate of 0.801 mL. s^{-1}, which is reasonable given that the channels have volumes of ~1 mL. The velocity in the chamber containing the assay remained low, which can improve the detection of proteins in the targeted biofluids.[14] The analysis assumed creeping flow of incompressible fluids with unchanging material properties in a stationary simulation. To determine the effect of creeping flow on the microfluidic-based electrochemical assay, we assumed based on the scanning electron microscopy (SEM) analysis (**Figure 2b**) that the assay height was 2 μm and the RE/CE connection for on-chip detection height was 5 μm; only a 50 μm by 25 μm region of interest was studied.

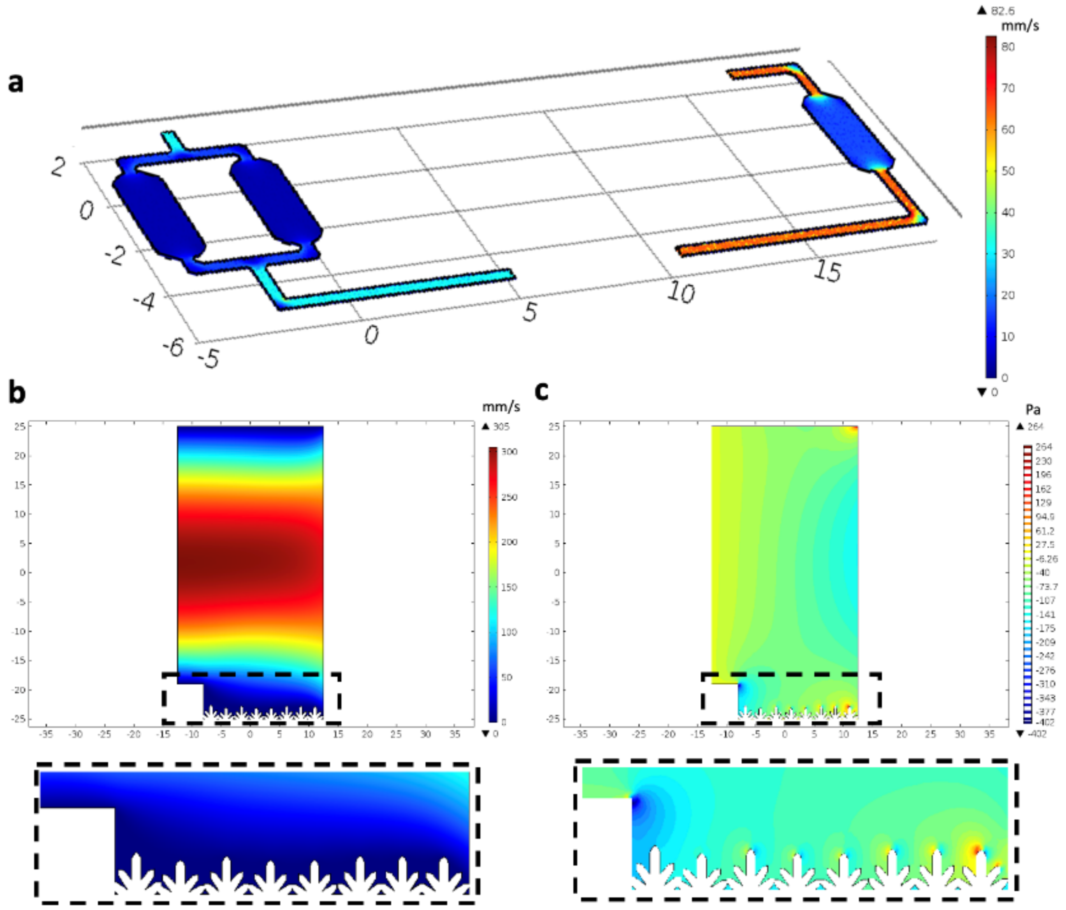

Figure S8. COMSOL simulations of fluid flow. (a) 3D simulation of proposed fluidic channels showing velocity distribution on the xy-plane; axis is shown in millimetres. The velocity in the chamber containing the assay remained low, which can improve the detection of proteins in the targeted biofluids. (b) 2D cross-sectional velocity distribution in the yz-plane to account for the hierarchical profile of the nanostructured assay and demonstrated that the velocity is lowest near the surface of the assay; therefore, the sensitive nanostructured layer should remain undisturbed. The higher velocity at the center of the cross-section demonstrated a parabolic velocity profile, which was indicative of Hagen-Poiseuille flow; inset: zoom-in view of the assay. (c) The corresponding pressure distributions demonstrated the effect of the RE/CE connection in the chamber as shielding the assay from high pressure; in the proximity of the RE/CE connection, the incident pressure on the assay was dampened; zoom-in view of the assay. Only a 50 µm by 25 µm region of interest was studied.

1.3. Cost of NFluidEX device

The cost of the device was based on the fabrication of the printed circuit board, electrochemical microfluidic device, 3D-printed housing unit and the 3D-printed sample collection kit. The cost for one handheld signal transduction unit with a fully assembled PCB was $533, which is cheaper than current potentiostats used for EIS analysis.[29] Meanwhile, the price per test kit was calculated by considering all disposable components of the design, including the electrochemical microfluidic test strips and the sample collection kit; this yielded a cost of about $7 per kit. Compared to single-test screen printed electrode that costs $7,[30] the NFluidEX harnesses three test assays with fluidic channels for precise biofluid control. Assuming the scaled production of 10,000 units, the cost per whole device drops to $199 and the cost per test kit would be $2.69 to enable low-cost accessibility that reveals the potential for scalable manufacturing. Notably, this represents the cost for the service provider; we can estimate a cost lower than $10 per test for the end user once the product is packaged and in used in congregate settings, at-home and in remote locations. The NFluidEX dual detection device allows for both diagnostic and serology testing on the same device; based on a cost analysis of current testing paradigms, a proposed device would be cost-effective for under $75 per test for all transmission rates,[31] thereby confirming the feasibility of NFluidEX as an economical solution. The cost breakdown for the whole device is summarized in **Table S1**; all prices are given in Canadian dollars and assumes the use of research research-grade materials and fabrication protocols.

Table S1. Cost breakdown for NFluidEX device

**Part**	**Bulk cost**	**Quantity**	**Cost per unit**	**Vendor**
Electrochemical Microfluidic Device				
Microfluidic device fabrication	ITO, aluminum deposition for alignment markers, SiO_2 deposition and lithographies, etching and dicing, lift-off, and precious metal deposition; PDMS bonding	~ 5 hours total	$6.41	Cleanroom facilities
Test assay	$579 per 5 grams	3 electrodes per device	$0.0405	Sigma-Aldrich, Alfa Aesar

Suction buttons	$109 per 0.5 kilograms	1 ml per device	$0.21	Dow SYLGARD
Sample Collection Kit				
PLA black resin	$24.95 per 800 ml	16 ml per device	$0.50	Filaments.ca
Whatman Grade 4 filter	$1.15 per 10 filters	1/4 filter per device	$0.029	Whatman
**Total[a]**	**$7.19 per test kit**			
Handheld Signal Transduction Housing Unit				
PLA black resin	$24.95 per 800 ml	240 ml per device	$7.49	Filaments.ca
Printed Circuit Board and Signal Transduction Components				
Printed circuit board (fully assembled)	$392.77 per board	1 per device	$392.77	PCBWay
Battery	$14.99 per battery	1 per device	$14.99	Canada Robotix
Relay module	$26.49 per 5 modules	1 per device	$5.30	Amazon
Screen-printed electrode adaptors	$31.49 per adaptor	3 per device	$94.47	IORodeo
3-way manual toggle switch	$6.15 per 5 switches	1 per device	$1.23	Amazon
3-way servo splitter cable	$16.99 per cable	1 per device	$16.99	Amazon
Jumper wires	$11.98 per 240 wires	3 per device	$0.15	Amazon
**Total[b]**	**$533.39 per signal transduction module**			

[a] Per test kit includes the cost of all disposable components, including the electrochemical microfluidic device and sample collection kit. Scaling the cost for the fabrication of 10,000 devices over one month assumes the adoption of certain McGill University cleanroom policies. This includes a soft cap at monthly expenses of $2000 with subsequent expenses reduced to 25% of the original cost. Assuming the use of 12” wafers in scalable production, the 10,000 devices can be fabricated using 223 wafers; with the cleanroom soft cap policy

pricing, this can yield a cost of $19,230.77 for 10,000 devices and we estimate the total price per test can be down to $2.69.

[b] Whole device includes all reusable components, including the handheld signal transduction unit and its associated components. Assuming 10,000 devices are fabricated, we estimate the price can be down to $199.

1.4. The NMI/MIP Assay

Assay optimization using Finite Element Method (FEM)

A FEM simulation was performed using COMSOL Multiphysics to show the effect of the NMIs on electrochemical sensing. A single NMI was designed in SolidWorks 2021 based on SEM images (**Figure 2b);** we assumed a base with a 1 µm diameter and 3.14 µm^2 surface area, then designed eight protrusions separated by an equal angle (45 degrees) with 2 µm height. The electric current physics is used to solve the current conservation equation based on Ohm's law. A reference electrode was implemented by applying zero potential at infinity using an Infinite Element Domain boundary condition. Then, the molecularly imprinted polymer (MIP) layer made from nonconductive o-PD was modelled by first determining its impedance value. In general, an impedance value depends on the resistance and the capacitance of the electrode surface.[15] To determine the impedance of the o-PD layer, we used a control sample (**Figure S9a, inset**) as an experimental calibration to determine the unknown parameters. First, we considered the capacitance to have a negligible effect on the impedance value at high frequency (10^5 Hz) (i.e., the impedance magnitude is equal to the resistance parameters). Then, we applied known impedance values for a single gold NMI (2.96 µΩ for an active surface area of 0.49 cm^2).[16] The resultant obtained resistance and capacitance parameters for o-PD were 4.5e-4 Ω. µm^2 and 14 F. µm^{-2}, respectively.

With these known values, we assessed the role of the NMIs on electrochemical biosensing by evaluating the surface current density against that of a bare gold electrode. The simulation results showed that current density was increased more than five times by adding NMI structures (**Figure S9a**), which was likely due to the higher geometric aspect ratio and isotropy of the protrusion surface. The sharp edges of the NMI electrodes provided a steep electric field gradient, which enabled a higher electrical current. Also, the NMI structures provided 10 times higher surface area (28.9 µm^2 versus 2.82 µm^2) for a high surface-to-volume ratio, resulting in predictably enhanced electrochemical biosensing.

Observing the corresponding impedance of the simulated electrode over the frequency sweep demonstrated the highest impedimetric response for low frequencies values, particularly at 0, 0.1 and 0.01 Hz (**Figure S9b**). As such, the most sensitive response was expected to occur over these low probing frequencies.

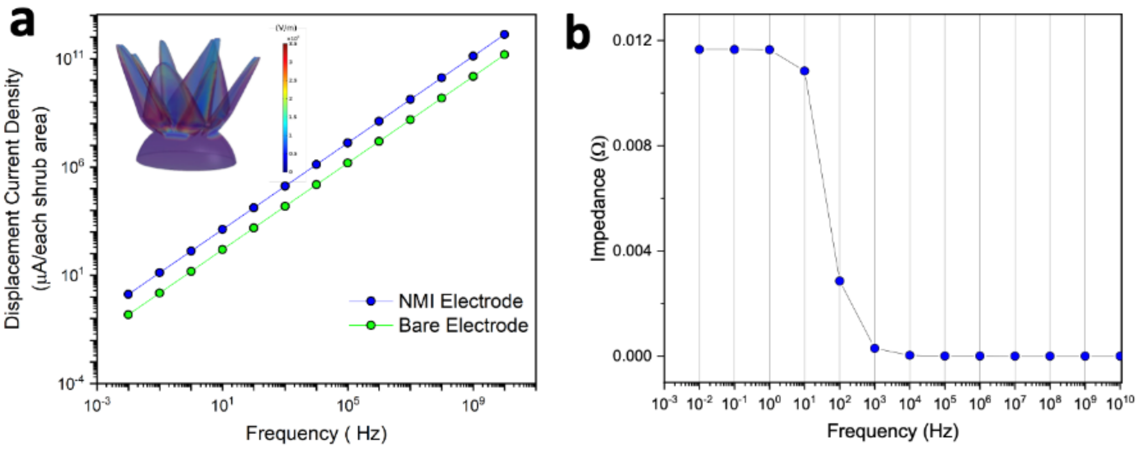

Figure S9. The effect of NMI electrode on current density. (a) The current density is increased more than 2 times by using NMI structures; the inset shows the electrical field over a single NMI structure. (b) The impedance of the electrode as a function of the probing frequency showing the highest response in the low frequency range.

Fabrication optimization

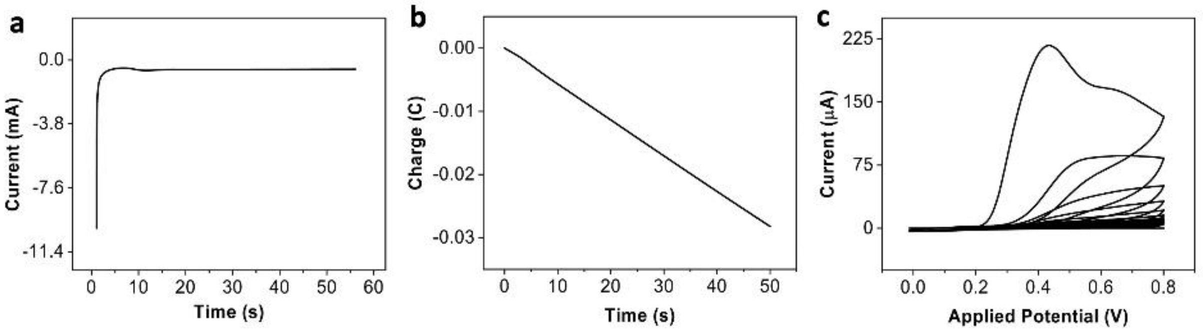

Figure S10. NMI/MIP assay fabrication. Chronoamperometry was performed to fabricate gold NMIs[17] and cyclic voltammetry to electropolymerize nonconductive o-PD polymer to fabricate the MIP assay with various template proteins including the SARS-CoV-2 spike protein (SP) and anti-receptor binding domain (RBD) antibodies (IgG-RBD and IgM-RBD). (a) Gold NMIs electrodeposition using chronoamperometry, (b) corresponding charge on the working electrode during electrodeposition becoming more negative, (c) cyclic voltammetry for o-PD electropolymerization. 25 successive cycles of the electropolymerizing were done in the presence of template proteins on the NMIs surface. Two oxidation peaks are observed in the first cycle at about 0.4 and 0.7 V, which are related to the oxidation of o-PD;[14] from the second to the tenth cycle, just one oxidation peak exists, which gradually shifts to more positive potentials, and its intensity decreases mainly due to the formation of a nonconductive layer on the surface.[15] In the last cycle, the oxidation peaks of o-PD have completely disappeared, validating the creation of a continuous nonconductive layer on the surface.

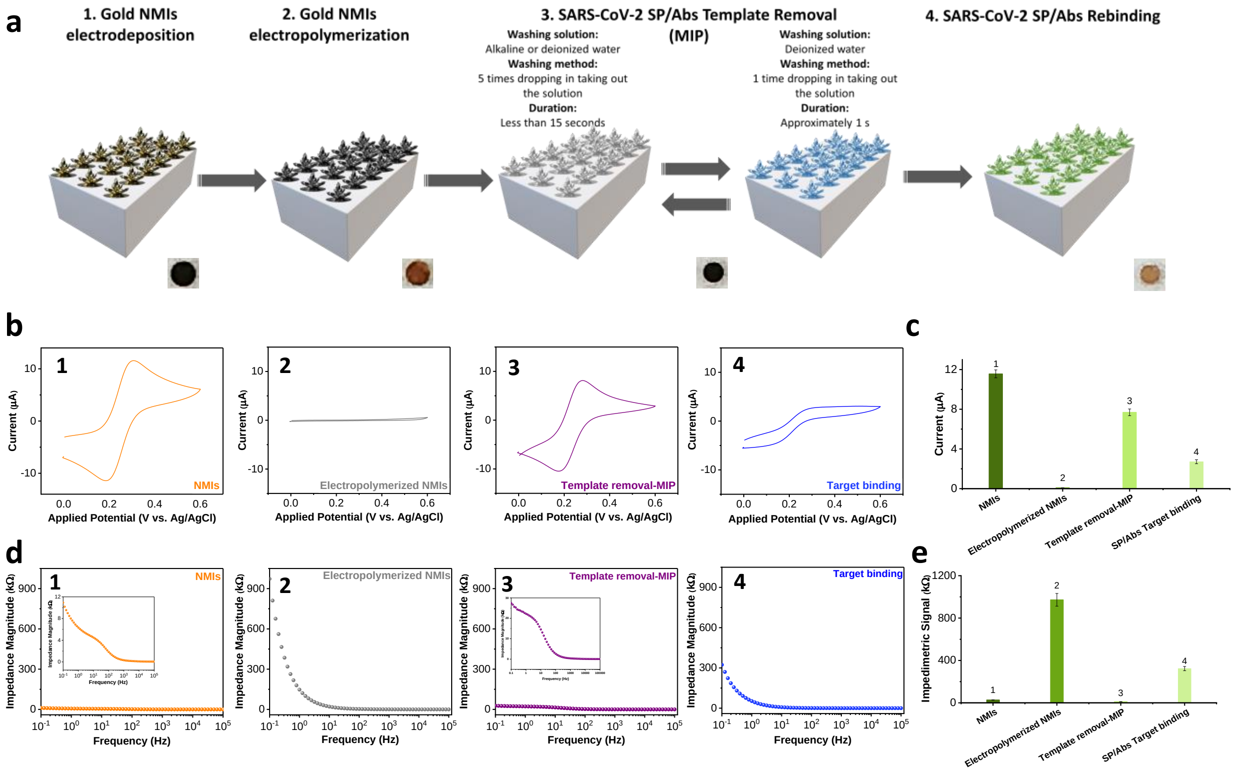

Figure S11. Electrochemical characterization of NMI/MIP assay. Electrochemical characterization of the NMI/MIP assay after each step of electrode fabrication. (a) The stepwise fabrication process for each assay (SP and antibodies): (1) electrodeposition of the enhanced gold NMIs, (2) electropolymerization of the nonconductive o-PD polymer, (3) template removal from the MIPs, (4) target binding, (b) the cyclic voltammetry responses at each step; the redox peak for NMIs is sharply suppressed in the presence of a nonconductive polymer to confirm the coverage with o-PD during electropolymerization, (c) the current signal at 0.3 V at each fabrication step, (d) the Bode EIS response at each step, and (e) the corresponding impedance signal at 0.1 Hz for each step; the highest sensitivity of the assay is in the low frequency range, with the greatest difference in impedimetric response at 0.1 Hz. The electrochemical measurements were done with 5 mM $[Fe(CN)_6]^{3-/4-}$ in PBS. After electropolymerization, the current is decreased, and impedance magnitude is increased due to complete coverage of the electrode surface with nonconductive o-PD. Template removal shows an increase in current confirming the partial coverage. A measurable drop in the current and consequent increase in impedance magnitude is resulted from target binding to the surface.

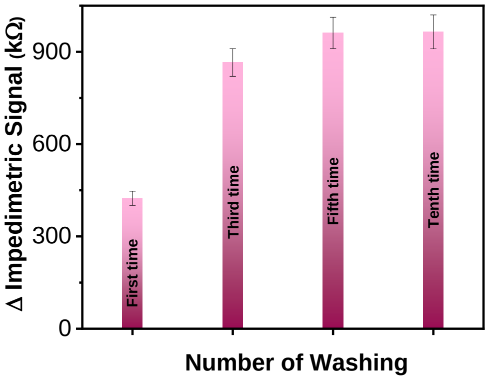

Figure S12. Optimization of the washing step in template removal to achieve the optimal number of binding sites. The impedance magnitude differences with respect to the electropolymerized NMIs after various number of washing repeats with ethanol and water (5:1) and 0.1 M NaOH (optimal washing solution). After five times washing, most of the template proteins were removed from the structure.

1.5. Physical Characterization of Test Assay

Molecular docking simulation and characterization of o-PD

Due to the large sequence length of the 6VXX SP, we used 24 boxes to investigate docking events (**Table S2**); for the smaller 7BWJ antibody fragment, we used 2 boxes (**Table S3**). For the 6VXX SP, preferential binding is exclusively in the head region of the SP, indicating that the favourable binding is not solely based on ligand charge or structure. Due to many o-PD monomers in the same region, the monomers are likely competing for residues in the head region. This also suggests that the protein is not confined in the polymer as only the top region is making an imprint to impart chemical functionality. Similar results are shown for the 7BWJ antigen-binding fragment; for a 2-box simulation, 7 o-PD monomers found preferential binding sites, indicating favourable and competitive interaction between the o-PD monomers and the amino acids. We also observe that the amine functional groups in o-PD preferentially face the Phe and His amino acids to make stabilizing hydrogen bonds and electrostatic interactions. Frequent interactions with polar and charged amino acids were observed, including Ser, Lys, Asp, Arg and Gln. This confirms that o-PD is a suitable option for making MIPs with SARS-CoV-2 SP and antibodies, and suggests chemical functionality is imparted in the construction of MIPs, allowing for increased selectivity during detection.

Table S2. Molecular docking box coordinates for the 6VXX SP in o-PD with the corresponding free energy of preferential binding.

Box Number	Coordinates (x, y, z)	DG (kcal/mol)
Box 1	(187, 173, 253)	-5.66
Box 2	(208, 173, 250)	-5.66
Box 3	(239, 173, 250)	-5.70
Box 4	(239, 174, 250)	-5.76
Box 5	(239, 173, 250)	-5.67
Box 6	(250, 208, 249)	-5.71
Box 7	(231, 194, 247)	-5.71
Box 8	(231, 230, 246)	-5.68
Box 9	(199, 243, 247)	-5.68
Box 10	(194, 181, 247)	-5.69
Box 11	(189, 174, 224)	-4.57
Box 12	(189, 174, 196)	-4.54
Box 13	(253, 197, 224)	-5.67
Box 14	(214, 217, 224)	-5.68
Box 15	(186, 217, 224)	-5.72
Box 16	(186, 217, 224)	-4.48
Box 17	(213, 247, 224)	-5.71
Box 18	(190, 250, 204)	-4.55
Box 19	(185, 225, 204)	-5.47
Box 20	(220, 235, 204)	-5.44
Box 21	(235, 220, 204)	-5.45
Box 22	(205, 220, 204)	-5.45
Box 23	(185, 205, 204)	-5.41
Box 24	(185, 166, 204)	-4.53

Table S3. Molecular docking box coordinates for the 7BWJ SP RBD antibody in o-PD with the corresponding free energy of preferential binding.

Box Number	Coordinates (x, y, z)	DG (kcal/mol)
Box 1	(15, -35, 0)	-4.93
Box 2	(15, -65, 0)	-4.93

Atomic force microscopy (AFM) to evaluate template removal

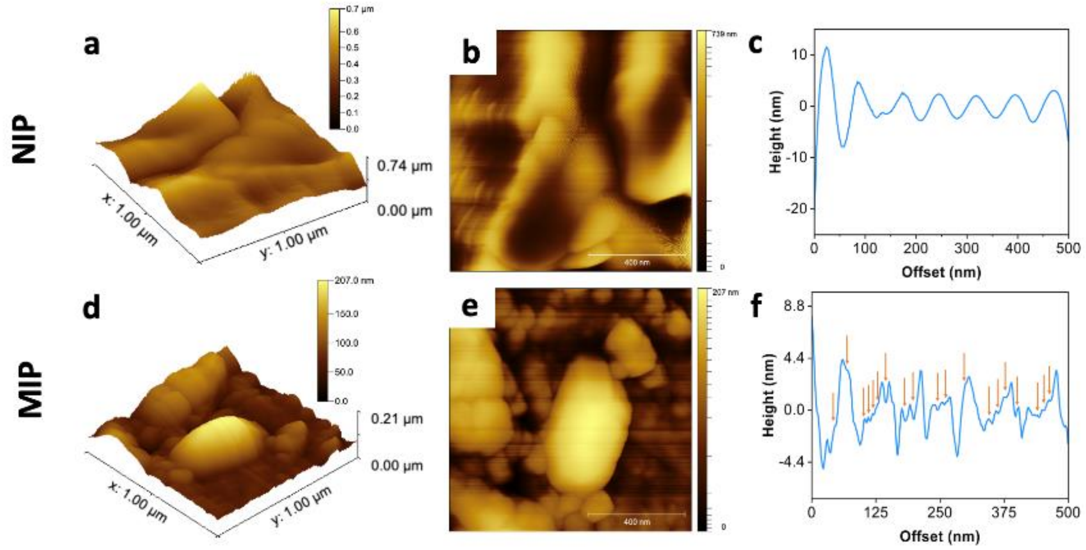

Figure S13. AFM scans of the NFluidEX after template removal. (a) The 3D topology of the NIP electrode. (b) The 2D profile of the NIP electrode. (c) The one-dimensional nanoroughness of the NIP electrode. (d) The 3D topology of the MIP electrode. (e) The 2D profile of the MIP electrode, (f) The one-dimensional nanoroughness of the MIP electrode with orange arrows showing the imprinted recognition sites. The imprinted MIP possesses nanometer-scale recognition sites in the o-PD polymer on the surface compared to relatively smoother nonimprinted polymer (NIP) surface; the AFM profiles were analyzed with the Gwyddion probe microscopy visualization and analysis software using the 500-nm line spectroscopy one-dimensional roughness tool at the cut-off of 0.05 Nyquist frequency.

Assay incubation time

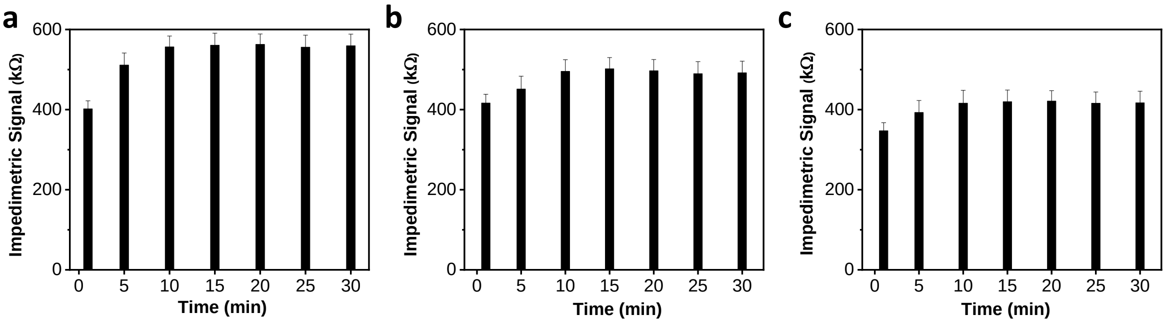

Figure S14. The assay incubation time study. The impedimetric signal at different incubation time for SARS-CoV-2 (a) SP in human saliva at 1000 pg. ml^{-1}, (b) IgG-RBD in undiluted human plasma at 100 pg. µl^{-1}, and (c) IgM-RBD in undiluted human plasma at 100 pg. µl^{-1}. As such, 10 min is considered as the optimal incubation time as negligible differences were observed after 10 min incubation. For the whole blood, the incubation period was determined to be 1 min to prevent coagulation on the surface of the electrode to avoid an erroneously high impedimetric readout due to the presence of coagulated blood aggregates.

Stability, repeatability, and reproducibility of the NMIs/MIPs biosensor

The stability of the NMI/MIP and NMI/NIP sensors was evaluated using cyclic voltammetry in the presence of 5 mM [Fe(CN)6]3-/4- in 1X PBS. During a total of 100 cycles, a nearly unchanged current signal and the same curve shape were observed, indicating the high electrode stability for the NMIs (**Figure S15a**), MIPs (**Figure S15b**) and NIPs (**Figure S15c**). This not only demonstrates the high stability of the composite electrode, but further emphasizes that the initial NMI electrode served as a stable and controllable electroactive surface for the electropolymerization of o-PD, both in the presence and absence of a template protein, resulting in an overall stable electrochemical detection platform. The stability was further validated over several days in ambient temperature, indicating the potential for long-term stable use and prolonged storage; 2.5-6% and 5-9% decrease in the electrochemical response after 7 and 21 days, respectively, were captured for SARS-CoV-2 SP on NMI/MIP electrode in saliva and SARS-CoV-2 IgG-RBD and IgM-RBD on NMI/MIP electrode in whole blood (**Figures S15d-f**). These results signify that the proposed biosensor offers high stability at ambient temperature. To ensure the absence of carryover effects upon successive measurements on a single sensor the repeatability of the biosensor was evaluated by the impedimetric repetitive measurements (five times, n=5). Each experiment was repeated for three individual electrodes and the relative standard deviation (RSD) was recorded 5.2%, which is in an acceptable range. Another important feature for practical applications of the biosensor is the reproducibility which was verified by recording the impedance magnitude for 5 as-prepared NMI/MIP electrodes, each one three times with a total RSD of 4.2% (n=5). A slight high value of this parameter can be related to the effective distribution of the MIP layer and its thickness.

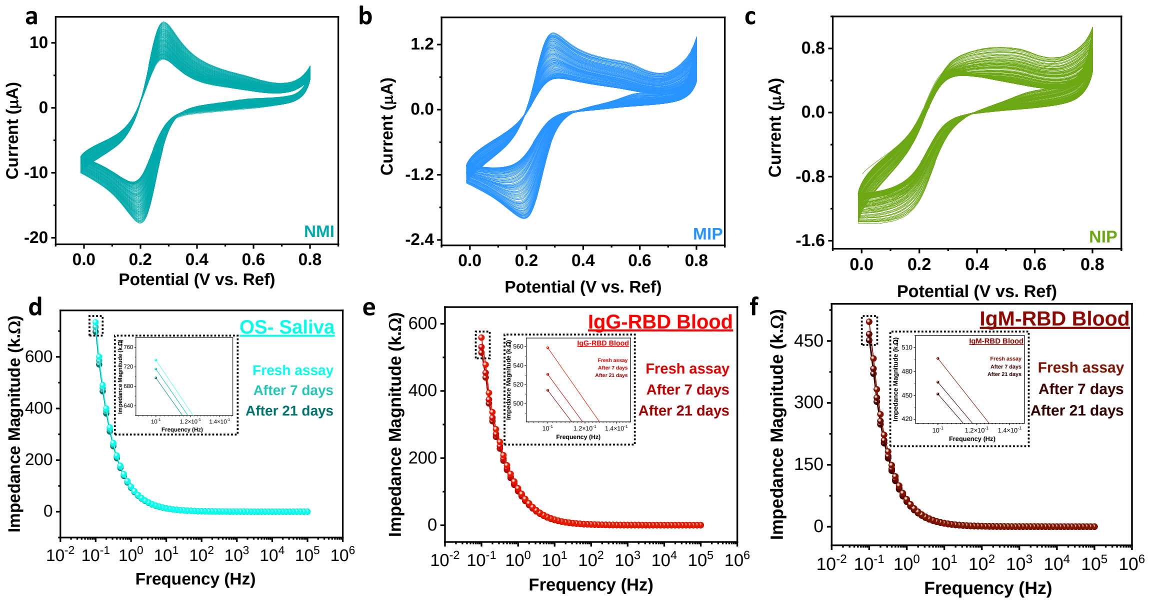

Figure S15. The electrochemical stability test for NMI/MIP assay. Cyclic voltammetry with 100 consecutive cycles for the (a) bare NMI, (b) NMI/MIP, and (c) NMI/NIP assay. Electrochemical measurements were done with 5 mM $[Fe(CN)_6]^{3-/4-}$ in PBS. The impedance magnitude of (d) SARS-CoV-2 SP on NMI/MIP electrode in saliva and (e) SARS-CoV-2 IgG-RBD on NMI/MIP electrode, and (f) SARS-CoV-2 IgM-RBD on NMI/MIP electrode in whole blood (insets show the zoom-in view).

2. NFluidEX Test Assay Assessment

Sensitivity

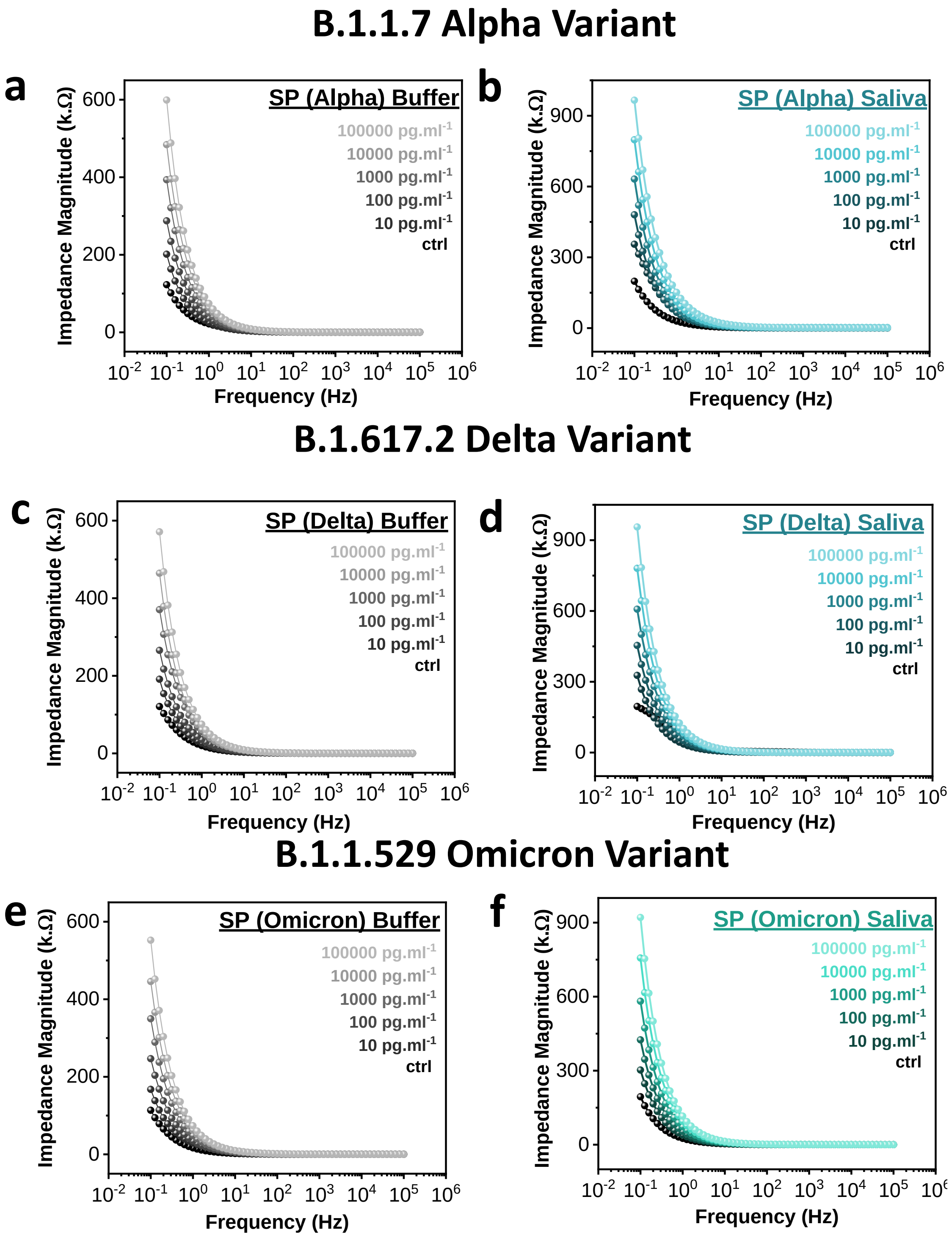

Figure S16. Sensitivity of variant spike proteins. Bode plots of the impedance magnitude over a relevant range of 10 pg. ml^{-1} -10^{5} pg. ml^{-1} for the Alpha B.1.1.7 variant SP in (a) buffer and (b) saliva, the Delta B.1.617.2 variant SP in (c) buffer and (d) saliva, and the Omicron B.1.1.529 variant SP in (e) buffer and (f) saliva.

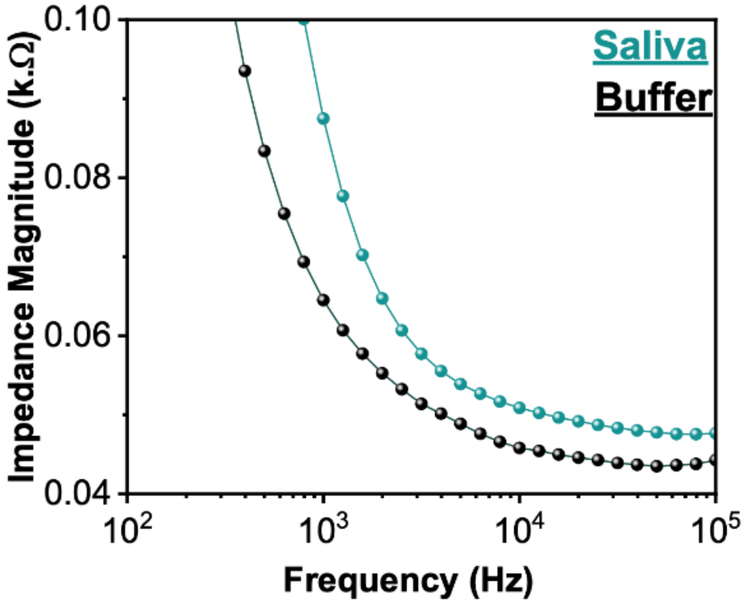

Figure S17. Comparison between the impedance magnitude of saliva and buffer, showing higher impedimetric responses for saliva compared to a 1X PBS buffer solution.

Table S4. Limit of detection and linear range of NFluidEX for various targets in saliva, plasma, blood, and buffer

Target	Biofluid	Limit of detection	Linear range
Original strain SP	Saliva	5.89 pg. ml^{-1}	1e1 – 1e5 pg. ml^{-1}
	Buffer	3.79 pg. ml^{-1}	
Alpha variant SP	Saliva	6.48 pg. ml^{-1}	
	Buffer	4.51 pg. ml^{-1}	
Delta variant SP	Saliva	8.13 pg. ml^{-1}	
	Buffer	6.28 pg. ml^{-1}	
Omicron variant SP	Saliva	7.62 pg. ml^{-1}	
	Buffer	4.72 pg. ml^{-1}	
Heat-inactivated SARS-CoV-2 viral particles	Saliva	948.4 number of viral particles. ml^{-1}	9.60e3 – 3.84e8 number of viral particles. ml^{-1}
	Buffer	2091.6 number of viral particles. ml^{-1}	
IgG-RBD	Plasma	4.06 pg. μl^{-1}	1e1 – 1e4 pg. μl^{-1}
	Blood	5.74 pg. μl^{-1}	
	Buffer	3.63 pg. μl^{-1}	
IgM-RBD	Plasma	2.97 pg. μl^{-1}	
	Blood	3.13 pg. μl^{-1}	
	Buffer	2.79 pg. μl^{-1}	
IgG-N	Plasma	6.94 pg. μl^{-1}	
	Blood	7.76 pg. μl^{-1}	
	Buffer	5.18 pg. μl^{-1}	
IgM-N	Plasma	3.25 pg. μl^{-1}	
	Blood	3.58 pg. μl^{-1}	
	Buffer	2.99 pg. μl^{-1}	
Influenza A SP	Saliva	8.63 pg. ml^{-1}	1e1 – 1e5 pg. ml^{-1}
	Buffer	3.99 pg. ml^{-1}	
Heat-inactivated Influenza A viral particles	Saliva	2,576,415 number of viral particles. ml^{-1}	6.44e6 – 2.58e9 number of viral particles. ml^{-1}
	Buffer	1,105,422 number of viral particles. ml^{-1}	

Table S5. Comparative table for detection of SARS-CoV-2 antigen and antibodies.

SARS-CoV-2 Tests	Media	Time	Limit of Detection	Linear Range	Portable signal transduction	Without Reference Measure	Ref
Magnetic bead-based immunosensor combined with carbon black-modified screen-printed electrode	Untreated saliva, buffer	30 min	SP: 1.9e4 pg. ml^{-1}	SP: 1.9e4 – 1e7 pg. ml^{-1}	Yes	Yes	[18]
ePAD paper-based sensor: GO-Modified with SP RBD immobilized and SKI bloqued	Clinical sera	30 min	SP: 110 pg. ml^{-1} IgG: 0.96 pg. µl^{-1} IgM: 0.14 pg. µl^{-1}	SP: 1000 – 1000e3 pg. ml^{-1} IgG and IgM: 1 - 1000 pg. µl^{-1}	No	No	[19]
NanoSystem: GO-8H-EDC–NHS–Au NS	Blood, saliva and nasal swab	1 min	SP: 1.68e-16 pg. ml^{-1}	SP: 1 - 10e-11 pg. ml^{-1}	No	Yes	[20]
Tethered Au nanostructurated bearing an analyte-binding antibody	Unprocessed saliva	5 min	SP: 1 pg. ml^{-1}	SP: 1 – 100 pg. ml^{-1}	No	Yes	[21]
SPEEDS: electrochemical immunosensor	Patient serum	13 min	IgG-S: 10.1 pg. µl^{-1} IgM-S: 1.64 pg. ml^{-1}	IgG-S: 10.1 – 6e4 pg. ml^{-1} IgM-S: 1.64 – 5e4 pg. µl^{-1}	Yes	Yes	[22]

SARS-CoV-2 RapidPlex: Laser engraved graphene electrodes	Serum and saliva	1 min	IgG-S: 250 pg. μl^{-1} IgM-S: 250 pg. μl^{-1}	IgG-S: 2e4 – 4e4 pg. μl^{-1} (serum), 200 – 500 pg. μl^{-1} (saliva) IgM-S: 2e4 – 5e4 pg. μl^{-1} (serum), 600 – 500 pg. μl^{-1} (saliva)	Yes	No	[23]
Electrochemical aptamer-based sensor	Serum and artificial saliva	Not stated	SP: 760 pg. ml^{-1}	SP: 760 – 760e3 pg. ml^{-1}	No	No	[24]
Low-cost Electrochemical Advanced Diagnostic (LEAD): modified graphite leads	Saliva	6.5 min	SP and Alpha variant: 0.229 pg. ml^{-1}	SP and Alpha variant: 0.1 - 1e3 pg. ml^{-1}	No	No	[25]
Carbon nanotube field-effect transistor	Saliva and buffer	2-3 min	SP: 4.12e-3 pg. ml^{-1}	SP: 0.1e-3 - 5.0 pg. ml^{-1}	No	No	[26]
DSA1N5-Cov-eChip: aptamer functionalized to gold electrodes	1:1 diluted saliva	10 min	Original strain: 0.438 pg. ml^{-1} Alpha variant: 1.227 pg.	Original, Alpha and Delta variants: 1.752 - 1927.2 pg. ml^{-1}	Yes	No	[27]

			ml^{-1} Delta variant: 1.578 pg. ml^{-1}				
KAUSTat AuNPs-LSG sensor	Nasal swab	1 min	SP, Alpha, Beta and Delta variants: 5140 pg. ml^{-1}	SP, Alpha, Beta and Delta variants: 1e3 – 500e3 pg. ml^{-1}	Yes	No	[28]
Flexible organic electrochemical transistors	Buffer, serum, saliva	5 min	IgG: 1.5e-4 pg. μl^{-1} (buffer), 1.5e-3 pg. μl^{-1} (saliva, serum)	IgG: 1.5e-3 – 1.5e3 pg. μl^{-1}	Yes	No	[29]
**NFluidEX: NMI/MIP assay**	**Untreated saliva Whole blood**	**11 min**	**See Table S4**	**See Table S4**	**Yes**	**Yes**	**This work**

Table S6. Comparative table of SARS-CoV-2 FDA EUA Approved Antigen Diagnostic Tests

Company	Test	Read-out	Sampling method	PPA	NPA	LOD	Respo-nse time	Number of Patient Samples	Ref
Abbott	Panbio COVID-19 Ag Rapid Test Device	Colori metric	Nasal swab	98.1%	99.8%	$2.5x10^{1.8}$ $TCID_{50}.ml^{-1}$	15 minutes	508	[30]
Access Bio Inc.	CareStart COVID-19 Antigen Home Test	Colori metric	Nasal swab	87%	98%	$2.8x10^{3}$ $TCID_{50}.ml^{-1}$	10 minutes	92	[31]
OraSure Technolog ies Inc.	InteliSwab COVID-19 Rapid Test Rx	Colori metric	Nasal swab	84%	98%	$2.5x10^{2}$ $TCID_{50}.ml^{-1}$	30 minutes	165	[32]
Lumira	LumiraDx SARS-CoV-2 Ag Test	Fluore scent	Nasal swab	97.6%	96.6%	32 $TCID_{50}.ml^{-1}$	12 minutes	255	[33]
BTNX Inc.	Rapid Response COVID-19	Colori metric	Nasal swab	94.55%	100%	$2x10^{2.4}$ $TCID_{50}.ml^{-1}$	15 minutes	85	[34]
QIAGEN	QIAreach SARS-CoV-2 Antigen test	Fluore scent	Nasal swab	85%	99.05%	$3.80x10^{6}$ $TCID_{50}.ml^{-1}$	2-15 minutes	270	#
**NFluidEX**	**NMI/MIP assay**	**Electr oche mical**	**Saliva**	**100%**	**100%**	**14** $TCID_{50}.ml^{-1}$	**11 minute s**	**42**	**This wor k**

Table S7. Comparative table of SARS-CoV-2 FDA EUA Approved Serology Tests

**Company**	**Test**	**Detected Antibodies**	**PPA**	**NPA**	**LOD**	**Response time**	**Ref**
Access Bio, Inc.	Access Bio CareStart COVID-19 IgM/IgG	IgG-S, IgM-S, IgG-N and IgM-N	98.4% (combined)	98.9% (combined)	Not stated	10 min	[35]
Abbott	AdviseDx SARS-CoV-2 IgG II (Alinity)	IgG-S	98.1%	99.6%	~8.67 pg. μl^{-1}	Not stated	[36]
Siemens	Atellica IM SARS-CoV-2 IgG (COV2G)	IgG-S	100%	99.9%	~0.84 pg. μl^{-1}	2 h, 1 min batch testing	[37]
Kantaro Biosciences	COVID-SeroKlir	IgG-S	99.15%	99.6%	~3.14 pg. μl^{-1}	30 min	[38]
BTNX Inc.	Rapid Response Liberty	IgG-N, IgG-RBD, IgM-N and IgM-RBD	92.9-94.6%	98.1-100%	--	15 min	#
**NFluidEX**	**NMI/MIP assay**	**IgG-RBD, IgM-RBD, IgG-N, IgM-N**	**100%**	**100%**	**2.79-7.76 pg. μl^{-1} for IgG-RBD, IgG-N, IgM-N and IgM-RBD**	**11 min**	**This work**

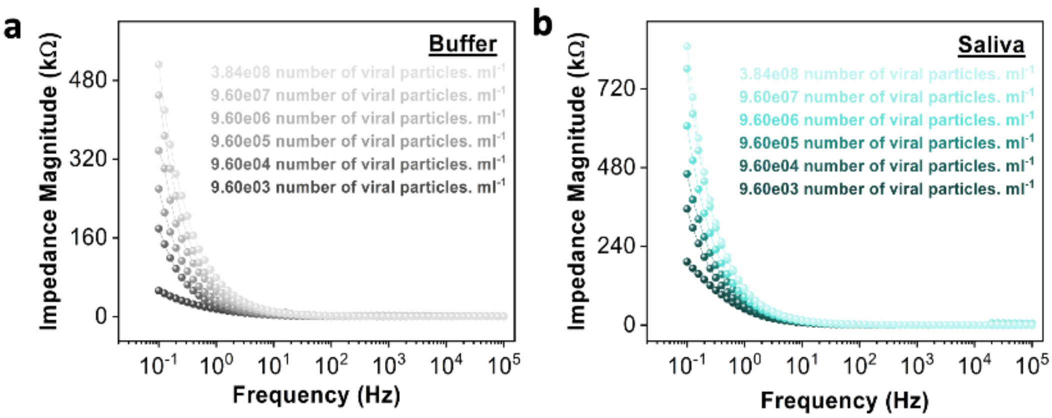

Figure S18. Bode plots for impedimetric detection of heat-inactivated SARS-CoV-2 viral particles in (a) buffer and (b) saliva from 9.60x10^3 – 3.84x10^8 number of viral particles. ml^{-1}. The highest sensitivity was recorded at 0.1 Hz in buffer and saliva.

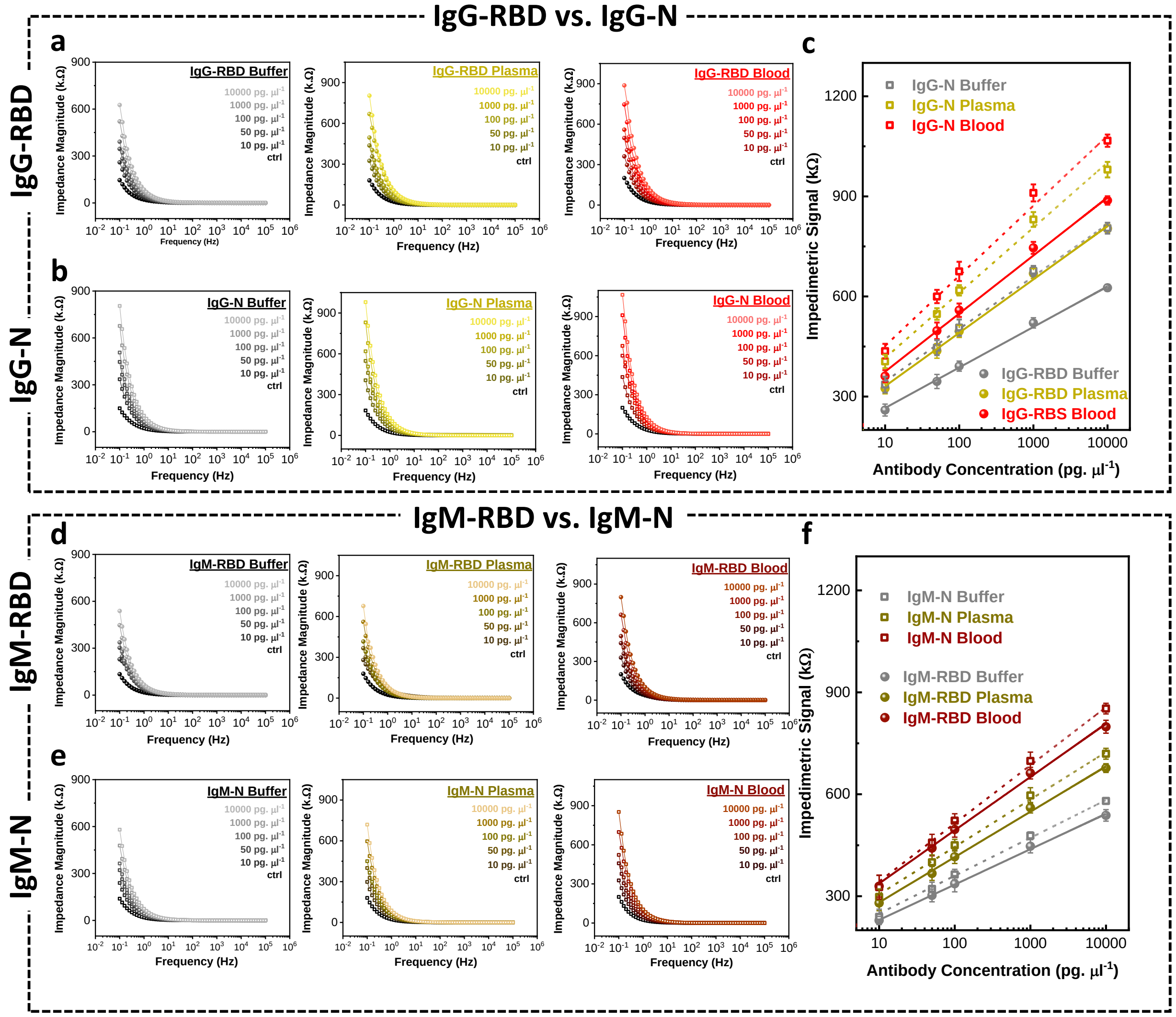

Figure S19. Calibration plots for serology in buffer, plasma, and whole blood of (a) IgG-RBD, (b) IgG-N and (c) corresponding calibration curve; calibration plots for serology in buffer, plasma, and whole blood of (d) IgM-RBD, (e) IgM-N and (f) corresponding calibration curve; targeted range from 10 pg. μl^{-1}- 10^4 pg. μl^{-1}. Comparable impedimetric responses were shown for both anti-RBD (IgG-RBD and IgM-RBD) and anti-nucleocapsid (IgG-N and IgM-N) antibodies in each of the three biofluids, demonstrating the functionality of the assay independent of the biofluid selection. Marginally higher impedimetric responses for whole blood are likely due to the presence of interferent molecules and blood cells,[39] but the electrochemical response remains within a comparable range of impedance magnitude.

Selectivity and Cross Reactivity

Table S8. A summary of statistical significance evaluation using one-way ANOVA with post hoc Holm-Sidak mean comparison test for the diagnostic selectivity of SARS-CoV-2 SP

Mean Comparisons	Saliva p value			Buffer p value			Sig.[a]
	**10 pg.ml^{-1}**	**1000 pg.ml^{-1}**	**10000 pg.ml^{-1}**	**10 pg.ml^{-1}**	**1000 pg.ml^{-1}**	**10000 pg.ml^{-1}**	
Influenza A H1N1 SARS-CoV-2	3.55E-11	4.02E-12	3.79E-14	1.79E-14	2.26E-14	1.75E-13	1
HCoV-229E SARS-CoV-2	4.58E-11	4.44E-12	4.69E-14	2.23E-14	2.96E-14	2.10E-13	1
MERS-CoV SARS-CoV-2	4.69E-11	5.33E-12	4.93E-14	2.33E-14	3.01E-14	2.22E-13	1
Influenza A H1N1 SARS-CoV-1	2.91E-10	5.70E-11	3.57E-13	1.70E-13	2.60E-13	1.56E-12	1
HCoV-229E SARS-CoV-1	3.98E-10	6.50E-11	4.65E-13	2.24E-13	3.68E-13	1.95E-12	1
MERS-CoV SARS-CoV-1	4.10E-10	8.25E-11	4.96E-13	2.36E-13	3.76E-13	2.09E-12	1
SARS-CoV-1 SARS-CoV-2	1.69E-04	4.71E-06	2.59E-07	1.23E-07	7.65E-08	1.33E-06	1
Influenza A H1N1 MERS-CoV	0.42109	0.31383	0.14863	0.12069	0.10126	0.25589	0
Influenza A H1N1 HCoV-229E	0.46224	0.51144	0.23302	0.18526	0.11884	0.38013	0
MERS-CoV HCoV-229E	0.94208	0.71207	0.77357	0.78995	0.92318	0.78	0
$F_{4,10}$ values	442.444	668.2656	1744.077	2025.4021	1896.4195	1293.194	

[a] Significant p values are denoted by a one (1) and non-significant p values are denoted by a zero (0).

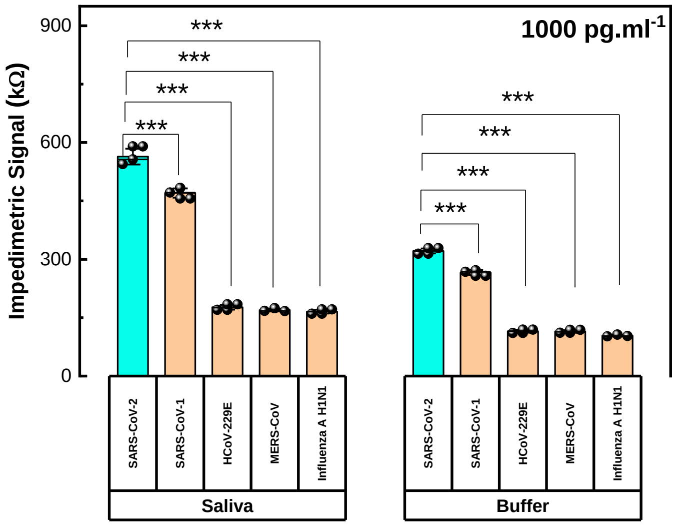

Figure S20. Selectivity over the linear range of SP detection in saliva and buffer at 1000 pg. ml^{-1}, *** $p < .001$. For the entire linear range from 10 pg. ml^{-1} -10^{5} pg. ml^{-1}, no cross reactivity of similar viruses was observed.

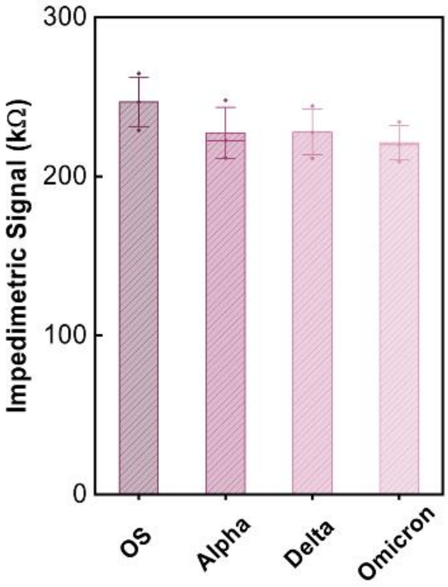

Figure S21. Cross reactivity study of SP assays for different variants: original strain, alpha, delta and omicron SPs at 100 pg. ml^{-1}. The SPs of variants were tested on the original strain nano-imprinted polymer assay, which demonstrated the ability to detect even minute changes in protein morphology. Although the original strain NMI/MIP assay can detect a positive result for emerging variants, the noticeable signal drop indicated the high resolution of the MIPs that can differentiate structural refinements based on single amino acid mutations.

Physical characteristics of proteins including their molecular weight, the radius of gyration and percentage sequence similarity provided insight about the predictive binding activity and the selectivity of the assay. Proteins were selected from the Protein Data Bank (PDB) and visualized with Visual Molecular Dynamics (VMD, version 1.9.3); the PDB files of the proteins were used to determine their physical properties. The radius of gyration (R_g) was used to understand the spread of a protein in space; the average R_g estimated the size of the protein and its corresponding polymer imprint. From the PDB file, we calculated this radius according to **Equation S2**.[40]

$$\langle R_g \rangle = \sqrt{\frac{1}{n}\sum_{i=1}^{n}(r_i - r_{cm})^2} \qquad \textbf{Equation S2}$$

where n is the number of particles defined to be the total mass divided by the mass of individual atoms, r_i is the coordinate of each atom and r_{cm} is the center of mass defined to be the average position of each atom. Interestingly, a higher impedance magnitude was recorded amongst proteins with a smaller R_g, indicating that smaller proteins can likely bind with greater ease and generate a more robust electrochemical response while larger proteins cannot easily diffuse out of the polymer layer following template removal;[41] similar trends between protein size and electrochemical response were reported in other electrochemical assays.[23] The percent sequence similarity was measured from the PDB file using the Pairwise Structure Alignment tool using the Smith-Waterman 3D algorithm (gap opening penalty = 3; gap extension penalty = 5).[42] The percentage of locally identical residues in addition to residues with similar chemical properties between two structures was reported as the percent sequence similarity.

Physical properties are summarized in **Table S9-S10**. Structures with a higher similarity showed better binding to the assay (ex. SARS-CoV-1 SP); nonetheless, the sequence and physical similarity between SARS-CoV-2 and other similar viruses were sufficiently distinct to generate statistically significant differences in the impedance magnitude.

Table S9. Physical properties of viral spike proteins

Name	Molecular Weight	Radius of Gyration	Structure	Mutations	Percent Sequence Similarity	Ref
SARS-CoV-2 spike protein (PDB 6VXX, 7V7Q, 7LWV, 7T9J)	438.26 kDa	9.8508 nm	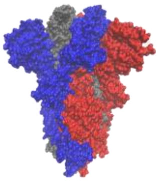	Original strain	100%	[43]
	444.07 kDa	9.259 nm	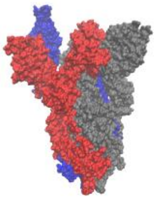	Delta B.1.617.2: High transmissibility with reduction in the neutralization potential	99%	[44,45]
	433.91 kDa	7.4003 nm	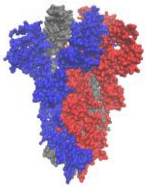	Alpha B.1.1.7: High transmissibility	99%	[46]
	440.26 kDa	9.4361 nm	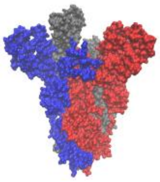	Omicron B.1.1.529: High transmissibility, homology with cold viruses (ex. HCoV 229E)	98%	[47,48]
SARS-CoV-1 (PDB 5XLR)	401.03 kDa	6.7966 nm	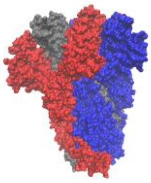	Wild type	92%	[49]

HCoV 229E spike protein (PDB 6U7H)	398.28 kDa	4.4818 nm	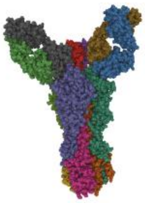	Wild type	54%	[50]
MERS CoV spike protein (PDB 5X5F)	437.57 kDa	4.953 nm	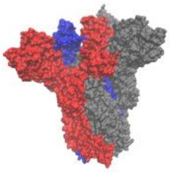	Wild type	57%	[51]
Influenza H1N1 hemagglutinin protein (PDB 1RUY)	165.02 kDa	4.3397 nm	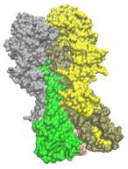	Wild type	51%	[52]

Table S10. Physical properties of humoral immune response antibodies

**Name**	**Structure**	**Molecular Weight**	**Radius of Gyration**	**Antibody Characteristics**	**Ref**
IgM antibody (PDB 2RCJ)	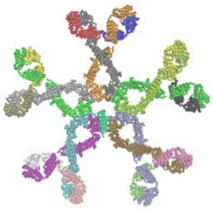	809.95 kDa	11.6187 nm	First antibodies that are produced, low affinity binding, but compensate with pentamer antigen binding sites	[53]
IgG antibody (PDB 1IGY)	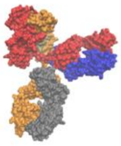	146.10 kDa	4.687 nm	Principle neutralizing antibodies, high affinity viral binding	[54]

Antigen-Binding Fragments (FABs)

**Name**	**Binding domain**	**Target protein**	**Percent Sequence Similarity**	**Ref**
Anti-SARS-CoV-2 antibodies	Receptor binding domain (PDB 7BWJ)	Spike	100%	[55]
	N-terminal	Nucleocapsid	81-87%	[56]

	domain (PDB 7CR5)			
Anti-MERS-CoV antibody	Receptor binding domain (PDB 5GMQ)	Spike	70-81%	[57]
Anti-Influenza H1N1 antibody	Head domain (PDB 6XQ4)	Hemagglutinin	68-83%	[58]

Table S11. A summary of statistical significance evaluation using a one-way ANOVA with post hoc Holm-Sidak mean comparison test for serological selectivity of SARS-CoV-2 IgG-RBD

**Mean Comparisons**	**Blood p value**		**Plasma p value**		**Buffer p value**		**Sig.**[a]
	**100 pg. µl^{-1}**	**50 pg. µl^{-1}**	**100 pg. µl^{-1}**	**50 pg. µl^{-1}**	**100 pg. µl^{-1}**	**50 pg. µl^{-1}**	
HCoV-229E (IgG-N) SARS-CoV-2 (IgG-RBD)	6.58E-15	6.25E-15	2.55E-14	3.30E-15	6.81E-14	3.43E-13	1
MERS-CoV (IgG-N) SARS-CoV-2 (IgG-RBD)	6.90E-15	6.33E-15	2.85E-14	3.63E-15	7.06E-14	3.67E-13	1
Influenza A H1N1 (IgG-N) SARS-CoV-2 (IgG-RBD)	7.38E-15	6.51E-15	3.47E-14	3.85E-15	7.51E-14	3.99E-13	1
MERS-CoV (IgG-RBD) SARS-CoV-2 (IgG-RBD)	7.51E-15	6.88E-15	3.94E-14	4.04E-15	8.30E-14	5.06E-13	1
Influenza A H1N1 (IgG-RBD) SARS-CoV-2 (IgG-RBD)	9.09E-15	6.98E-15	3.95E-14	4.39E-15	9.89E-14	5.90E-13	1
SARS-CoV-2 (IgM-RBD) SARS-CoV-2 (IgG-RBD)	1.06E-14	1.94E-14	5.32E-14	6.75E-15	1.11E-13	6.92E-13	1
HCoV-229E (IgG-N) SARS-CoV-2 (IgM-RBD)	0.26238	0.01724	0.12903	0.09116	0.32972	0.21885	0
MERS-CoV (IgG-N)	0.31235	0.01826	0.1937	0.13861	0.36607	0.26549	0

SARS-CoV-2 (IgM-RBD)							
Influenza A H1N1 (IgG-N) SARS-CoV-2 (IgM-RBD)	0.39124	0.02095	0.34805	0.17898	0.43301	0.33389	0
MERS-CoV (IgG-RBD) SARS-CoV-2 (IgM-RBD)	0.41432	0.02725	0.352	0.21783	0.45325	0.33436	0
HCoV-229E (IgG-N) Influenza A H1N1 (IgG-RBD)	0.44137	0.02922	0.36776	0.2977	0.49796	0.39771	0
MERS-CoV (IgG-N) Influenza A H1N1 (IgG-RBD)	0.51213	0.78972	0.4815	0.47602	0.55969	0.48163	0
Influenza A H1N1 (IgG-N) Influenza A H1N1 (IgG-RBD)	0.6185	0.8119	0.48639	0.60871	0.57842	0.48737	0
MERS-CoV (IgG-RBD) Influenza A H1N1 (IgG-RBD)	0.64851	0.81726	0.50615	0.63247	0.68638	0.56102	0
Influenza A H1N1 (IgG-RBD) SARS-CoV-2 (IgM-RBD)	0.71294	0.83964	0.52601	0.69575	0.72481	0.58141	0
MERS-CoV (IgG-RBD) HCoV-229E (IgG-N)	0.74885	0.86583	0.53116	0.74395	0.74143	0.66919	0
HCoV-229E (IgG-	0.78125	0.89397	0.67052	0.78343	0.81506	0.77794	0

N) Influenza A H1N1 (IgG-N)							
MERS-CoV (IgG-N) MERS-CoV (IgG-RBD)	0.83913	0.92197	0.77711	0.81117	0.8369	0.77869	0
MERS-CoV (IgG-N) Influenza A H1N1 (IgG-N)	0.87256	0.94486	0.78326	0.83766	0.8424	0.7859	0
MERS-CoV (IgG-N) HCoV-229E (IgG-N)	0.9064	0.97151	0.80788	0.87845	0.90086	0.87589	0
$F_{6,14}$ values	327.5460	329.3973	263.5958	359.1365	232.9898	181.5124	

[a] Significant p values are denoted by a one (1) and non-significant p values are denoted by a zero (0).

Table S12. A summary of statistical significance evaluation using a one-way ANOVA with post hoc Holm-Sidak mean comparison test for serological selectivity of SARS-CoV-2 IgM-RBD

**Mean Comparisons**	**Blood p value**		**Plasma p value**		**Buffer p value**		**Sig.[a]**
	**100 pg. µl^{-1}**	**50 pg. µl^{-1}**	**100 pg. µl^{-1}**	**50 pg. µl^{-1}**	**100 pg. µl^{-1}**	**50 pg. µl^{-1}**	
HCoV-229E (IgG-N) SARS-CoV-2 (IgM-RBD)	2.27E-12	1.68E-12	3.60E-13	4.12E-12	4.04E-11	1.09E-13	1
SARS-CoV-2 (IgM-RBD) SARS-CoV-2 (IgG-RBD)	2.70E-12	2.23E-12	3.61E-13	5.59E-12	4.06E-11	1.12E-13	1
Influenza A H1N1 (IgM-N) SARS-CoV-2 (IgM-RBD)	3.09E-12	2.72E-12	3.65E-13	5.70E-12	4.57E-11	1.20E-13	1
Influenza A H1N1	3.74E-12	3.01E-12	4.03E-13	5.89E-12	4.72E-11	1.21E-13	1

(IgM-RBD) SARS-CoV-2 (IgM-RBD)							
MERS-CoV (IgM-RBD) SARS-CoV-2 (IgM-RBD)	3.82E-12	3.42E-12	4.57E-13	8.06E-12	5.77E-11	1.21E-13	1
MERS-CoV (IgM-RBD) HCoV-229E (IgG-N)	0.2437	0.11387	0.52198	0.16223	0.51845	0.74855	0
HCoV-229E (IgG-N) Influenza A H1N1 (IgM-RBD)	0.26263	0.18509	0.52751	0.43708	0.52297	0.75508	0
MERS-CoV (IgM-RBD) SARS-CoV-2 (IgG-RBD)	0.43195	0.26597	0.54373	0.43877	0.67162	0.77592	0
Influenza A H1N1 (IgM-RBD) SARS-CoV-2 (IgG-RBD)	0.46046	0.33243	0.73458	0.46137	0.7158	0.81735	0
HCoV-229E (IgG-N) Influenza A H1N1 (IgM-N)	0.47905	0.49084	0.75998	0.48041	0.77491	0.82406	0
MERS-CoV (IgM-RBD) Influenza A H1N1 (IgM-N)	0.62924	0.50013	0.76659	0.50431	0.78036	0.84544	0
Influenza A H1N1 (IgM-N) Influenza A H1N1 (IgM-RBD)	0.66438	0.60008	0.78583	0.50615	0.82139	0.92827	0
HCoV-229E (IgG-N) SARS-CoV-2 (IgG-RBD)	0.687	0.64575	0.97291	0.90993	0.82694	0.97118	0
Influenza A H1N1 (IgM-N)	0.75614	0.76985	0.97986	0.94332	0.9518	0.97808	0

SARS-CoV-2 (IgG-RBD) MERS-CoV (IgM-RBD) Influenza A H1N1 (IgM-RBD)	0.96045	0.81497	0.99304	0.96646	0.99429	0.9931	0
$F_{5,12}$ values	255.7395	264.3684	361.9643	230.0317	161.3457	442.9206	

[a] Significant p values are denoted by a one (1) and non-significant p values are denoted by a zero (0).

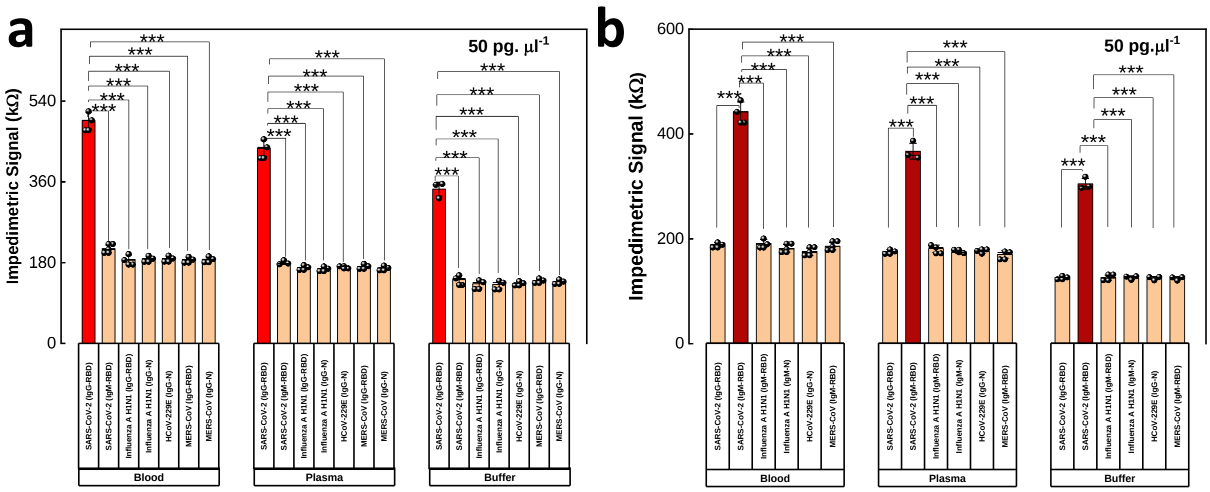

Figure S22. Selectivity of over the linear range of antibody detection in blood, plasma, and buffer for (a) IgG-RBD and (b) IgM-RBD at 50 pg. μl^{-1}. Similar cross reactivity was studied for the NFluidEX IgG-RBD and IgM-RBD assay over the linear range from 10 pg. μl^{-1} – 10^4 pg. μl^{-1} against antibodies from similar viruses.

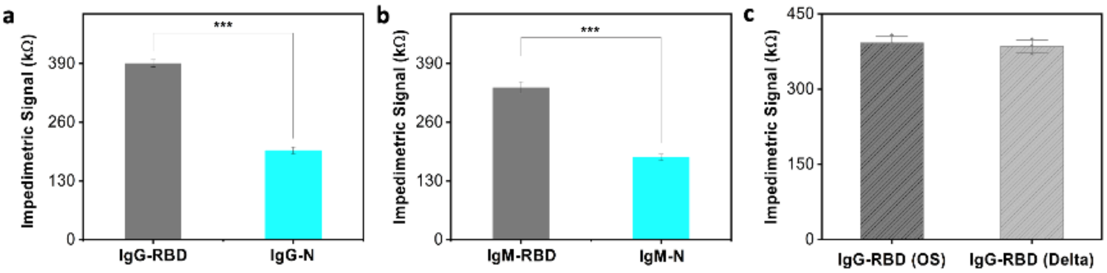

Figure S23. Cross reactivity study for (a) IgG-N on the IgG-RBD imprinted assay ($F_{1,4}$ = 305.487, p = 6.29139E-5), (b) IgM-N on the IgM-RBD imprinted assay ($F_{1,4}$ = 119.298, p = 3.99023E-4), (c) IgG-RBD belonging to the Delta variant on the IgG-RBD original strain imprinted assay showing non-significant response due to similarity of the binding affinities between antigens and antibodies ($F_{1,4}$ = 0.28315, p = 0.62283); at 100 pg. μl^{-1}, *** $p < .001$.

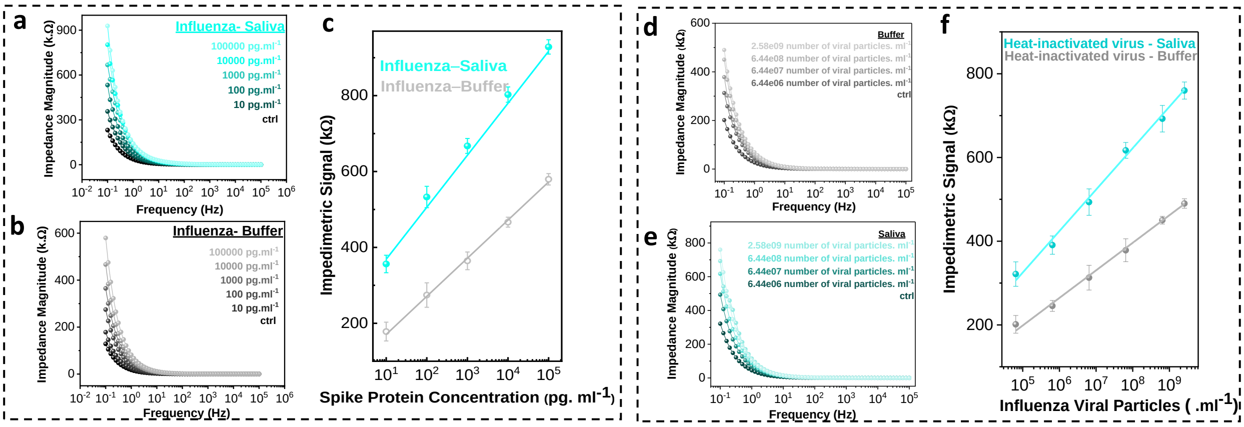

Figure S24. Calibration plots for the Influenza A NMI/MIP assay. Bode response showing the impedance magnitude for the detection of Influenza SP in (a) buffer and (b) saliva, and (c) corresponding linear calibration plot for Influenza SP. Bode response showing the impedance magnitude in for the detection of heat-inactivated Influenza viral particles in (d) buffer and (e) saliva, and (f) corresponding linear calibration plot for heat-inactivated Influenza viral particles. The NMI/MIP assay was fabricated with the same protocol as for the SARS-CoV-2 SP; linear range from 10 pg. ml^{-1} – 10^5 pg. ml^{-1} for SP detection and from 6.44e6 – 2.58e9 number of viral particles. ml^{-1} for viral particles.

Surface plasmon resonance (SPR) to study affinity

The SPR results shown in **Figure S25** were fit with a Hill function (**Equation S3**):[59]

$$y = V_{\max} \frac{x^n}{K_d^n + x^n} \qquad \textbf{Equation S3}$$

where V_{max} is the velocity for maximum specific binding, x is the concentration of analyte, and n is the Hill coefficient, which represents the degree of cooperativity for the binding of x to the target. K_d is dissociation constant.

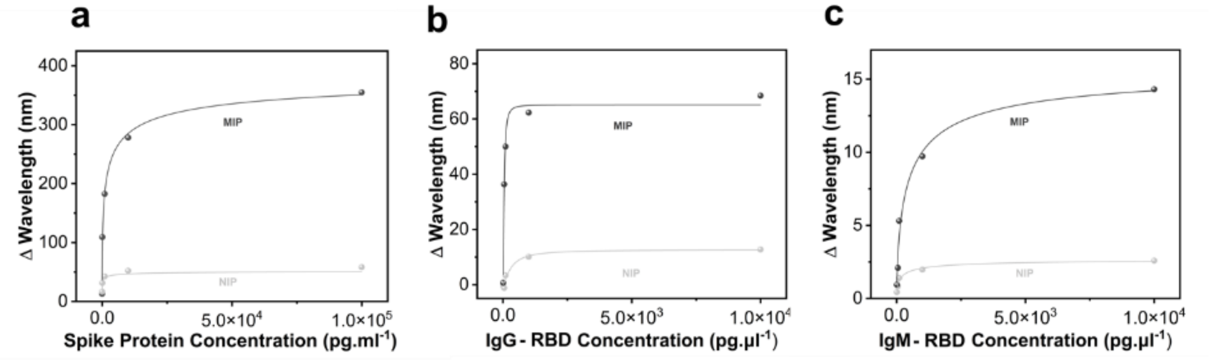

Figure S25. **Affinity study of the NMI/MIP and NMI/NIP assays via SPR, showing Δ wavelength vs target.** Optical shift in wavelength denoted for the binding of the SARS-CoV-2 (a) Spike protein, (b) IgG-RBD, and (c) IgM-RBD at different concentrations to the NMI/MIP and NMI/NIP test assay obtained in 1X PBS buffer with portable SPR equipment, and the fitting of the data with Hill function.

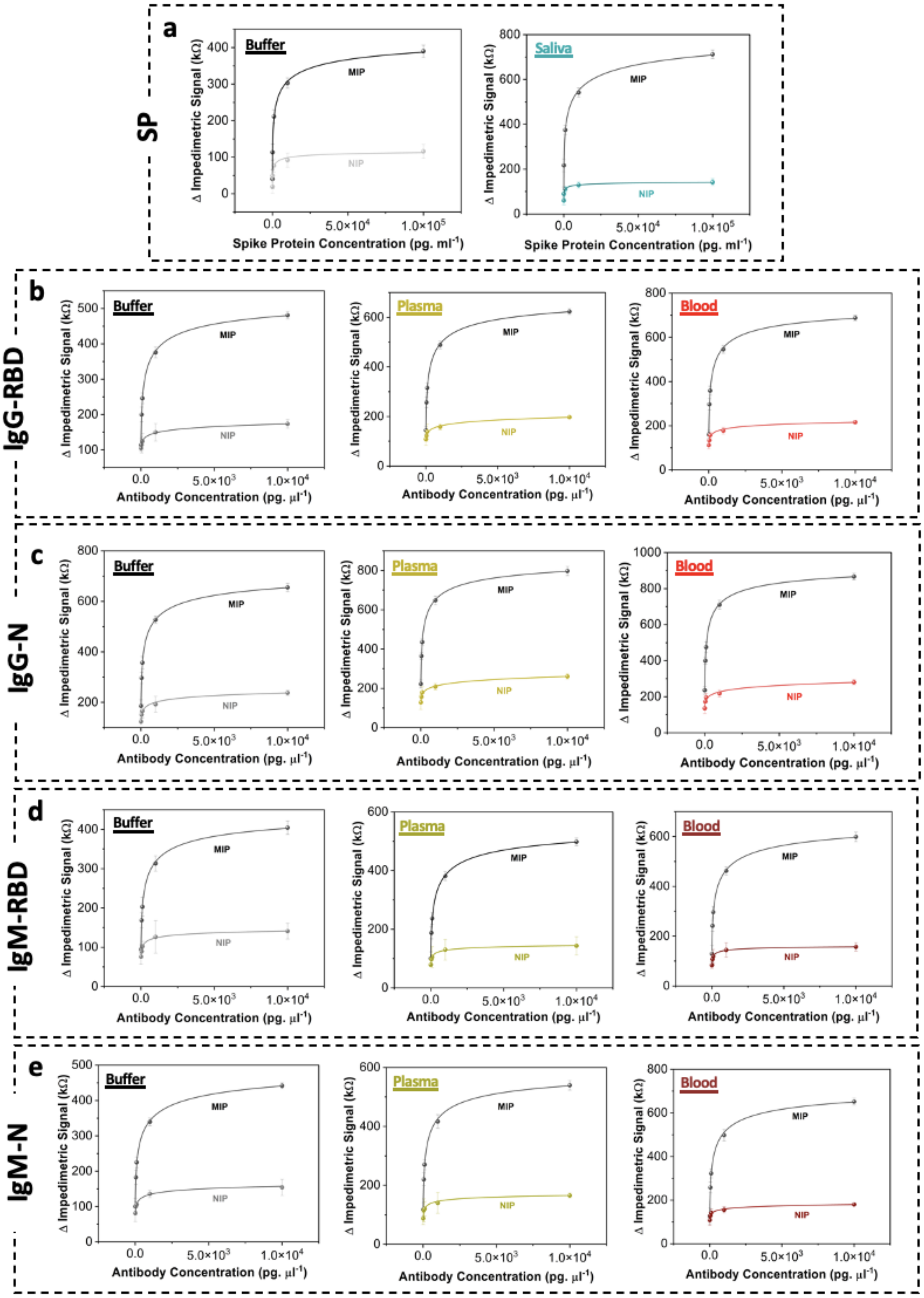

Figure S26. NMI/MIP and NMI/NIP for the detection of SARS-CoV-2 SP and antibodies. NMI/NIP and NMI/MIP responses for the detection of SARS-CoV-2 (a) SP in buffer and saliva, (b-e) IgG-RBD, IgG-N, IgM-RBD and IgM-N, in buffer, undiluted plasma and whole blood. results are fitted using the Hill equation. Data shows the mean ± standard deviation over triplicates.

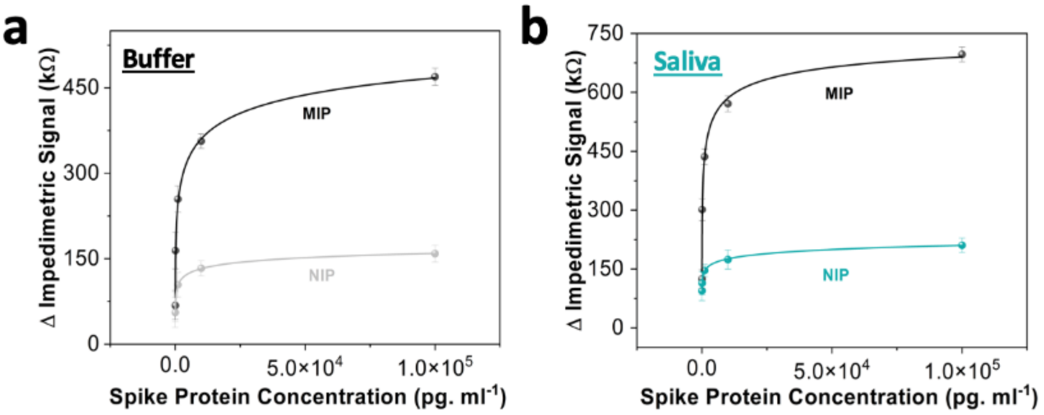

Figure S27. NMI/MIP and NMI/NIP for the detection of Influenza viral protein. NMI/NIP and NMI/MIP responses for the detection of Influenza A viral protein in (a) buffer and (b) saliva. Results are fitted using the Hill equation. Data shows the mean ± standard deviation over triplicates.

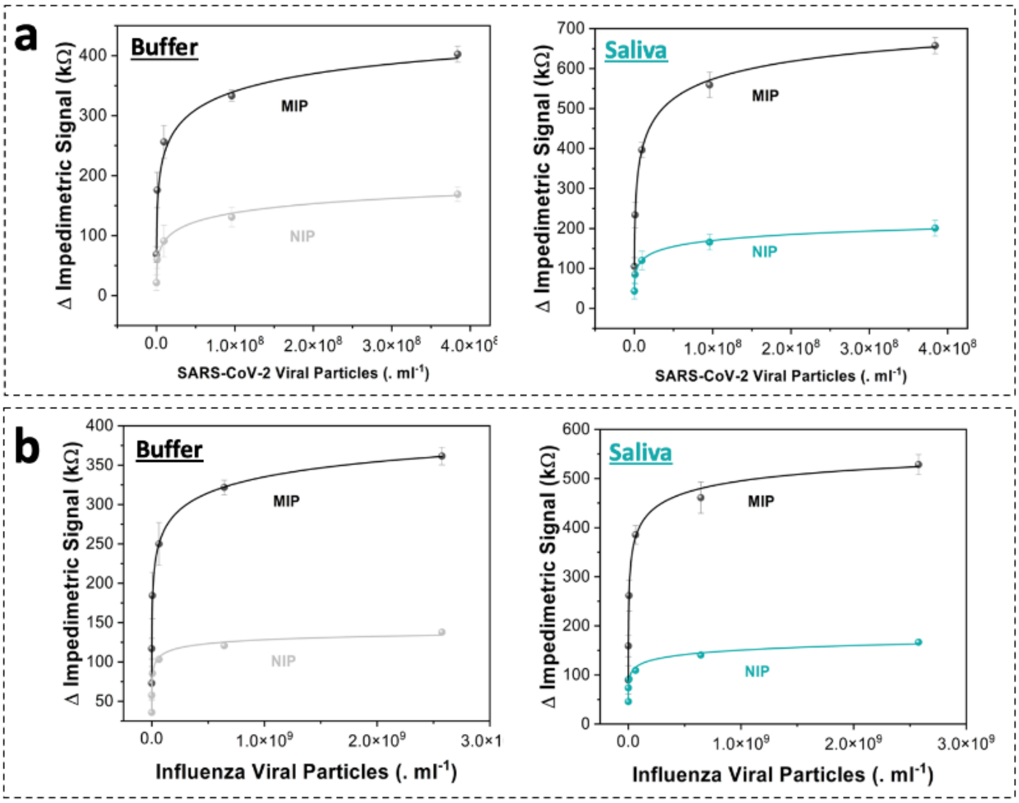

Figure S28. NMI/MIP and NMI/NIP for the detection of the whole viral particles. NMI/NIP and NMI/MIP responses for the detection of heat-inactivated whole viral particle of (a) SARS-CoV-2 and (b) Influenza A in buffer and saliva. Results are fitted using the Hill equation. Data shows the mean ± standard deviation over triplicates.

Table S13. SARS-CoV-2 dissociation constants on the NMI/MIP test assay

**Analyte**	**Media**	**ΔZ_{max}**	**Hill coefficient (n)**	**K_d via electrochemistry**	**K_d via electrochemistry**	**K_d via SPR**
SP	Buffer	123.75 kΩ	0.41	358.16 pg. ml^{-1}	0.82 pM	3.17 pM
SP	Saliva	921.74 kΩ	0.35	3227.25 pg. ml^{-1}	7.36 pM	
IgG-RBD	Buffer	563.03 kΩ	0.44	193.33 pg. µl^{-1}	1.32 nM	0.32 nM
IgG-RBD	Plasma	723.99 kΩ	0.46	191.55 pg. µl^{-1}	1.31 nM	
IgG-RBD	Blood	781.56 kΩ	0.47	154.11 pg. µl^{-1}	1.05 nM	
IgG-N	Buffer	768.22 kΩ	0.41	150.05 pg. µl^{-1}	1.03 nM	
IgG-N	Plasma	723.99 kΩ	0.46	191.55 pg. µl^{-1}	1.31 nM	
IgG-N	Blood	973.83 kΩ	0.46	112.46 pg. µl^{-1}	0.77 nM	
IgM-RBD	Buffer	472.10 kΩ	0.45	201.35 pg. µl^{-1}	0.25 nM	0.57 nM
IgM-RBD	Plasma	587.19 kΩ	0.47	254.64 pg. µl^{-1}	0.31 nM	
IgM-RBD	Blood	710.39 kΩ	0.44	232.29 pg. µl^{-1}	0.29 nM	
IgM-N	Buffer	537.93 kΩ	0.41	256.08 pg. µl^{-1}	0.32 nM	
IgM-N	Plasma	624.34 kΩ	0.46	193.15 pg. µl^{-1}	0.24 nM	
IgM-N	Blood	758.49 kΩ	0.47	212.19 pg. µl^{-1}	0.26 nM	
Viral particle	Buffer	558.93 kΩ	0.33	2.57E+07 particles. ml^{-1}	--	
Viral particle	Saliva	823.45 kΩ	0.39	1.18E+07 particles. ml^{-1}	--	

Table S14. Influenza A dissociation constants on the NMI/MIP test assay

**Analyte**	**Media**	**ΔZ_{max}**	**Hill coefficient (n)**	**K_d via electrochemistry**	**K_d via electrochemistry**
SP	Buffer	652.52 kΩ	0.31	5091.18 pg. ml^{-1}	30.77 pM
SP	Saliva	799.10kΩ	0.36	628.59 pg. ml^{-1}	3.80 pM
Viral particle	Buffer	504.77 kΩ	0.25	6.90E+07 particles. ml^{-1}	--
Viral particle	Saliva	625.20 kΩ	0.33	1.66E+07 particles. ml^{-1}	--

Table S15. Comparison of current K_d values for SARS-CoV-2 viral entities

**Target**	**K_d**	**Method**	**Ref**
Wild type SP	120 pM	Dot blot (colorimetric)	[27]
Alpha SP	290 pM	Dot blot (colorimetric)	[27]
Delta SP	480 pM	Dot blot (colorimetric)	[27]
Wild type virus	2.1 pM	Dot blot (colorimetric)	[27]
Alpha virus	2.3 pM	Dot blot (colorimetric)	[27]
Wild type SP	83 pM	Dynamic light scattering of polyclonal	[60]

		antibody-functionalized spherical gold NP biosensor	
Wild type IgG-S	0.87 fM	Graphene field-effect transistor modified with S1 protein	[61]
Wild type IgG-S	9.55 pM	LSPR	[62]
Wild type IgM-S	3.33 x 10^{-7} M	LSPR	[62]
Wild type SP	1.42 pM	LSPR	[62]
Wild type SP	7 nM	SPR	[63]
Wild type SP	2.7 pg mL^{-1}	Electrochemistry	[64]
Wild type SP	1.6 - 60 nM (depending on the media)	SPR	[65]
**Whole viral particle**	**Table S13**	**Electrochemistry**	**This work**
**Wild type SP**	**Table S13**	**SPR & Electrochemistry**	
**IgG-RBD**	**Table S13**	**SPR & Electrochemistry**	
**IgM-RBD**	**Table S13**	**SPR & Electrochemistry**	

Table S16. Comparison of current K_d values for Influenza H1N1 viral entities

**Target**	**K_d**	**Method**	**Ref**
Influenza SP	19.2 nM	DPV	[66]
Influenza particle	5.56 ± 4.41 nM	Sandwich ELONA	[67]
Influenza SP	1.9-8.7 × 10^{-11} M	SPR	[68]
Influenza SP	1.53-2.47 × 10^{-8} M	SPR	[69]
Influenza SP	0.64 uM	Bio-layer interferometry	[70]
**Whole viral particle**	**Table S14**	**Electrochemistry**	**This work**
**Wild type SP**	**Table S14**	**Electrochemistry**	

Imprinting factor

$$IF = \frac{\langle \Delta Z_{MIP} \rangle}{\langle \Delta Z_{NIP} \rangle} \quad \textbf{Equation S4}$$

Table S17. Imprinting factor (IF) for SARS-CoV-2 viral entities on the NMI/MIP test assay

Analyte	Media	Concentration	<ΔZ_{MIP}> (kΩ) (n=3)	<ΔZ_{NIP}> (kΩ) (n=3)	IF
SP	Buffer	10 pg. ml^{-1}	40.21	19.55	2.06
		100 pg. ml^{-1}	113.46	49.04	2.31
		1000 pg. ml^{-1}	211.30	77.12	2.74
		10000 pg. ml^{-1}	302.85	91.93	3.29
		100000 pg. ml^{-1}	390.02	115.99	3.36
	Saliva	10 pg. ml^{-1}	89.80	59.77	1.50
		100 pg. ml^{-1}	216.68	89.65	2.42
		1000 pg. ml^{-1}	374.68	110.65	3.39
		10000 pg. ml^{-1}	541.95	128.92	4.20
		100000 pg. ml^{-1}	711.83	141.80	5.02
IgG-RBD	Buffer	10 pg. µl^{-1}	114.14	104.98	1.09
		50 pg. µl^{-1}	199.88	114.59	1.74
		100 pg. µl^{-1}	245.80	124.36	1.98
		1000 pg. µl^{-1}	375.44	149.94	2.50
		10000 pg. µl^{-1}	480.73	173.59	2.77
	Plasma	10 pg. µl^{-1}	144.18	108.08	1.33
		50 pg. µl^{-1}	256.64	122.05	2.10
		100 pg. µl^{-1}	315.20	138.50	2.28
		1000 pg. µl^{-1}	488.54	157.96	3.09
		10000 pg. µl^{-1}	623.36	197.17	3.16
	Blood	10 pg. µl^{-1}	161.05	112.07	1.44
		50 pg. µl^{-1}	296.97	134.07	2.22
		100 pg. µl^{-1}	358.68	157.16	2.28
		1000 pg. µl^{-1}	545.56	177.49	3.07
		10000 pg. µl^{-1}	687.64	216.04	3.18
IgG-N	Buffer	10 pg. µl^{-1}	185.92	124.22	1.50
		50 pg. µl^{-1}	296.84	151.63	1.96
		100 pg. µl^{-1}	356.90	165.96	2.15
		1000 pg. µl^{-1}	526.92	192.46	2.74
		10000 pg. µl^{-1}	654.92	237.65	2.76
	Plasma	10 pg. µl^{-1}	222.54	128.67	1.73
		50 pg. µl^{-1}	364.49	157.80	2.31
		100 pg. µl^{-1}	435.46	179.74	2.42
		1000 pg. µl^{-1}	647.85	208.55	3.11
		10000 pg. µl^{-1}	797.16	260.86	3.06
	Blood	10 pg. µl^{-1}	236.01	135.19	1.75
		50 pg. µl^{-1}	399.72	173.36	2.31
		100 pg. µl^{-1}	474.88	195.66	2.43
		1000 pg. µl^{-1}	710.07	218.00	3.26
		10000 pg. µl^{-1}	866.62	280.36	3.09
IgM-RBD	Buffer	10 pg. µl^{-1}	95.07	76.06	1.25
		50 pg. µl^{-1}	168.50	89.39	1.88

		100 pg. µl^{-1}	202.93	102.10	1.99
		1000 pg. µl^{-1}	313.20	126.13	2.48
		10000 pg. µl^{-1}	404.11	140.88	2.87
		10 pg. µl^{-1}	100.76	78.92	1.28
		50 pg. µl^{-1}	187.70	97.65	1.92
	Plasma	100 pg. µl^{-1}	236.87	105.02	2.26
		1000 pg. µl^{-1}	381.17	130.19	2.93
		10000 pg. µl^{-1}	497.85	143.59	3.47
		10 pg. µl^{-1}	129.42	83.68	1.55
		50 pg. µl^{-1}	241.59	107.33	2.25
	Blood	100 pg. µl^{-1}	296.38	118.64	2.50
		1000 pg. µl^{-1}	462.18	144.49	3.20
		10000 pg. µl^{-1}	598.66	156.92	3.81
		10 pg. µl^{-1}	100.32	81.18	1.24
		50 pg. µl^{-1}	182.54	100.38	1.82
	Buffer	100 pg. µl^{-1}	225.06	106.05	2.12
		1000 pg. µl^{-1}	339.52	136.05	2.50
		10000 pg. µl^{-1}	441.47	154.13	2.86
		10 pg. µl^{-1}	117.86	87.91	1.34
		50 pg. µl^{-1}	220.01	115.13	1.91
IgM-N	Plasma	100 pg. µl^{-1}	270.25	120.18	2.25
		1000 pg. µl^{-1}	416.61	140.35	2.97
		10000 pg. µl^{-1}	539.32	165.46	3.26
		10 pg. µl^{-1}	126.74	108.50	1.17
		50 pg. µl^{-1}	257.76	128.87	2.00
	Blood	100 pg. µl^{-1}	322.38	141.98	2.27
		1000 pg. µl^{-1}	498.01	155.02	3.21
		10000 pg. µl^{-1}	652.01	180.06	3.62
		96000 particles. ml^{-1}	68.75	21.62	3.18
		960000 particles. ml^{-1}	175.86	60.14	2.92
	Buffer	9600000 particles. ml^{-1}	256.30	90.97	2.82
		9.60E+07 particles. ml^{-1}	333.09	130.91	2.54
Viral particles		3.84E+08 particles. ml^{-1}	402.39	168.99	2.38
		96000 particles. ml^{-1}	105.20	43.57	2.41
		960000 particles. ml^{-1}	234.00	85.08	2.75
	Saliva	9600000 particles. ml^{-1}	396.53	120.16	3.30
		9.60E+07 particles. ml^{-1}	559.33	165.82	3.37
		3.84E+08 particles. ml^{-1}	657.33	200.88	3.27

Table S18. Imprinting factor (IF) for Influenza A viral entities on the NMI/MIP test assay

**Analyte**	**Media**	**Concentration**	**$\langle \Delta Z_{MIP} \rangle$ (kΩ) (n=3)**	**$\langle \Delta Z_{NIP} \rangle$ (kΩ) (n=3)**	**IF**
		10 pg. ml^{-1}	68.05	55.73	1.22
		100 pg. ml^{-1}	164.31	67.65	2.43
	Buffer	1000 pg. ml^{-1}	254.53	104.32	2.44
		10000 pg. ml^{-1}	356.34	133.29	2.68
SP		100000 pg. ml^{-1}	469.35	158.98	2.95
		10 pg. ml^{-1}	124.66	94.57	1.32
	Saliva	100 pg. ml^{-1}	301.28	113.86	2.65
		1000 pg. ml^{-1}	435.83	145.97	2.99
		10000 pg. ml^{-1}	571.15	173.92	3.28

		100000 pg. ml^{-1}	696.77	210.45	3.31
Viral particles	Buffer	64400 particles. ml^{-1}	72.94	35.93	2.03
		644000 particles. ml^{-1}	116.93	57.76	2.02
		6440000 particles. ml^{-1}	184.40	85.55	2.16
		6.44E+07 particles. ml^{-1}	249.87	103.37	2.42
		6.44E+08 particles. ml^{-1}	321.75	120.72	2.67
		2.58E+09 particles. ml^{-1}	361.61	137.94	2.62
	Saliva	64400 particles. ml^{-1}	89.87	45.93	1.96
		644000 particles. ml^{-1}	159.04	73.68	2.16
		6440000 particles. ml^{-1}	261.79	91.48	2.86
		6.44E+07 particles. ml^{-1}	385.33	109.30	3.53
		6.44E+08 particles. ml^{-1}	460.96	140.64	3.28
		2.58E+09 particles. ml^{-1}	528.25	166.84	3.17

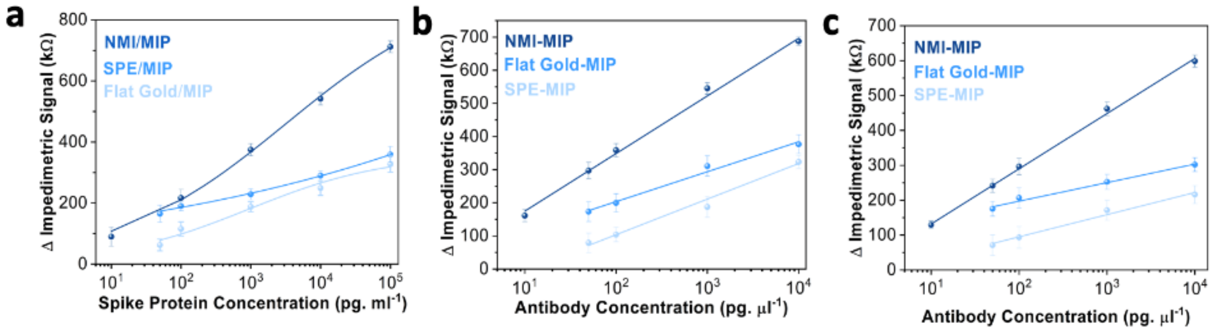

Figure S29. Comparison between the NMI/MIP, SPE/MIP and flat gold/MIP responses for (a) SP in saliva, (b) IgG-RBD in whole blood, and (c) IgM-RBD in whole blood.

Table S19. Limit of Detection (LOD) for SP, IgG-RBD and IgM-RBD over different MIP assays

Analyte	Media	Assay	LOD	Linear Range	Sensitivity
Spike Protein	Saliva	NMI/MIP	5.89 pg. ml^{-1}	1.00E+01 - 1.00E+05 pg. ml^{-1}	68.16 kΩ/ pg. ml^{-1}
		SPE/MIP	26.96 pg. ml^{-1}	5.00E+01 - 1.00E+05 pg. ml^{-1}	24.59 kΩ/ pg. ml^{-1}
		flat gold/MIP	45.94 pg. ml^{-1}	5.00E+01 - 1.00E+05 pg. ml^{-1}	32.92 kΩ/ pg. ml^{-1}
IgG-RBD	Blood	NMI/MIP	5.74 pg. µl^{-1}	1.00E+01 - 1.00E+04 pg. µl^{-1}	76.71 kΩ/ pg. µl^{-1}
		SPE/MIP	34.51 pg. µl^{-1}	5.00E+01 - 1.00E+04 pg. µl^{-1}	39.38 kΩ/ pg. µl^{-1}
		flat gold/MIP	49.61 pg. µl^{-1}	5.00E+01 - 1.00E+04 pg. µl^{-1}	45.44 kΩ/ pg. µl^{-1}
IgM-RBD	Blood	NMI/MIP	3.13 pg. µl^{-1}	1.00E+01 - 1.00E+04 pg. µl^{-1}	68.54 kΩ/ pg. µl^{-1}
		SPE/MIP	33.96 pg. µl^{-1}	5.00E+01 - 1.00E+04 pg. µl^{-1}	22.79 kΩ/ pg. µl^{-1}
		flat gold/MIP	47.50 pg. µl^{-1}	5.00E+01 - 1.00E+04 pg. µl^{-1}	27.86 kΩ/ pg. µl^{-1}

3. Patient Sample Validation and a Field Study

Raw Impedance Response of Patient Samples

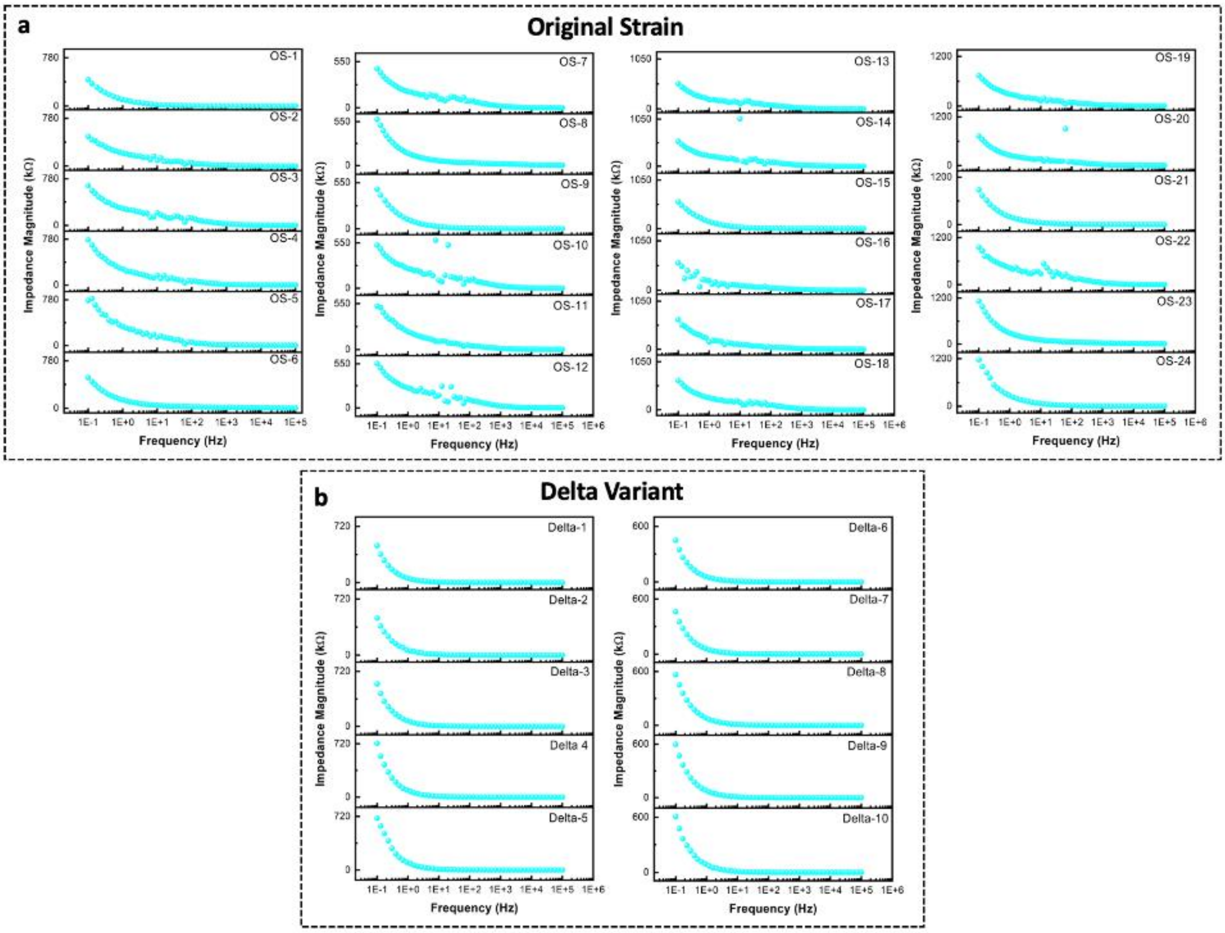

Figure S30. Bode plots of raw impedance response detecting whole viral particles from patient saliva samples diagnosed with (a) the original strain of SARS-CoV-2 and (b) Delta variant of SARS-CoV-2. Variant infections were verified and reported using real-time quantitative polymerase chain reaction (RT-qPCR).

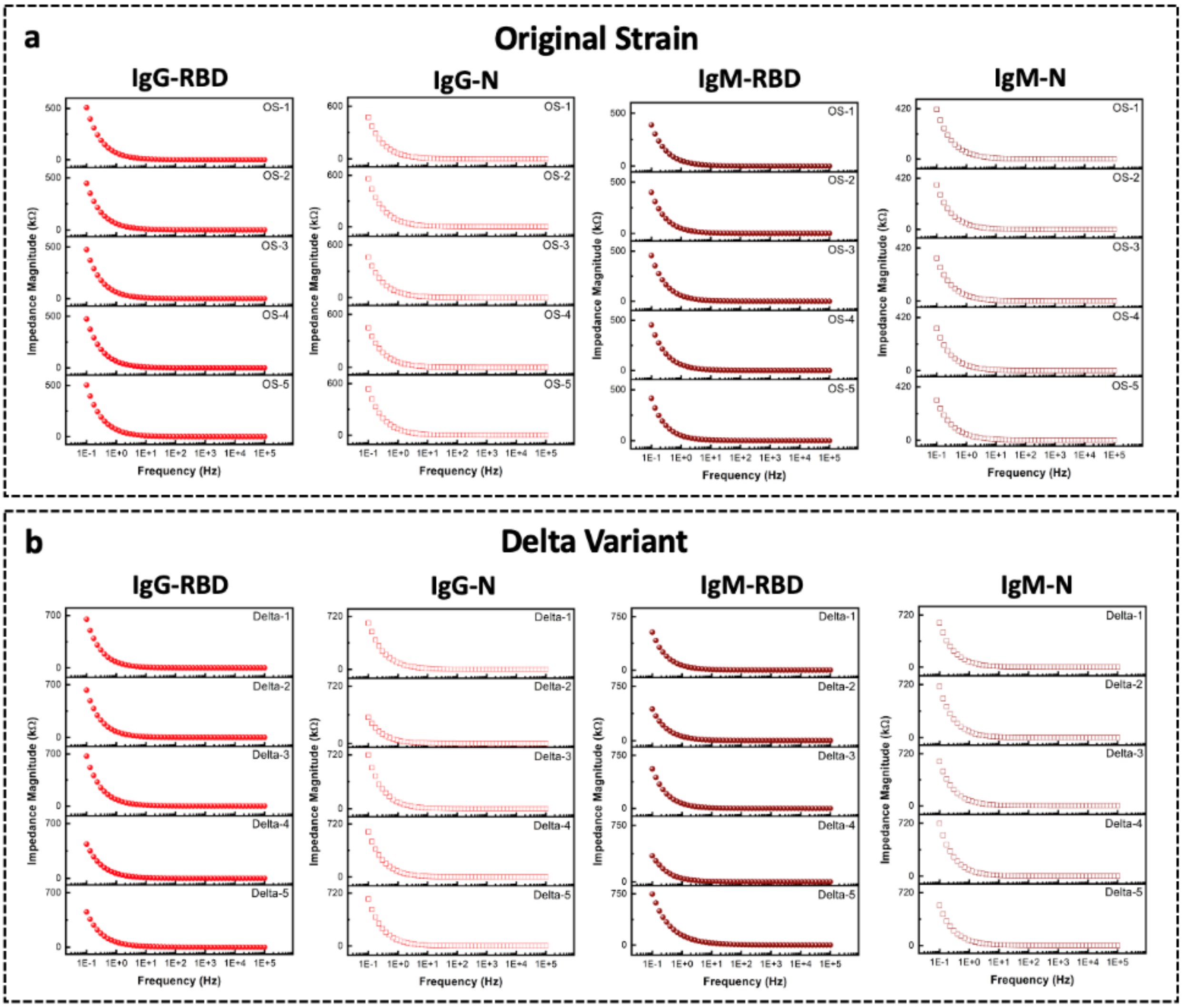

Figure S31. Bode plots of raw impedance response detecting IgG-RBD, IgG-N, IgM-RBD and IgM-N from patient whole blood samples diagnosed with (a) the original strain of SARS-CoV-2 and (b) Delta variant of SARS-CoV-2.

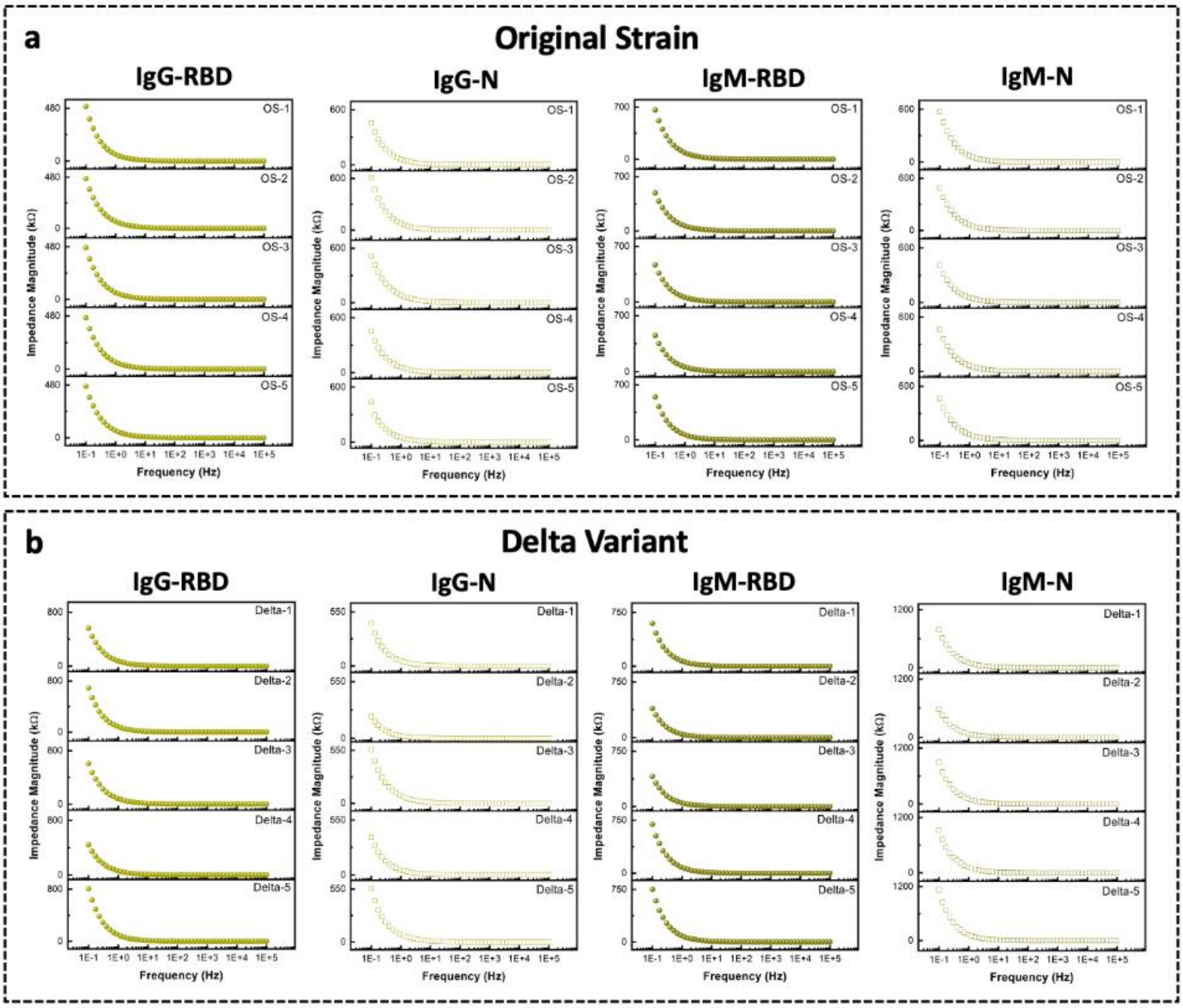

Figure S32. Bode plots of raw impedance response detecting IgG-RBD, IgG-N, IgM-RBD and IgM-N from patient undiluted plasma samples diagnosed with (a) the original strain of SARS-CoV-2 and (b) Delta variant of SARS-CoV-2.

RT-qPCR Calibration Curve for Assessment of quantitative NFluidEX

To quantify the results obtained by the NFluidEX in comparison with the RT-qPCR gold standard method, we used the cycle threshold (Ct) values using the RNA-dependent RNA polymerase (RdRp) gene for amplification in RT-qPCR, to relate the concentration of SARS-CoV-2 viral particles with the RT-qPCR response. **Figure S33** shows the calibration plot that relates the Ct values of RT-qPCR with viral particle concentration, which is consistent with the known trend of a decreasing Ct for increasing viral load.[41,71–74]

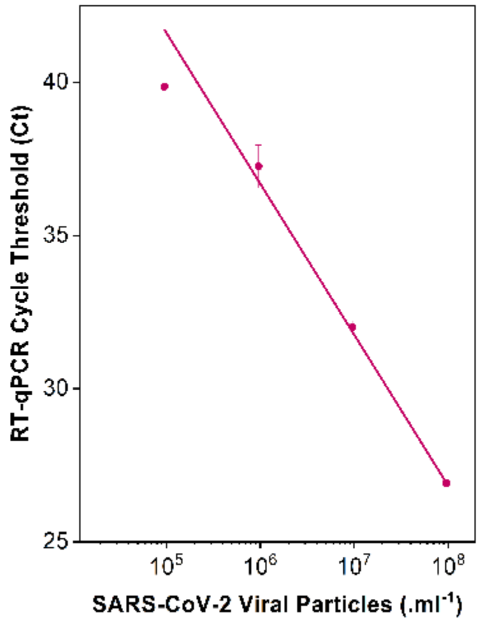

Figure S33. Calibration plot of RT-qPCR Ct values as a function of heat-inactivated SARS-CoV-2 viral particle concentration.

Table S20. RT-qPCR Ct values for patient samples

Patient Code	Ct value
OS-1	31.92
OS-2	32.85
OS-3	27.21
OS-4	24.36
OS-5	25.20
OS-6	28.13
OS-7	37.64
OS-8	22.88
OS-9	35.59
OS-10	34.45
OS-11	27.58
OS-13	29.28
OS-14	30.85
OS-17	30.61
OS-22	22.48

OS-24	16.19
Delta-1	35.01
Delta-2	29.48
Delta-3	25.27
Delta-4	27.15
Delta-5	28.24
Delta-6	25.23
Delta-7	27.78
Delta-8	27.59
Delta-9	39.24
Delta-10	35.31

Table S21. ELISA optical density (OD) values for patient samples

Patient Code	Antibodies	$OD_{450\ nm}$ value
OS-1	IgG	11.19
	IgM	5.04
OS-2	IgG	0.10
	IgM	2.35
OS-3	IgG	5.02
	IgM	1.02
OS-4	IgG	1.11
	IgM	0.10
OS-5	IgG	10.89
	IgM	3.23

References:

[1] D. M. Jenkins, B. E. Lee, S. Jun, J. Reyes-De-Corcuera, E. S. McLamore, *J. Electrochem. Soc.* **2019**, *166*, B3056.

[2] K. L. Helton, K. E. Nelson, E. Fu, P. Yager, *Lab Chip* **2008**, *8*, 1847.

[3] B. Johannsen, L. Müller, D. Baumgartner, L. Karkossa, S. M. Früh, N. Bostanci, M. Karpíšek, R. Zengerle, N. Paust, K. Mitsakakis, *Micromachines* **2019**, *10*, DOI 10.3390/mi10120833.

[4] K. Lee, T. Yoon, H. S. Yang, S. Cha, Y. P. Cheon, L. Kashefi-Kheyrabadi, H. Il Jung, *Lab Chip* **2020**, *20*, 320.

[5] H. De Puig, R. A. Lee, D. Najjar, X. Tan, L. R. Soekensen, N. M. Angenent-Mari, N. M. Donghia, N. E. Weckman, A. Ory, C. F. Ng, P. Q. Nguyen, A. S. Mao, T. C. Ferrante, G. Lansberry, H. Sallum, J. Niemi, J. J. Collins, *Sci. Adv.* **2021**, *7*, DOI 10.1126/sciadv.abh2944.

[6] C. Del Real Mata, R. Siavash Moakhar, I. I. Hosseini, M. Jalali, S. Mahshid, *Nanoscale* **2021**, *13*, 14316.

[7] R. Siavash Moakhar, T. Abdelfatah, A. Sanati, M. Jalali, S. E. Flynn, S. S. Mahshid, S. Mahshid, *ACS Appl. Mater. Interfaces* **2020**, *12*, 23298.

[8] M. Jalali, T. AbdelFatah, S. S. Mahshid, M. Labib, A. Sudalaiyadum Perumal, S. Mahshid, *Small* **2018**, *14*, 1801893.

[9] T. AbdelFatah, M. Jalali, S. Mahshid, *Biomicrofluidics* **2018**, *12*, 064103(1).

[10] K. Iwai, K. C. Shih, X. Lin, T. A. Brubaker, R. D. Sochol, L. Lin, *Lab Chip* **2014**, *14*, 3790.

[11] E. Kubala, P. Strzelecka, M. Grzegocka, D. Lietz-Kijak, H. Gronwald, P. Skomro, E. Kijak, *Biomed Res. Int.* **2018**, *2018*, DOI 10.1155/2018/6572381.

[12] R. P. Kusy, D. L. Schafer, *J. Mater. Sci. Mater. Med.* **1995**, *6*, 385.

[13] D. J. Vitello, R. M. Ripper, M. R. Fettiplace, G. L. Weinberg, J. M. Vitello, *J. Vet. Med.* **2015**, *2015*, 1.

[14] M. Jalali, I. Isaac Hosseini, T. AbdelFatah, L. Montermini, S. Wachsmann Hogiu, J. Rak, S. Mahshid, *Lab Chip* **2021**, *21*, 855.

[15] N. S. Mazlan, M. M. Ramli, M. M. A. B. Abdullah, D. S. C. Halin, S. S. M. Isa, L. F. A. Talip, N. S. Danial, S. A. Z. Murad, in *AIP Conf. Proc.*, AIP Publishing LLC AIP Publishing, **2017**, p. 020276.

[16] A. Sanati, R. Siavash Moakhar, I. I. Hosseini, K. Raeissi, F. Karimzadeh, M. Jalali, M. Kharaziha, S. Sheibani, L. Shariati, J. F. Presley, H. Vali, S. Mahshid, *ACS Sensors*

2021, *6*, 797.

[17] M. Jalali, T. AbdelFatah, S. S. Mahshid, M. Labib, S. Mahshid, *Small* **2018**, *1801893*, 1.

[18] L. Fabiani, M. Saroglia, G. Galatà, R. De Santis, S. Fillo, V. Luca, G. Faggioni, N. D'Amore, E. Regalbuto, P. Salvatori, G. Terova, D. Moscone, F. Lista, F. Arduini, *Biosens. Bioelectron.* **2021**, *171*, DOI 10.1016/j.bios.2020.112686.

[19] A. Yakoh, U. Pimpitak, S. Rengpipat, N. Hirankarn, O. Chailapakul, S. Chaiyo, *Biosens. Bioelectron.* **2021**, *176*, 112912.

[20] S. A. Hashemi, N. G. Golab Behbahan, S. Bahrani, S. M. Mousavi, A. Gholami, S. Ramakrishna, M. Firoozsani, M. Moghadami, K. B. Lankarani, N. Omidifar, *Biosens. Bioelectron.* **2021**, *171*, 112731.

[21] H. Yousefi, A. Mahmud, D. Chang, J. Das, S. Gomis, J. B. Chen, H. Wang, T. Been, L. Yip, E. Coomes, Z. Li, S. Mubareka, A. Mcgeer, N. Christie, S. Gray-Owen, A. Cochrane, J. M. Rini, E. H. Sargent, S. O. Kelley, *J. Am. Chem. Soc.* **2021**, *143*, 1722.

[22] R. Peng, Y. Pan, Z. Li, Z. Qin, J. M. Rini, X. Liu, *Biosens. Bioelectron.* **2022**, *197*, DOI 10.1016/j.bios.2021.113762.

[23] R. M. Torrente-Rodríguez, H. Lukas, J. Tu, J. Min, Y. Yang, C. Xu, H. B. Rossiter, W. Gao, *Matter* **2020**, *3*, 1981.

[24] A. Idili, C. Parolo, R. Alvarez-Diduk, A. Merkoçi, *ACS Sensors* **2021**, *6*, 3093.

[25] L. F. de Lima, A. L. Ferreira, M. D. T. Torres, W. R. de Araujo, C. de la Fuente-Nunez, *Proc. Natl. Acad. Sci. U. S. A.* **2021**, *118*, DOI 10.1073/pnas.2106724118.

[26] M. A. Zamzami, G. Rabbani, A. Ahmad, A. A. Basalah, W. H. Al-Sabban, S. Nate Ahn, H. Choudhry, *Bioelectrochemistry* **2022**, *143*, 107982.

[27] Z. Zhang, R. Pandey, J. Li, J. Gu, D. White, H. D. Stacey, J. C. Ang, C. J. Steinberg, A. Capretta, C. D. M. Filipe, K. Mossman, C. Balion, M. S. Miller, B. J. Salena, D. Yamamura, L. Soleymani, J. D. Brennan, Y. Li, *Angew. Chemie - Int. Ed.* **2021**, *60*, 24266.

[28] D. Beduk, J. Ilton de Oliveira Filho, T. Beduk, D. Harmanci, F. Zihnioglu, C. Cicek, R. Sertoz, B. Arda, T. Goksel, K. Turhan, K. N. Salama, S. Timur, *Biosens. Bioelectron. X* **2022**, *10*, 100105.

[29] H. Liu, A. Yang, J. Song, N. Wang, P. Lam, Y. Li, H. K. W. Law, F. Yan, *Sci. Adv.* **2021**, *7*, 8387.

[30] *COVID-19 Ag Rapid Test Device 41FK11/41FK21 In Vitro Diagnostic Rapid Test for Qualitative Detection of SARS-CoV-2 Antigen (Ag) (NASAL).*

[31] Access Bio Inc. CareStart COVID-19 Antigen Home Test [package insert]. U.S. Food and Drug Administration website. https://www.fda.gov/media/142919/download. Revised April 2021.

[32] OraSure Technologies Inc. InteliSwab COVID-19 Rapid Test Rx [package insert]. U.S. Food and Drug Administration website. https://www.fda.gov/media/149906/download Revised January 2022.

[33] LumiraDx, "The LumiraDx SARS-CoV-2 Ag Test is a rapid microfluidic immunoassay detecting SARS-CoV-2 antigen," **2020**.

[34] BTNX Inc. Rapid Response COVID-19 Antigen Rapid Test Device [package insert]. U.S. Food and Drug Administration website. https://www.btnx.com/files/1110032811V5_COVID-19_Antigen_Rapid_Test_Device.pdf. Revised November 2020.

[35] Access Bio, Inc. Access Bio CareStart COVID-19 IgM/IgG [package insert]. U.S. Food and Drug Administration website. https://www.fda.gov/media/140447/download. Revised June 2021..

[36] Abbott Ireland Diagnositics Division, *AdviseDx SARS-CoV-2 IgG II Instructions For Use*, **2021**.

[37] Siemens, *EliA SARS-CoV-2-Sp1 IgG Test - IFU | FDA*, **2021**.

[38] Kantaro Biosciences, "COVID-SeroKlir, Kantaro Semi-Quantitative SARS-CoV-2 IgG Antibody Kit," **2020**.

[39] H. F. Florindo, R. Kleiner, D. Vaskovich-Koubi, R. C. Acúrcio, B. Carreira, E. Yeini, G. Tiram, Y. Liubomirski, R. Satchi-Fainaro, *Nat. Nanotechnol. 2020 158* **2020**, *15*, 630.

[40] R. Phillips, J. Kondev, J. Theriot, H. G. Garcia, N. Orme, *Physical Biology of the Cell*, Garland Science, **2012**.

[41] H. R. Culver, N. A. Peppas, *Chem. Mater.* **2017**, *29*, 5753.

[42] J. Porter, J. Berkhahn, L. Zhang, in *Emerg. Trends Comput. Biol. Bioinformatics, Syst. Biol. Algorithms Softw. Tools*, Morgan Kaufmann, **2015**, pp. 521–535.

[43] A. C. Walls, Y. J. Park, M. A. Tortorici, A. Wall, A. T. McGuire, D. Veesler, *Cell* **2020**, *181*, 281.

[44] M. H. Afrad, M. H. Khan, S. I. A. Rahman, O. H. Bin Manjur, M. Hossain, A. N. Alam, F. I. Khan, N. Afreen, F. T. Haque, N. R. Thomson, T. Shirin, F. Qadri, *Microbiol. Resour. Announc.* **2021**, *10*, DOI 10.1128/mra.00560-21.

[45] S. Tsuchiya, N. P. Aoki, D. Shinmachi, M. Matsubara, I. Yamada, K. F. Aoki-Kinoshita, H. Narimatsu, *Carbohydr. Res.* **2017**, *445*, 104.

[46] S. M. C. Gobeil, K. Janowska, S. McDowell, K. Mansouri, R. Parks, V. Stalls, M. F. Kopp, K. Manne, D. Li, K. Wiehe, K. O. Saunders, R. J. Edwards, B. Korber, B. F.

Haynes, R. Henderson, P. Acharya, *Science* **2021**, *373*, DOI 10.1126/SCIENCE.ABI6226.

[47] A. J. Venkatakrishnan, P. Anand, P. J. Lenehan, R. Suratekar, B. Raghunathan, M. J. M. Niesen, V. Soundararajan, **2021**, DOI 10.31219/OSF.IO/F7TXY.

[48] D. Mannar, J. W. Saville, X. Zhu, S. S. Srivastava, A. M. Berezuk, K. S. Tuttle, C. Marquez, I. Sekirov, S. Subramaniam, *bioRxiv* **2021**, 2021.12.19.473380.

[49] M. Gui, W. Song, H. Zhou, J. Xu, S. Chen, Y. Xiang, X. Wang, *Cell Res.* **2017**, *27*, 119.

[50] Z. Li, A. C. A. Tomlinson, A. H. M. Wong, D. Zhou, M. Desforges, P. J. Talbot, S. Benlekbir, J. L. Rubinstein, J. M. Rini, *Elife* **2019**, *8*, DOI 10.7554/eLife.51230.

[51] Y. Yuan, D. Cao, Y. Zhang, J. Ma, J. Qi, Q. Wang, G. Lu, Y. Wu, J. Yan, Y. Shi, X. Zhang, G. F. Gao, *Nat. Commun. 2017 81* **2017**, *8*, 1.

[52] S. J. Gamblin, L. F. Haire, R. J. Russell, D. J. Stevens, B. Xiao, Y. Ha, N. Vasisht, D. A. Steinhauer, R. S. Daniels, A. Elliot, D. C. Wiley, J. J. Skehel, *Science (80-.).* **2004**, *303*, 1838.

[53] S. J. Perkins, A. S. Nealis, B. J. Sutton, A. Feinstein, *J. Mol. Biol.* **1991**, *221*, 1345.

[54] L. J. Harris, E. Skaletsky, A. McPherson, *J. Mol. Biol.* **1998**, *275*, 861.

[55] B. Ju, Q. Zhang, J. Ge, R. Wang, J. Sun, X. Ge, J. Yu, S. Shan, B. Zhou, S. Song, X. Tang, J. Yu, J. Lan, J. Yuan, H. Wang, J. Zhao, S. Zhang, Y. Wang, X. Shi, L. Liu, J. Zhao, X. Wang, Z. Zhang, L. Zhang, *Nature* **2020**, *584*, 115.

[56] S. Kang, M. Yang, S. He, Y. Wang, X. Chen, Y. Q. Chen, Z. Hong, J. Liu, G. Jiang, Q. Chen, Z. Zhou, Z. Zhou, Z. Huang, X. Huang, H. He, W. Zheng, H. X. Liao, F. Xiao, H. Shan, S. Chen, *Nat. Commun.* **2021**, *12*, 1.

[57] Z. Chen, L. Bao, C. Chen, T. Zou, Y. Xue, F. Li, Q. Lv, S. Gu, X. Gao, S. Cui, J. Wang, C. Qin, Q. Jin, *J. Infect. Dis.* **2017**, *215*, 1807.

[58] K. R. McCarthy, J. Lee, A. Watanabe, M. Kuraoka, L. R. Robinson-Mccarthy, G. Georgiou, G. Kelsoe, S. C. Harrison, *MBio* **2021**, *12*, DOI 10.1128/mBio.01144-21.

[59] R. Sender, R. Milo, *Nat. Med. 2021 271* **2021**, *27*, 45.

[60] C. B. P. Ligiero, T. S. Fernandes, D. L. D'Amato, F. V. Gaspar, P. S. Duarte, M. A. Strauch, J. G. Fonseca, L. G. R. Meirelles, P. Bento da Silva, R. B. Azevedo, G. Aparecida de Souza Martins, B. S. Archanjo, C. D. Buarque, G. Machado, A. M. Percebom, C. M. Ronconi, *Mater. Today Chem.* **2022**, *25*, 100924.

[61] H. Kang, X. Wang, M. Guo, C. Dai, R. Chen, L. Yang, Y. Wu, T. Ying, Z. Zhu, D. Wei, Y. Liu, D. Wei, *Nano Lett.* **2021**, *21*, 7897.

[62] A. N. Masterson, B. B. Muhoberac, A. Gopinadhan, D. J. Wilde, F. T. Deiss, C. C. John, R. Sardar, *Anal. Chem.* **2021**, *93*, 8754.

[63] J. McClements, L. Bar, P. Singla, F. Canfarotta, A. Thomson, J. Czulak, R. E. Johnson, R. D. Crapnell, C. E. Banks, B. Payne, S. Seyedin, P. Losada-Pérez, M. Peeters, *ACS Sensors* **2022**, *7*, 1122.

[64] M. A. Tabrizi, J. P. Fernández-Blázquez, D. M. Medina, P. Acedo, *Biosens. Bioelectron.* **2022**, *196*, 956.

[65] Z. Bognár, E. Supala, A. Yarman, X. Zhang, F. F. Bier, F. W. Scheller, R. E. Gyurcsányi, *Chem. Sci.* **2022**, *13*, 1263.

[66] J. Bhardwaj, N. Chaudhary, H. Kim, J. Jang, *Anal. Chim. Acta* **2019**, *1064*, 94.

[67] C. Bai, Z. Lu, H. Jiang, Z. Yang, X. Liu, H. Ding, H. Li, J. Dong, A. Huang, T. Fang, Y. Jiang, L. Zhu, X. Lou, S. Li, N. Shao, *Biosens. Bioelectron.* **2018**, *110*, 162.

[68] S. C. B. Gopinath, P. K. R. Kumar, *Acta Biomater.* **2013**, *9*, 8932.

[69] I. Shiratori, J. Akitomi, D. A. Boltz, K. Horii, M. Furuichi, I. Waga, *Biochem. Biophys. Res. Commun.* **2014**, *443*, 37.

[70] D. D. Raymond, G. Bajic, J. Ferdman, P. Suphaphiphat, E. C. Settembre, M. A. Moody, A. G. Schmidt, S. C. Harrison, *Proc. Natl. Acad. Sci. U. S. A.* **2018**, *115*, 168.

[71] S. Lefever, C. Indevuyst, L. Cuypers, K. Dewaele, N. Yin, F. Cotton, E. Padalko, M. Oyaert, J. Descy, E. Cavalier, M. Van Ranst, E. André, K. Lagrou, P. Vermeersch, *J. Clin. Microbiol.* **2021**, *59*, DOI 10.1128/JCM.00374-21.

[72] A. S. Walker, E. Pritchard, T. House, J. V. Robotham, P. J. Birrell, I. Bell, J. I. Bell, J. N. Newton, J. Farrar, I. Diamond, R. Studley, J. Hay, K. D. Vihta, T. E. A. Peto, N. Stoesser, P. C. Matthews, D. W. Eyre, K. B. Pouwels, *Elife* **2021**, *10*, DOI 10.7554/eLife.64683.

[73] M. Brandolini, F. Taddei, M. M. Marino, L. Grumiro, A. Scalcione, M. E. Turba, F. Gentilini, M. Fantini, S. Zannoli, G. Dirani, V. Sambri, *Viruses* **2021**, *13*, DOI 10.3390/v13061022.

[74] N. R. Pollock, T. J. Savage, H. Wardell, R. A. Lee, A. Mathew, M. Stengelin, G. B. Sigal, *J. Clin. Microbiol.* **2021**, *59*, DOI 10.1128/JCM.03077-20.
